# Trends and cross-country inequalities in the global burden of gastrointestinal cancers attributable to smoking, 1990–2021: A secondary dataset analysis of GBD Study 2021

**DOI:** 10.18332/tid/217692

**Published:** 2026-06-12

**Authors:** Jian Yang, Chengqing Yu, Wenxu Cui, Junwei Ji, Jiazi Hao, Jun Yao, Ye Li, Jian Zhou

**Affiliations:** 1 Department of General Surgery, The First Affiliated Hospital of Soochow University, Suzhou, China; 2 State Key Laboratory of Radiation Medicine and Protection, Soochow University, Suzhou, China; 3 Department of General Surgery, The Fourth Affiliated Hospital of Soochow University, Suzhou, China

**Keywords:** gastrointestinal cancers, GBD study, disability-adjusted life years, health inequality

## Abstract

**INTRODUCTION:**

Gastrointestinal (GI) cancers, including esophageal, gastric, and colorectal cancers, are major contributors to the global cancer burden. Epidemiological trends of GI cancers attributable to smoking have not been comprehensively examined. We aim to evaluate the burden of GI cancers attributable to smoking and its cross-country inequalities from 1990 to 2021.

**METHODS:**

A secondary assessment of Global Burden of Disease (GBD) datasets was conducted. Deaths, age-standardized mortality rate (ASMR), disability-adjusted life years (DALYs), age-standardized rate of DALYs (ASDR), and estimated annual percentage changes (EAPCs) were obtained from the GBD 2021. Spearman's rank test and a locally weighted regression (LOESS) model were utilized as analytical models. We quantified the inequalities using the slope index of inequality (SII) and the concentration index (Crl) associated with the national sociodemographic index.

**RESULTS:**

In 2021, the ASMR for smoking-attributable esophageal, gastric, and colorectal cancers was 2.38 (95% UI: 1.81–2.98), 1.25 (95% UI: 0.98–1.61), and 0.55 (95% UI: 0.34–0.77) per 100000 persons, respectively. From 1990 to 2021, there was a downward trend in the ASMRs and ASDRs for all three GI cancers attributed to smoking. The distribution and trajectory of the burden of disease exhibits considerable variation across regions. The three GI cancers had positive SIIs and Crls, with their Crls changing from 0.03 to 0.21 (esophageal cancer), 0.12 to 0.19 (gastric cancer), and 0.32 to 0.28 (colorectal cancer) between 1990 and 2021, suggesting that the disease burden is more prevalent and concentrated in countries with higher SDIs. However, heterogeneity was observed in the changes of absolute and relative inequality of the three GI cancers attributable to smoking between 1990 and 2021.

**CONCLUSIONS:**

The burden of GI cancer associated with smoking has increased in some regions from 1990 to 2021. These differing regional patterns may suggest a potential need for further assessment or targeted interventions in some areas.

## INTRODUCTION

Cancer is a substantial global public health challenge, with persistently rising incidence and mortality rates. According to GLOBOCAN Cancer Statistics 2022, there are 36 malignant tumors in 185 countries or territories worldwide, with approximately 19965000 new cases and 9377000 deaths^1^. Gastrointestinal (GI) cancers, including esophageal, gastric, and colorectal cancers, account for 39.29% of all cancer-related deaths^2^. Collectively, these malignancies account for approximately one-third of new cancer cases and are ranked seventh, fifth, and second in terms of mortality rates, respectively^1^. Esophageal, gastric, and colorectal cancers present distinctive features and commonalities. First, the majority of GI cancers exhibit analogous premalignant stages, characterized by chronic inflammation, epithelial hyperplasia, or atypical hyperplasia, which can progress to malignancy. Second, environmental factors or detrimental lifestyle habits, such as smoking, alcohol consumption, protracted stress, and psychological distress, play pivotal roles in the development of GI cancer. In clinical practice, all three GI cancers are highly amenable to gastrointestinal endoscopy, which plays a crucial role in early screening, diagnosis, and treatment in high-risk populations.

The tobacco epidemic constitutes one of the most significant public health challenges confronting the global community. In 2019, more than 1 billion individuals were estimated to be regular smokers. This will result in approximately 8 million deaths and 200 million disability-adjusted life years (DALYs)^3^. Tobacco smoke comprises at least 60 compounds identified as carcinogenic. In addition to the direct effects of these carcinogenic compounds, the increased inflammation induced by smoking contributes to an increased risk of cancer^4^. Smoking constitutes a considerable risk factor for the development of cancerous diseases, including the trachea, bronchus, lung, esophagus, stomach, colorectum, pancreas, kidney, bladder, and cervix^5,6^. Smoking is a modifiable behavioral risk factor, and accumulating evidence suggests that further interventions involving relevant environmental and behavioral factors should be incorporated into the prevention, diagnosis, and treatment of GI cancers.

Previous studies have reported significant heterogeneity in the disease burden of cancers across geographical locations and sociodemographic index (SDI) levels^7-9^. Therefore, obtaining accurate data on GI cancers is essential for the management of patients, the implementation of early cancer screening, the creation of public health policies, and the advancement of national cancer control programs. We used data from the Global Burden of Disease (GBD) 2021 database to analyze the epidemiological trends, patterns, and changes in mortality and DALY characteristics of these three cancers due to smoking, with an aim to better understand regional differences and provide new insights into cancer prevention and public health^10^. This study aimed to examine global patterns and socioeconomic inequalities in smoking-attributable gastrointestinal cancers across regions between 1990 and 2021.

## METHODS

### Data sources

This study is a secondary analysis of GBD 2021 data^10^. In accordance with our research objectives, the inclusion criteria were patients with esophageal cancer, gastric cancer, and colorectal cancer, with smoking as the exposure factor. GBD 2021 comprehensively assessed the health burden caused by 371 diseases, injuries, and hazards, as well as 88 risk factors, across 204 countries and regions worldwide from 1990 to 2021. The study is implemented by the Institute for Health Metrics and Evaluation (IHME) and adheres to the Guidelines for Accurate and Transparent Reporting of Health Estimates (GATHER) statement^11^. The global burden of disease due to smoking was extracted from GBD 2021 using the Global Health Data Exchange (GHDx) query tool^10^. The dataset encompasses the number of smoking-related deaths, DALYs, age-standardized mortality rates (ASMRs), and age-standardized disability-adjusted life year rates (ASDRs) for 21 GBD regions and 204 countries/territories for each year between 1990 and 2021. The analysis was conducted in accordance with GATHER, and all information on ethical standards is available through the official GBD website^12^. The authors confirm that they have complied with this ethics policy. As this study was based on secondary analysis of publicly available data, no additional human participant research ethics review or informed consent was needed.

### Definitions

According to the International Classification of Diseases, 10th edition (ICD-10)^13^, esophageal cancer is coded as C15-C15.9, gastric cancer is coded as C16-C16.9, and colorectal cancer is coded as C18-20 and C21-C21.8. The term ‘smoking exposure’ is defined as the use of any smoking product on a daily or occasional basis, as well as a person who has used any smoking product in the past. All smoking products were included, such as cigarettes, pipes, cigars, and hookahs. Smokeless tobacco, electronic cigarettes, vaporized products, and heated tobacco products were not included^3^. The SDI is a novel development classification index developed by the GBD for countries as a proxy for the human development index (HDI). The SDI is calculated on the basis of the lag in per capita distribution of income, the average education level of the population aged ≥15 years, and the total fertility rate up to the age of 25 years. The SDI was created to divide 204 countries into five regions: high (>0.81), high-middle (0.70–0.81), middle (0.61–0.69), low-middle (0.46–0.60), and low (<0.46) SDIs, ranging from 0 to 1^14,15^. Furthermore, with consideration for socioeconomic similarities and geographical proximity, the 204 countries and territories were subsequently classified into 21 GBD regions.

### Measures of health inequality

The assessment of absolute and relative income-related inequality between countries followed the recommendations of the World Health Organization (WHO). Two standard measures were used to achieve this objective: the slope index of inequality (SII) and the concentration index (CrI)^16^. Absolute inequality across countries is calculated using a robust linear regression model. The SII represents the slope of the regression line that relates the crude disability-adjusted life-year rate (CDR) to the weighted ranking of each country. Furthermore, an analysis of health inequalities in gastrointestinal cancers between countries from 1990 to 2021 was conducted using concentration curves and concentration indices. Concentration curves plot the unequal distribution of the cumulative portion of DALYs against the cumulative proportion of countries ranked by their level of socioeconomic development^17,18^. Health outcome variables are more prevalent in low-income countries when the curve lies above the line of equality (the 45-degree line), and vice versa. The CrI is derived from the concentration curve, which quantifies relative socioeconomic inequalities in health and is equal to twice the area between the concentration curve and the equality line. The CrI ranges from -1 to 1, where negative values indicate that ASDRs are more concentrated in low-income countries, whereas positive values indicate that the burden of disease is concentrated in high-income countries.

### Statistical analysis

The data on deaths, DALYs, the ASMR, and the ASDR are reported as numbers and 95% uncertainty intervals (UIs), which are generated using 1000 sampled values ordered at the 2.5th and 97.5th percentiles of the posterior distribution. An estimated annual percentage change (EAPC) was calculated to quantify the secular trends of the ASMR and ASDR from 1990 to 2019 through the following formula: ln(y) = α+βx+ε, where y represents the ASMR or ASDR and x represents the calendar year. The EAPC and its 95% confidence interval (CI) were calculated as 100×[exp (β)-1]. When the 95% CI of the corresponding EAPC estimate exceeds 0, it suggests an increasing ASMR or ASDR. On the other hand, if the 95% CI falls below 0, the ASMR or ASDR decreases. Finally, a 95% CI that includes zero denotes a stabilizing ASMR or ASDR. In addition, the correlation of the SDI with the ASDR and ASMR was assessed by Spearman’s rank test, and the expected relationship between them was derived by a locally weighted regression (LOESS) model. All data are expressed as values, and their 95% CIs or UIs, and rates are reported per 100000 persons. All other statistical analyses were performed using R software (version 4.3.2)^19^, and a p<0.05 was considered statistically significant.

## RESULTS

### Burdens at the global level

Worldwide, 205463 deaths from esophageal cancer were attributable to smoking in 2021, an increase of 69013 cases from 1990, with an ASMR of 2.38 (95% UI: 1.81– 2.98) and an ASDR of 54.26 (95% UI: 41.29–68.02) per 100000 people. The ASMR of gastric cancer attributable to smoking was 1.25 (95% UI: 0.98–1.61), and the ASDR was 29.01 (95% UI: 22.75–37.32). For smoke-related colorectal cancer, the ASMR was 0.55 (95% UI: 0.34–0.77), and the ASDR was 14.12 (95% UI: 8.84–19.5) per 100000 people. Compared with those in 1990, there were decreases in the ASMRs and ASDRs for all three GI cancers in 2021 (Table 1). The global EAPCs for the ASMR and ASDR for esophageal cancer attributable to smoking from 1990 to 2021 were -1.38 (95% CI: -1.49 – -1.27) and -1.76 (95% CI: -1.87 – -1.65), respectively. The EAPC for gastric cancer was -2.63 (95% CI: -2.68 – -2.58) for the ASMR and -2.94 (95% CI: -2.99 – -2.89) for the ASDR. For colorectal cancer, the EAPCs were -1.26 (95% CI: -1.29 – -1.24) for the ASMR and -1.28 (95% CI: -1.31 – -1.26) for the ASDR. The results revealed a significant downward trend in the ASMRs and ASDRs for all three GI cancers attributed to smoking worldwide (Table 1).

**Table 1:** Global burden of three GI cancers attributed to smoking in 1990 and 2021, and the temporal trends from 1990 to 2021

*Year*	*Esophageal cancer*	*Gastric cancer*	*Colorectal cancer*
**1990**			
Deaths (95% UI)	136450 (109956–163495)	109818 (89441–131513)	31090 (19792–42082)
ASMR per 100000 (95% UI)	3.46 (2.79–4.13)	2.81 (2.29–3.36)	0.80 (0.51–1.09)
DALYs (95% UI)	3640952 (2931006–4386515)	2929437 (2380225–3509272)	850250 (541714–1141631)
ASDR per 100000 (95% UI)	88.47 (71.26–106.51)	71.17 (58.04–85.11)	20.64 (13.14–27.74)
**2021**			
Deaths (95% UI)	205463(156544–257209)	107926 (84603–138448)	47613 (29670–66040)
ASMR per 100000 (95% UI)	2.38 (1.81–2.98)	1.25 (0.98–1.61)	0.55 (0.34–0.77)
DALYs (95% UI)	4765032 (3626148–5973195)	2537998 (1991161–3270229)	1235667 (773606–1708237)
ASDR per 100000 (95% UI)	54.26 (41.29––68.02)	29.01 (22.75–37.32)	14.12 (8.84–19.50)
**1990–2021**			
EAPC of ASMR (95% CI)	-1.38 (-1.49 – -1.27)	-2.63 (-2.68 – -2.58)	-1.26 (-1.29 – -1.24)
EAPC of ASDR (95% CI)	-1.76 (-1.87 – -1.65)	-2.94 (-2.99 – -2.89)	-1.28 (-1.31 – -1.26)

GI: gastrointestinal. DALYs: disability-adjusted life years. ASMR: age-standardized mortality rates. ASDR: age-standardized DALYs rates. EAPC: estimated annual percentage change.

### The burden at the SDI level

In all SDI regions, the ASMR and ASDR for esophageal cancer attributable to smoking increased with increasing SDI from low-SDI regions to high-middle-SDI regions. The ASMR increased from 0.59/100000 (95% UI: 0.45–0.74) in low-SDI regions to 3.7/100000 (95% UI: 2.77–4.83) in high-middle-SDI regions. ASDR increased from 14.79/100000 (95% UI: 11.21–18.59) in low-SDI regions to 86.54/100000 (95% UI: 64.83–113.38) in high-middle-SDI regions. Compared with those in the high-middle-SDI area, the ASMR and ASDR in the high-SDI area decreased slightly to 1.65 per 100000 (95% UI: 1.29–2.0) and 37.57 per 100000 (95% UI: 29.85–45.36), respectively (Supplementary file Table S1). Similarly, the ASMR and ASDR for gastric and colorectal cancers attributable to smoking increased as the SDI increased from low-SDI regions to high-middle-SDI regions, and the ASMR and ASDR decreased slightly in high-SDI regions relative to high-middle-SDI regions (Supplementary file Tables S2 and S3). These findings indicate that the three types of GI cancers attributed to smoking have similar trends in the SDI regions.

### Burdens at the regional level

Among the 21 GBD regions, the ASMR and ASDR for esophageal cancer attributable to smoking were highest in East Asia at 6.44 (95% UI: 4.76–8.4) and 143.42 (95% UI: 105.65–188.21) per 100000 people and lowest in Andean Latin America at 0.17 (95% UI: 0.12–0.24) and 3.7 (95% UI: 2.6–5.09) per 100000 people, respectively (Supplementary file Table S1). Similarly, the ASMR and ASDR for gastric cancer attributable to smoking were highest in East Asia, at 3.04 (95% UI: 2.24–4.21) and 70 (95% UI: 51.49–97), respectively. Conversely, the lowest values were observed in western Sub-Saharan Africa, with ASMRs and ASDRs of 0.14 (95% UI: 0.1–0.18) and 3.54 (95% UI: 2.5–4.53), respectively (Supplementary file Table S2). The ASMR and ASDR for colorectal cancer attributable to smoking were highest in Central Europe at 1.12 (95% UI: 0.71–1.56) and 30.13 (95% UI: 19.01–41.72), respectively, and lowest in Western Sub-Saharan Africa at 0.07 (95% UI: 0.04–0.10) and 1.66 (95% UI: 0.98– 2.38), respectively (Supplementary file Table S3).

In the 21 GBD regions, the ASMR and ASDR of esophageal cancer attributable to smoking exhibited a marked upward trend in western Sub-Saharan Africa, with EAPCs of 1.52 (95% CI: 1.34–1.71) and 1.51 (95% CI: 1.33–1.69), respectively. Conversely, the EAPCs in other regions were <0, suggesting a downward trend in both the ASMR and ASDR (Supplementary file Table S1). The EAPC for gastric cancer attributed to smoking exhibited a significant downward trend in both the ASMR and ASDR across the 21 GBD regions (Supplementary file Table S2). For colorectal cancer, the EAPC values of the ASMR and ASDR in Central Sub-Saharan Africa were 0, indicating a stable trend. Southeast Asia and Andean Latin America presented significant increasing trends in the ASDR and ASMR, whereas the other regions presented significant decreasing trends (Supplementary file Table S3).

### The burden at the national level

**Figure 1:**
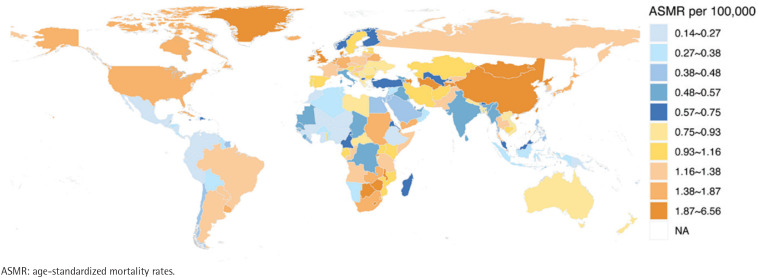
Spatial distribution and ASMR of smoking-related esophageal cancer in 204 countries and territories in 2021

**Figure 2:**
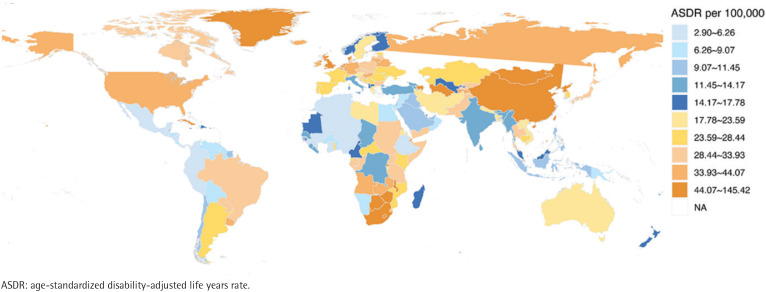
Spatial distribution and ASDR of smoking-related esophageal cancer in 204 countries and territories in 2021

**Figure 3:**
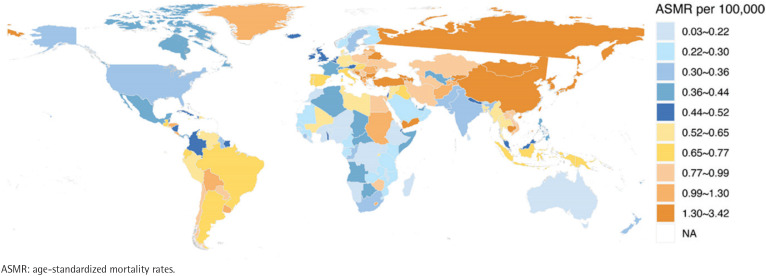
Spatial distribution and ASMR of smoking-related gastric cancer in 204 countries and territories in 2021

**Figure 4:**
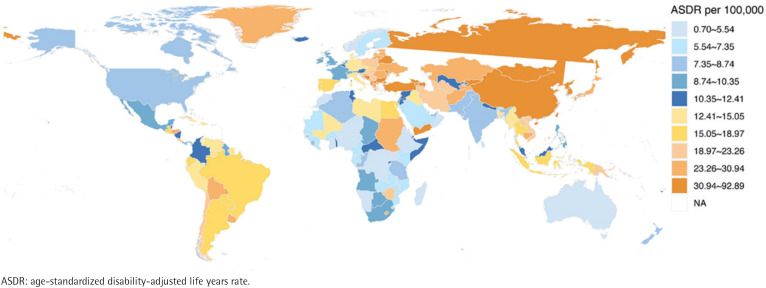
Spatial distribution and ASDR of smoking-related gastric cancer in 204 countries and territories in 2021

At the national level, the ASMR for esophageal cancer attributable to smoking in 2021 ranged from 0.14 to 6.56 per 100000 (Figure 1). The country with the lowest rate was the Republic of Peru, whereas the highest rate was recorded in the People’s Republic of China. The ASDR ranged from 2.90 per 100000 to 145.42 per 100000 (Figure 2). The Republic of Peru had the lowest rate, whereas the People’s Republic of China had the highest rate (Supplementary file Table S4). The ASMR for gastric cancer attributable to smoking in 2021 ranged from 0.03 to 3.42 per 100000 individuals (Figure 3). The ASDR ranged from 0.70 to 92.89 per 100000 individuals (Figure 4). The lowest and highest levels are observed in the Federal Republic of Nigeria and Mongolia (Supplementary file Table S4). For colorectal cancer, the ASMR and ASDR range from 0.04 per 100000 to 1.55 per 100000 and from 1.01 per 100000 to 42.29 per 100000, respectively (Supplementary file Figures S0A and S0B). The lowest country was the Federal Republic of Nigeria, and the highest country was Greenland (Supplementary file Table S4).

A downward trend was observed in the ASMRs and ASDRs for esophageal cancer attributable to smoking from 1990 to 2021 in most countries (Supplementary file Figures S1A and S1B). The EAPC for the ASMR ranged from -539% to 370%. The country with the most significant downward trend in the ASMR was the Republic of Colombia, followed by the Democratic Republic of Sao Tome and Principe, which exhibited the most significant upward trend (Supplementary file Table S5). A decline in the ASMR and ASDR for gastric cancer attributable to smoking was observed in most countries (Supplementary file Figures S1C and S1D). However, an increasing trend in ASMR was noted in the Arab Republic of Egypt, the Kingdom of Lesotho, Georgia, and the Republic of Mali. Furthermore, an increase in the ASDR was observed in three countries: the Arab Republic of Egypt, the Kingdom of Lesotho, and the Republic of Mali (Supplementary file Table S5). A decreasing trend was observed in the ASMR and ASDR for colorectal cancer attributable to smoking (Supplementary file Figures S1E and S1F). Approximately 63.25% of the countries exhibited a decline in the ASMR, whereas approximately 64.22% of the countries demonstrated a decrease in the ASDR. However, approximately 28.4% of countries exhibited an increasing trend in the ASMR, and 25.98% of countries demonstrated an increasing trend in the ASDR (Supplementary file Table S5).

### Trends in burden by sex across SDI regions

From 1990 to 2021, there was a clear upward trend in the number of male esophageal cancer deaths and the number of DALYs attributable to smoking across all five SDI regions. However, within the low- and low-middle-SDI regions, there was an increasing trend in the number of female deaths and the number of years with DALYs. Moreover, in the other three regions, there was an initial increasing trend, followed by a decrease (Supplementary file Figures S2A and S2B). The burden of gastric cancer attributable to smoking showed a downward trend in the number of male deaths and DALYs in the high-SDI region, while a downward trend was also observed in the number of female deaths and DALYs in the high-SDI, high-middle, and middle regions (Supplementary file Figures S2C and S2D). The burden of colorectal cancer attributable to smoking exhibited an increasing trend in the number of male deaths and DALYs across SDI regions, a decreasing trend in the number of female deaths and DALYs in the high-SDI region, and an increasing trend in the remaining regions (Supplementary file Figures S2E and S2F). In general, males presented significantly higher mortality rates, ASMRs, DALYs, and ASDRs than females did (Supplementary file Tables S6–S17). Moreover, a global downward trend was observed in the ASMRs and ASDRs for all three GI cancers attributable to smoking from 1990 to 2021, affecting both males and females. However, the decline was particularly pronounced in female populations.

### Associations between disease burden and the SDI

A correlation analysis was conducted on the burden of disease with the SDI in 21 regions from 1990 to 2021. The analysis revealed significant correlations between the SDI and the ASMR or ASDR for three GI cancers caused by smoking. In both esophageal and gastric cancers, a positive correlation was observed between the SDI and the ASMR, with overall correlation coefficient (ρ) values of 0.440 and 0.352, respectively. A similar trend was noted for the SDI and the ASDR (Supplementary file Figures S3A–S3D). In East Asia, although the ASMR and ASDR continue to decline, they are still well above the expected levels throughout the year. In colorectal cancer, the SDI was strongly positively correlated with the ASMR and ASDR, with ρ values of 0.762 and 0.765, respectively. The disease burden was highest when the SDI was approximately 0.74 and then decreased (Supplementary file Figures S3E and S3F). As the SDI increases, the ASMR and ASDR show a decreasing trend in the high-income regions of North America, Australia, and the high-income countries in the Asia-Pacific region, and all of them are close to or below the expected levels.

A comprehensive analysis encompassing 204 countries and territories revealed a substantial correlation between the SDI and the ASMR, as well as the ASDR, for GI cancers attributable to smoking. In the case of esophageal and gastric cancers, the SDI exhibited a weak positive correlation with the ASMR and ASDR (Supplementary file Figures S4A–S4D). The observed levels were considerably higher than expected in certain countries, including China and Mongolia in East Asia. Conversely, some countries, such as Singapore, presented levels significantly below expectations. In colorectal cancer, the SDI demonstrated a robust positive association with the ASMR and ASDR, with ρ values of 0.642 and 0.644, respectively. As the SDI increased, the disease burden demonstrated a positive correlation, peaking at approximately 0.77 and subsequently declining (Supplementary file Figures S4E and S4F).

### Cross-national health inequality

The SII shows a positive correlation between DALYs and the SDI index, with a disproportionate concentration of DALYs in countries with a high level of sociodemographic development. In 1990 and 2021, the SIIs for esophageal cancer DALYs attributable to smoking were 36.20 (95% CI: 27.48–44.91) and 34.67 (95% CI: 27.45–41.88), respectively (Supplementary file Figure S5A). A similar trend was observed for gastric cancer, with an SII of 47.20 (95% CI: 38.26–56.13) in 1990 and 21.49 (95% CI: 16.39–26.59) in 2021 (Supplementary file Figure S5C). A decrease in the SII indicates an improvement in the inequality in the burden of disease between high-income and low-income countries during this period. In contrast, the SII for colorectal cancer increased from 25.34 (95% CI: 20.90–29.78) in 1990 to 27.61 (95% CI: 23.89–31.33) in 2021, suggesting an increase in absolute inequality in disease burden (Supplementary file Figure S5E).

The increase in the esophageal cancer concentration index from 0.03 in 1990 to 0.21 in 2021 indicates that the relative inequality in the burden of disease between poor and rich countries has widened regionally (Supplementary file Figure S5B). This finding indicates that, despite advancements in overall health, the disparity in health status between the rich and the poor has widened. A similar trend was observed in the case of gastric cancer (Supplementary file Figure S5D). The colorectal cancer concentration index decreased from 0.32 to 0.28 (Supplementary file Figure S5F), indicating that relative inequality also improved.

## DISCUSSION

Cancers of the gastrointestinal tract are a major cause of cancer-related morbidity and mortality on a global scale^20^. This study indicates that global and regional trends in the burden and inequality of the three types of GI cancers caused by smoking have undergone significant changes over the past three decades. The findings revealed a decline in the trends of the ASMRs and ASDRs for all three categories of GI cancers attributable to smoking. This observation signifies that the present strategies for cancer prevention and treatment are efficacious. A multitude of efficacious tobacco control programs and policies have achieved substantial reductions in tobacco use in recent decades. These include, but are not limited to, the imposition of taxes on tobacco products, the prohibition of smoking in public places, and the implementation of the World Health Organization Framework Convention on Tobacco Control (WHO FCTC)^21,22^. Despite the global decline in smoking prevalence, the absolute number of smokers is increasing due to population growth. Compared with the data from 1990, there was a nearly 50% increase in both the absolute number of deaths and DALYs from esophageal and colorectal cancer attributable to smoking in 2021. This increase can be partially attributed to the growth and aging of the smoking population.

Tobacco smoke contains many carcinogens that can cause a variety of cancers. Nicotine, a major carcinogen in tobacco products, has been observed to down-regulate OTUD3 and ZFP36, thereby preventing the decay of VEGF-C mRNA, which in turn promotes lymphatic metastasis of esophageal cancer cells^23^. In the colon, inhaled smoke has been demonstrated to induce dysbiosis of the intestinal microbiota, alterations in intestinal metabolites, and weakening of the intestinal barrier. These phenomena may contribute to carcinogenic transformation of the intestinal epithelium^24^. These mechanisms depend on individual genetic susceptibility and the stability of the intestinal microenvironment. Recent research employing a prospective cohort-tumor biobank methodology (PCIBM) demonstrated a stronger association of smoking with colorectal cancer incidence for tumors containing higher numbers of exome-wide somatic mutations. Smoking may contribute to the development of colorectal tumors, especially those with high frequencies of somatic mutations, possibly through its effect on the tumor immune microenvironment^25^.

The three GI cancer burdens attributable to smoking exhibited a certain degree of similarity with respect to the SDI. The results demonstrated that within the SDI regions, both the ASMR and ASDR tended to increase as the SDI level increased from low-SDI regions to high-middle-SDI regions. It was reported that tobacco exposure has remained high in high-middle-SDI regions, including high-income Asia-Pacific, Europe, and East Asia^26^. Although these regions typically possess strong healthcare infrastructure and comprehensive health education, tobacco exposure remains substantial. These findings may reflect the influence of a range of non-economic factors on smoking patterns, although this cannot be directly evaluated in the present analysis. These factors may include physiological elements, among others^27^. The relatively lower burden in low-SDI regions may be partly due to lower smoking prevalence and an underdeveloped healthcare infrastructure, which may hinder timely diagnosis. For example, the implementation and development of comprehensive gastrointestinal endoscopy screening programs might be hindered^28,29^. However, our study found that the decline in the ASMR and ASDR has been most pronounced in high-SDI regions, driven by advances in medical technology, public health education, and effective preventive measures. These trends highlight the complex interactions between socioeconomic development and disease burden and the need for targeted strategies for planning and allocating public health resources.

The implementation of the WHO FCTC began in 2005, and there are significant differences in national and regional tobacco control efforts, so the burden of digestive cancers from smoking varies by region and country^30^. The number of deaths and DALYs from smoking-attributable GI cancers increased from 1990 to 2021 in 21 GBD regions, particularly in East Asia. The ASMR and ASDR for smoking-attributable esophageal and gastric cancers were also highest in East Asia and among the highest for colorectal cancers. As the most populous country in East Asia, China has a high burden of disease. In 2019, China’s smoking population accounted for 30% of the world’s smokers. Chinese smokers consumed one-third of the world’s tobacco – the highest proportions globally. Over the past 30 years, China has experienced a 20.9% decline in smoking prevalence among men and an 18.2% decline among women, but overall tobacco control effectiveness is well below the global average^3,31^. Rapid changes in social status and economic power, and increases in leisure time and socialization have contributed to the use of tobacco. In contrast, reductions in the ASMR and ASDR have been observed in high-income countries in North America, Western Europe, Australasia, and developed countries in Asia, such as Japan and Singapore, reflecting the widespread adoption of better healthcare policies and awareness of disease prevention, as well as the continued strengthening of tobacco control policies. For example, Australia has stricter rules regarding smoking bans^32^, including prohibition in public spaces and underage sales, with penalties for violations.

The EAPCs in the ASMR and ASDR for smoking-attributable GI cancers have decreased in most countries, particularly in Colombia and Singapore. Colombian Anti-Smoking Act (2009 and 2024) limits the places where smoking is allowed; requires the packaging and labels of tobacco products and their derivatives to include clear warnings and pictograms; and increases taxes on traditional tobacco and e-cigarettes^33^. Singapore was one of the first 40 countries to ratify the WHO FCTC, and has gradually raised the legal age for tobacco products from 18 to 21 years^34^. However, there is still an increasing trend in some countries, such as the Democratic Republic of Sao Tome and Principe, the Kingdom of Lesotho, the Republic of Guinea-Bissau, and the Republic of Mali, where there is a significant increase in the EAPCs of the ASMRs and ASDRs for all three GI cancers associated with smoking. Likely contributing factors include economic constraints, limited interventions, and tobacco-use behaviors.

This study revealed that men had significantly more deaths, DALYs, ASMRs, and ASDRs than women from all three GI cancers related to smoking. In 2015, 82.3% of daily smokers worldwide were men, and the age-standardized prevalence of daily smoking was approximately five times higher in men than in women worldwide^35^. The study revealed that the ASMRs and ASDRs for all three types of GI cancers attributable to cigarette smoking declined globally from 1990 to 2021 in both men and women. Over the past three decades, global tobacco control efforts have achieved some progress: the global average smoking prevalence rate for males aged ≥15 years reached 32.7% and 6.6% for females, a decrease of 27.5% and 37.7%, respectively, since 1990^3^. The global burden of the three types of GI cancer attributable to smoking is greater in men than in women, and given the regional differences, there is a need to reduce the high-risk exposure to smoking among women in high-income countries in North America and Western Europe, and among men in East Asia and Eastern Europe. Global disparities in smoking-associated gastrointestinal cancer burden necessitate precisely tailored interventions that account for regional and gender variations. This compelling evidence warrants replacing standardized approaches with context-specific strategies that synthesize epidemiological profiles with sociocultural determinants.

In this study, we assessed cross-country inequalities in three types of gastrointestinal cancers attributable to smoking from 1990 to 2021. The SII revealed a positive association between DALYs and SDI, indicating a disproportionate concentration of disease burden in highly developed countries. All three GI cancers exhibited positive SIIs and CrIs, reflecting a greater burden in high-SDI regions. Urbanization and industrialization in countries with a high SDI may lead to unhealthy lifestyles and higher rates of smoking. In addition, countries with higher SDIs have better healthcare and education, contributing to an aging population. Heterogeneity in absolute and relative inequality changes was observed from 1990 to 2021. The SII for esophageal and gastric cancer patients decreased, but the CrI increased slightly. Although the difference in esophageal and gastric cancer burdens between the highest and lowest socioeconomic groups has decreased, the cancer burden has become more concentrated in the higher socioeconomic groups. In addition, this study revealed that colorectal cancer had the highest relative inequality among the three GI cancers from 1990 to 2021. The dangers of smoking for esophageal and gastric cancers of the upper GI tract are well established, and early gastroscopy is more readily accepted. Poor dietary habits, obesity, and a family history of colorectal cancer are generally recognized as risk factors for colorectal cancer, and not enough attention has been given to the risk of smoking for colorectal cancer. These factors may contribute to disparities in early detection and treatment. In addition, our research indicated that while ASMRs and ASDRs for the three GI cancers associated with smoking exhibited a downward trend in most countries, colorectal cancer exhibited an increasing trend in approximately 28.4% of the countries, with ASDRs demonstrating an upward trend in 25.98% of the countries. This proportion is much greater than that of gastric and esophageal cancer, which warrants greater attention. Therefore, it is imperative to evaluate smoking as an independent risk factor for CRC. Strengthening risk awareness campaigns and expanding public health education on this association could enhance early prevention strategies and mitigate the growing disease burden. Further research should explore the mechanistic pathways linking smoking to CRC to inform targeted interventions.

### Strengths and limitations

The strengths of this study include showing changes in the smoking-attributable disease burden of three common digestive cancers using the most recent epidemiological data on esophageal, gastric, and colorectal cancers from 204 countries and territories from 1990 to 2021 and providing a comprehensive assessment of global inequalities across socioeconomic development.

This study has several limitations that should be considered when interpreting the results. First, the ecological and observational study design prevents us from establishing causal relationships between the analyzed variables. The observed associations merely reflect statistical correlations rather than a direct cause-and-effect link, as we cannot account for all potential confounding factors, such as unmeasured lifestyle variables and regional healthcare disparities, which may influence the outcomes. Second, the data for this study were sourced from the GBD database, which only encompasses population-level information on smoking-related gastrointestinal cancers from 1990 to 2021, and the database exhibits a certain degree of update lag, meaning the most recent epidemiological data may not have been incorporated in a timely manner. Moreover, although the GBD has extensive geographical coverage, significant variations exist across countries and regions in terms of data collection methods, quality, and completeness. In addition, data coverage remains insufficient for certain regions or specific populations, which may affect the comprehensive interpretation of the global distribution patterns of gastrointestinal diseases. Finally, the implementation of tobacco control measures varies considerably across countries, making it difficult to directly link our findings to variations in smoking interventions. Acknowledging these limitations may help guide future research and contribute to a deeper understanding of the mechanisms linking smoking to gastrointestinal cancers.

## CONCLUSIONS

Esophageal, gastric, and colorectal cancer remain major public health challenges worldwide, and although the proportion of the burden of the three types of GI cancer associated with smoking declined between 1990 and 2021, the number of deaths and the absolute number of DALYs continue to increase, highlighting the importance of continued attention. There are significant differences in the distribution and trajectory of the burden of disease by population distribution and socioeconomic level in different regions, particularly in certain parts of East Asia, where the downward trend in the burden of disease is not evident and where strong tobacco control measures are urgently needed. The present study delineates intricate socioeconomic patterns in the burden of esophageal, gastric, and colorectal cancers, emphasizing considerable heterogeneity across regions and income levels.

## Supplementary Material



## Data Availability

The data supporting this research are available from the following sources: http://ghdx.healthdata.org/gbd-results-tool
