# Supplementary material for: Trends and cross-country inequalities in the global burden of gastrointestinal cancers attributable to smoking, 1990–2021: A secondary dataset analysis of GBD Study 2021
Source: Tob Induc Dis. 2026 Jun 12;90:10.18332/tid/217692. doi: 10.18332/tid/217692 (PMC13261636; doi:10.18332/tid/217692)
Supplement: Supplementary file 1 [file TID-24-90-s1.pdf]

Additional Files

Additional File 1: Supplementary figures

Supplementary file Figure S0A: Spatial distribution and ASMR of smoking-related colorectal cancer in 204 countries and territories in 2021. ASMR: age-standardized mortality rates.

Supplementary file Figure S0B: Spatial distribution and ASDR of smoking-related colorectal cancer in 204 countries and territories in 2021. ASDR: age-standardized disability-adjusted life years rate.

Figure S1: The EAPC of ASMR (A, C, E) and ASDR (B, D, F) of three GI cancers attributed to smoking in 2021 of 204 countries and territories. (A, B) esophageal cancer; (C, D) gastric cancer; (E, F) colorectal cancer.

Figure S2: The burden of three GI cancers deaths (A, C, E) and DALYs (B, D, F) attributable to smoking from 1990 to 2021 by sex and SDI region. (A, B) esophageal cancer; (C, D) gastric cancer; (E, F) colorectal cancer. The curves represent ASMR (A, C, E) and ASDR (B, D, F), and the shaded area is 95% of the UI.

Figure S3: The trend in ASMR (A, C, E) and ASDR (B, D, F) of three GI cancers attributable to smoking across 21 GBD regions by SDI from 1990 to 2021. (A, B) esophageal cancer; (C, D) gastric cancer; (E, F) colorectal cancer.

Figure S4: The relationship between smoking attributable three GI cancers in ASMR (A, C, E) or ASDR (B, D, F) by 204 countries and SDI in 2021. (A, B) esophageal cancer; (C, D) gastric cancer; (E, F) colorectal cancer.

Figure S5: Analysis of absolute inequality (A, C, E) and relative inequality (B, D, F) in three GI cancers attributable to smoking between 1990 and 2021. (A, B) esophageal cancer; (C, D) gastric cancer; (E, F) colorectal cancer.

Additional File 2: Supplementary tables

Table S1: Global burden of esophageal cancer attributable to smoking in 1990 and 2021, and the temporal trends from 1990 to 2021.

Table S2: Global burden of gastric cancer attributable to smoking in 1990 and 2021, and the temporal trends from 1990 to 2021.

Table S3: Global burden of colorectal cancer attributable to smoking in 1990 and 2021, and the temporal trends from 1990 to 2021.

Table S4: the ASMR and ASDR of three GI cancers attributed to smoking in 2021 of 204 countries and territories.

Table S5: the EAPC of ASMR and ASDR of three GI cancers attributed to smoking in 2021 of 204 countries and territories.

Table S6: The DALYs of colorectal cancer attributable to smoking in males by SDI region, from1990 to 2021.

Table S7: The DALYs of colorectal cancer attributable to smoking in females by SDI region, from1990 to 2021.

Table S8: The DALYs of esophageal cancer attributable to smoking in males by SDI region, from1990 to 2021.

Table S10: The DALYs of gastric cancer attributable to smoking in males by SDI region, from1990 to 2021.

Table S11: The DALYs of gastric cancer attributable to smoking in females by SDI region, from1990 to 2021.

Table S12: The deaths of colorectal cancer attributable to smoking in males by SDI region, from1990 to 2021.

Table S13: The deaths of colorectal cancer attributable to smoking in females by SDI region, from1990 to 2021.

Table S14: The deaths of esophageal cancer attributable to smoking in males by SDI region, from1990 to 2021.

Table S15: The deaths of esophageal cancer attributable to smoking in females by SDI region, from1990 to 2021.

Table S16: The deaths of gastric cancer attributable to smoking in males by SDI region, from1990 to 2021.

Table S17: The deaths of gastric cancer attributable to smoking in females by SDI region, from1990 to 2021.

Figure S0A: Spatial distribution and ASMR of smoking-related colorectal cancer in 204 countries and territories in 2021. ASMR: age-standardized mortality rates.

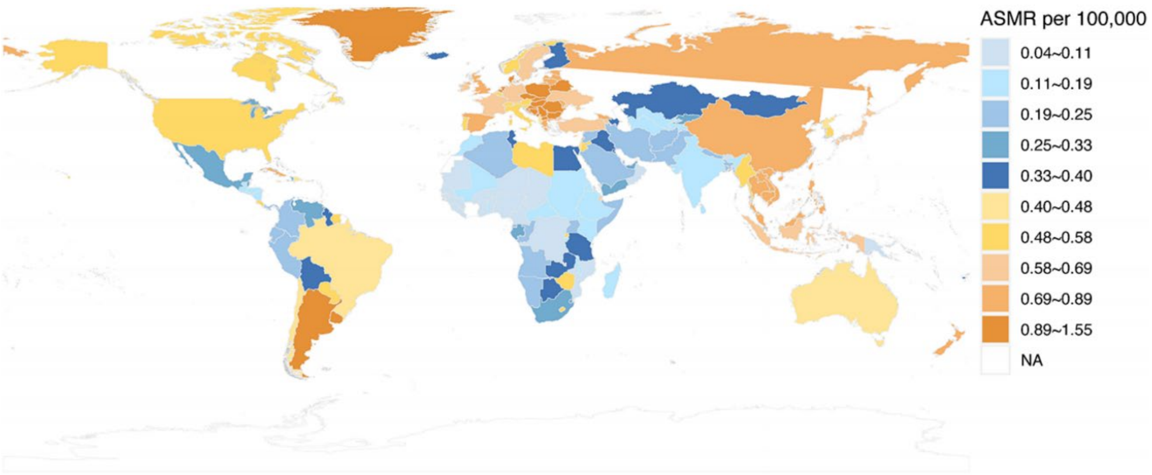

Figure S0B: Spatial distribution and ASDR of smoking-related colorectal cancer in 204 countries and territories in 2021. ASDR: age-standardized disability-adjusted life years rate.

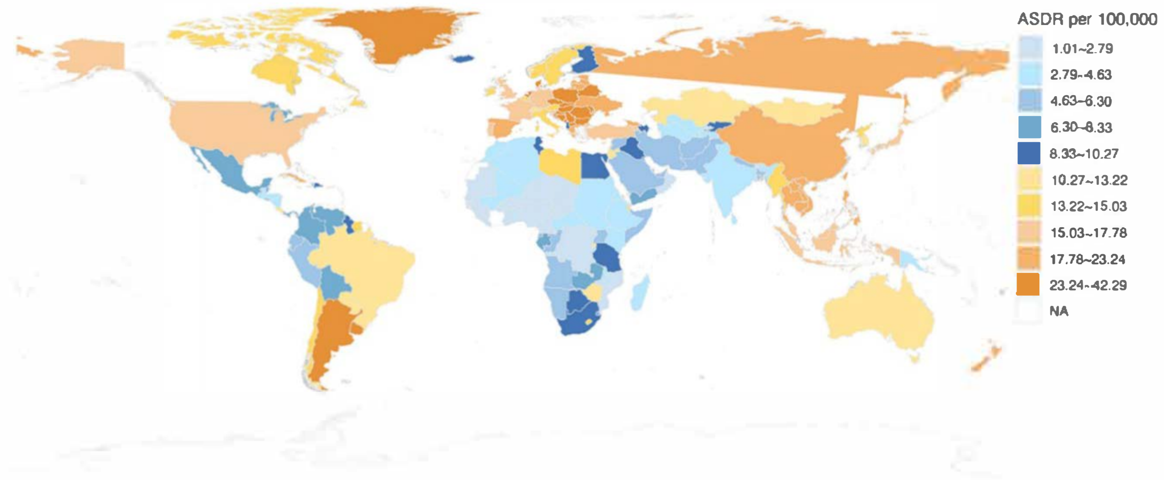

Figure S1: The EAPC of ASMR (A, C, E) and ASDR (B, D, F) of three GI cancers attributed to smoking in 2021 of 204 countries and territories. (A, B) esophageal cancer; (C , D) gastric cancer; (E, F) colorectal cancer.

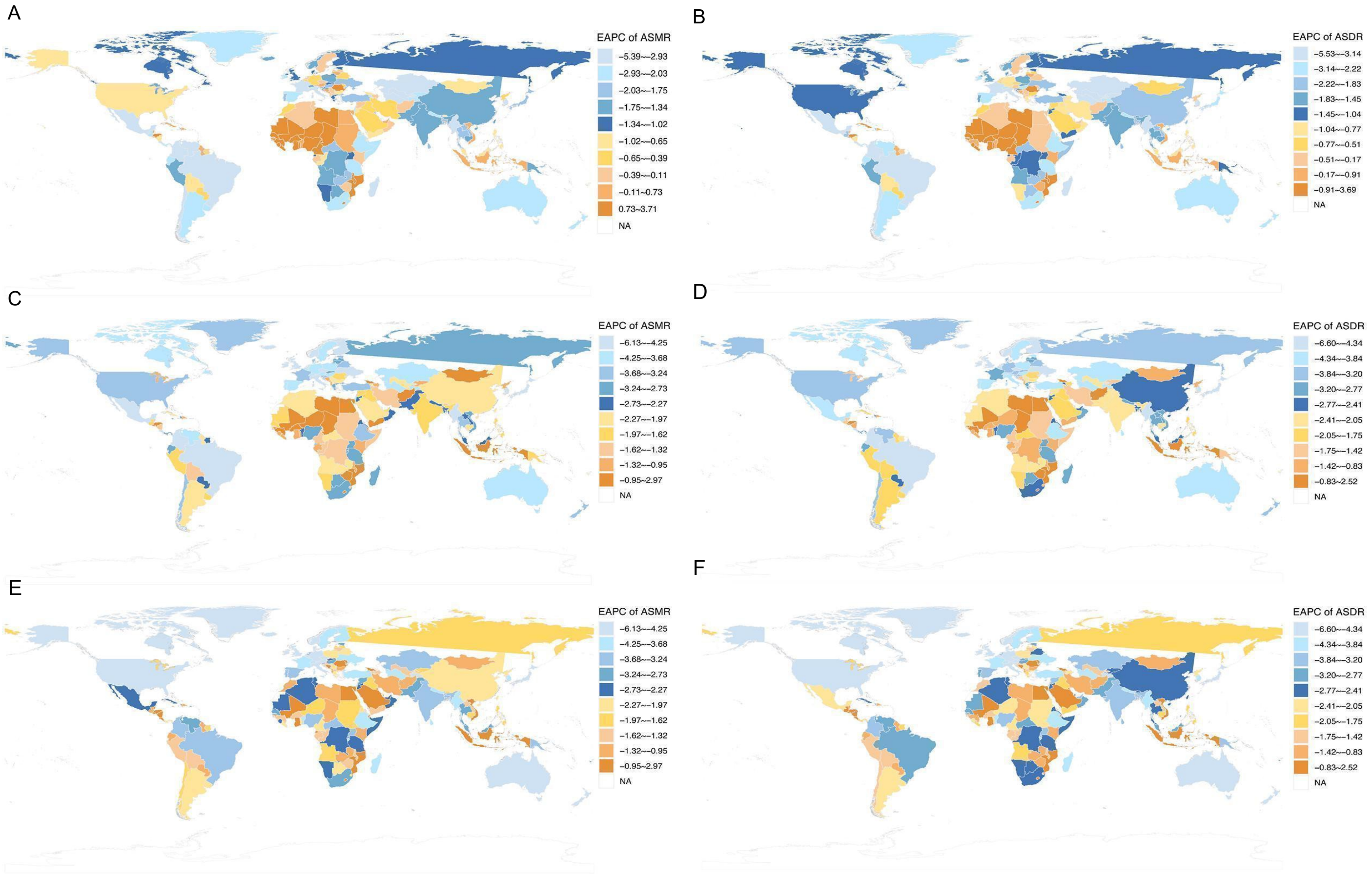

GI: gastrointestinal; EAPC: estimated annual percentage change; ASMR: age-standardized mortality rates; ASDR: age-standardized disability-adjusted life years rate.

Figure S2: The burden of three GI cancers deaths (A, C, E) and DALYs (B, D, F) attributable to smoking from 1990 to 2021 by sex and SDI region. (A, B) esophageal cancer; (C, D) gastric cancer; (E, F) colorectal cancer. The curves represent ASMR (A, C, E) and ASDR (B, D, F), and the shaded area is 95% of the UI.

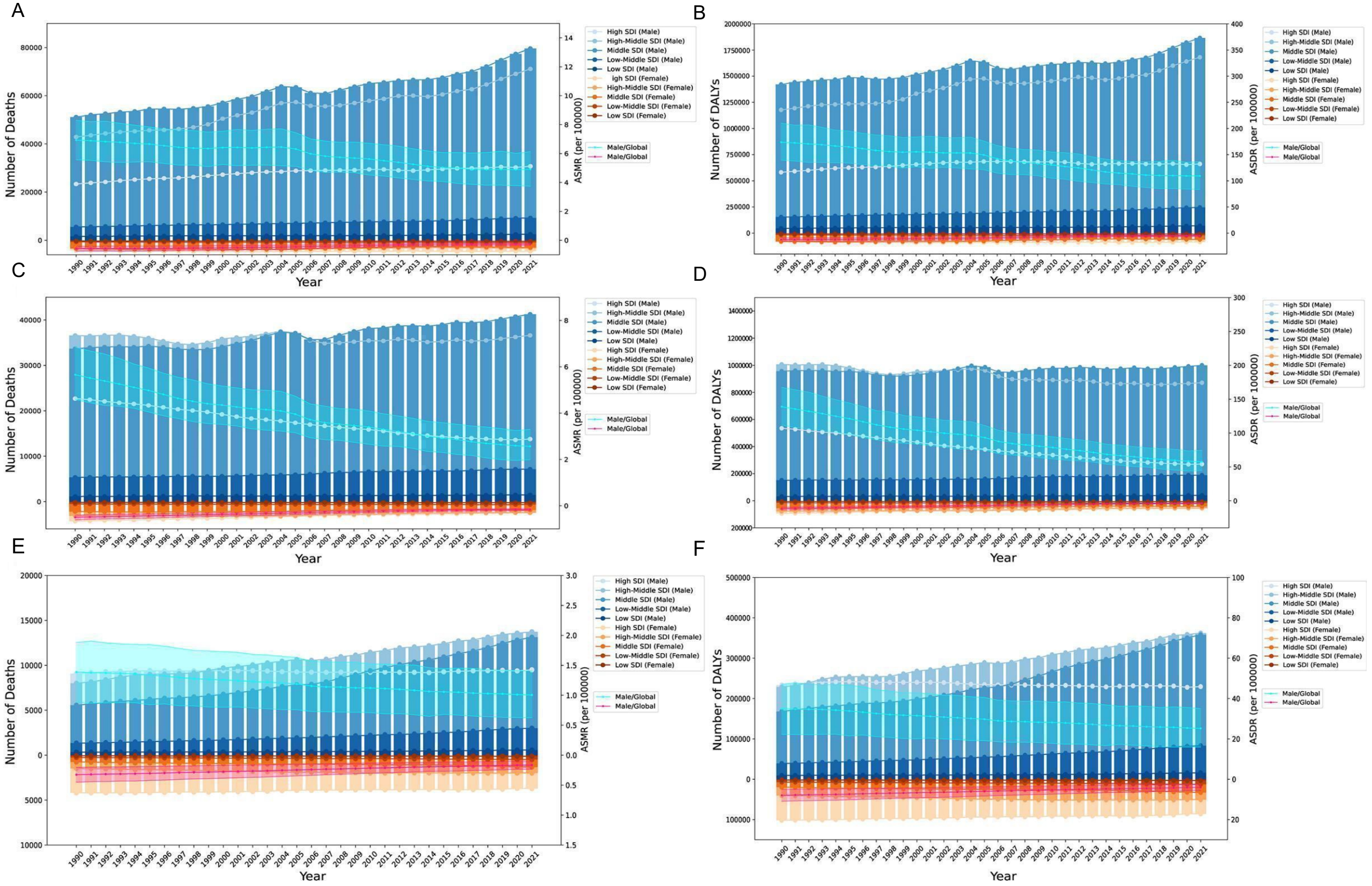

GI: gastrointestinal; UI: uncertainty interval; SDI: Socio-demographic Index; ASMR: age-standardized mortality rate; ASDR: age-standardized disability-adjusted life years rate.

Figure S3: The trend in ASMR (A, C, E) and ASDR (B, D, F) of three GI cancers attributable to smoking across 21 GBD regions by SDI from 1990 to 2021. (A, B) esophageal cancer; (C, D) gastric cancer; (E, F) colorectal cancer.

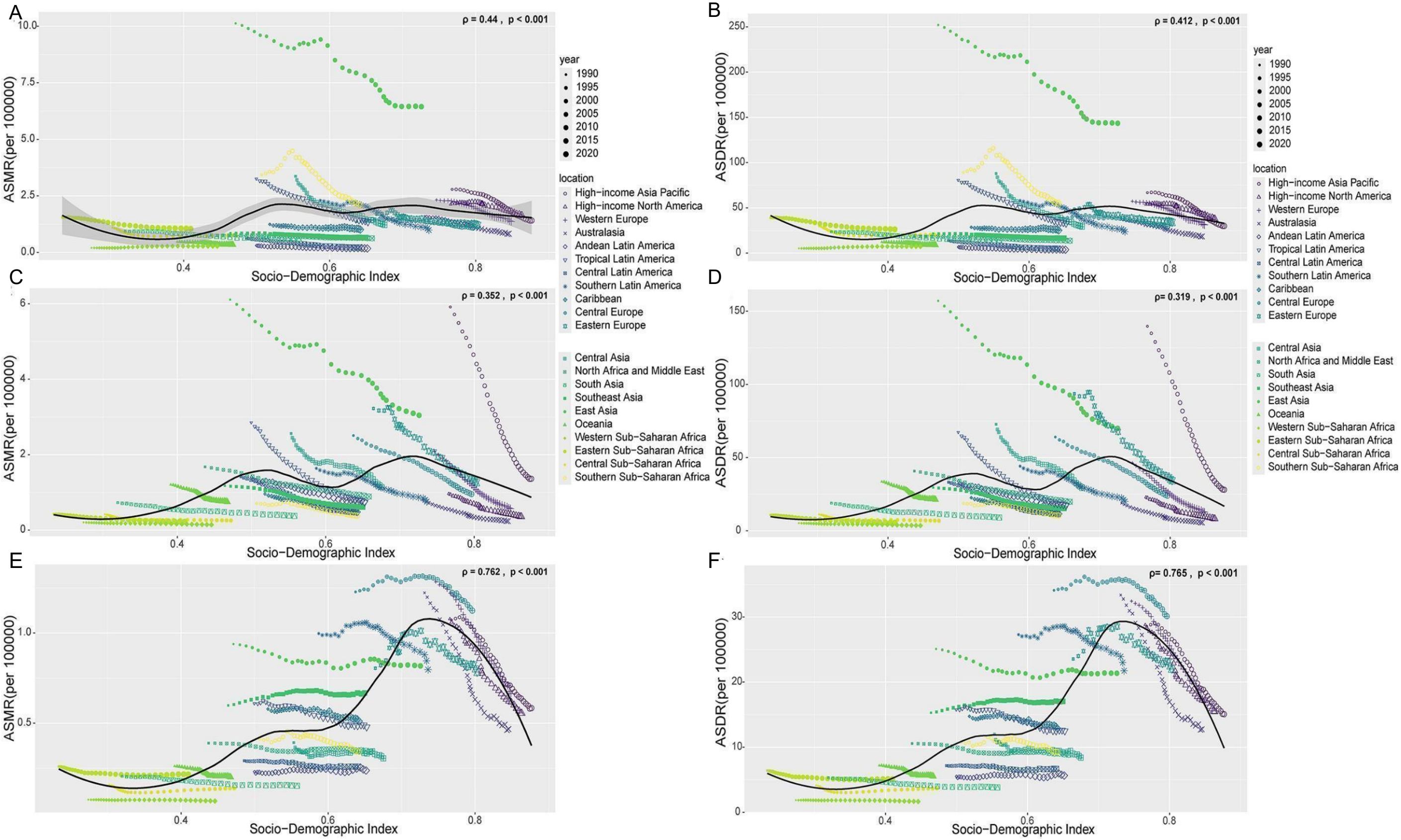

GI: gastrointestinal; ASMR: age-standardized mortality rates; ASDR: age-standardized disability-adjusted life years rate.

Figure S4: The relationship between smoking attributable three GI cancers in ASMR (A, C, E) or ASDR (B, D, F) by 204 countries and SDI in 2021. (A, B) esophageal cancer; (C, D) gastric cancer; (E, F) colorectal cancer.

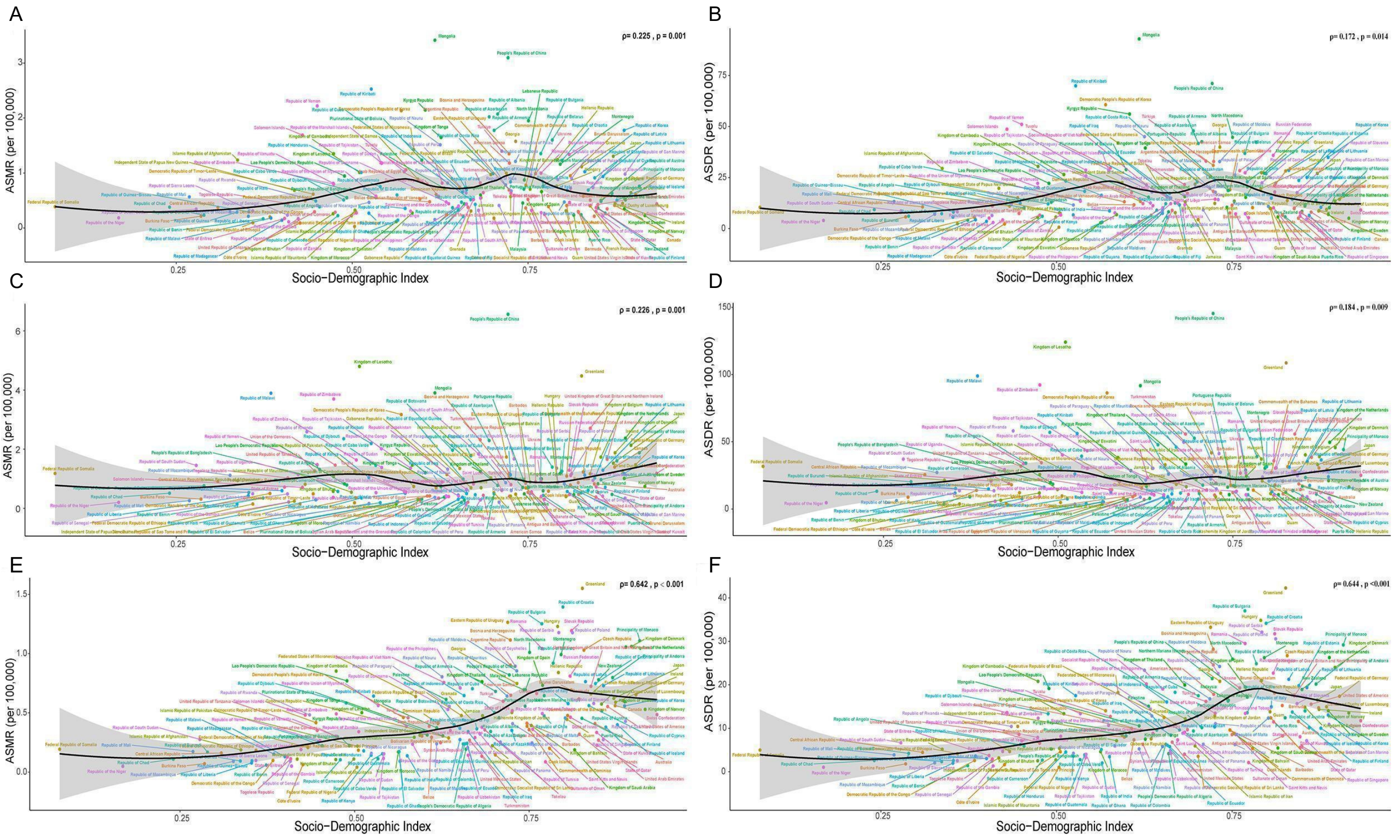

GI: gastrointestinal; ASMR: age-standardized mortality rates; ASDR: age-standardized disability-adjusted life years rate.

Figure S5: Analysis of absolute inequality (A, C, E) and relative inequality (B, D, F) in three GI cancers attributable to smoking between 1990 and 2021. (A, B) esophageal cancer; (C, D) gastric cancer; (E, F) colorectal cancer.

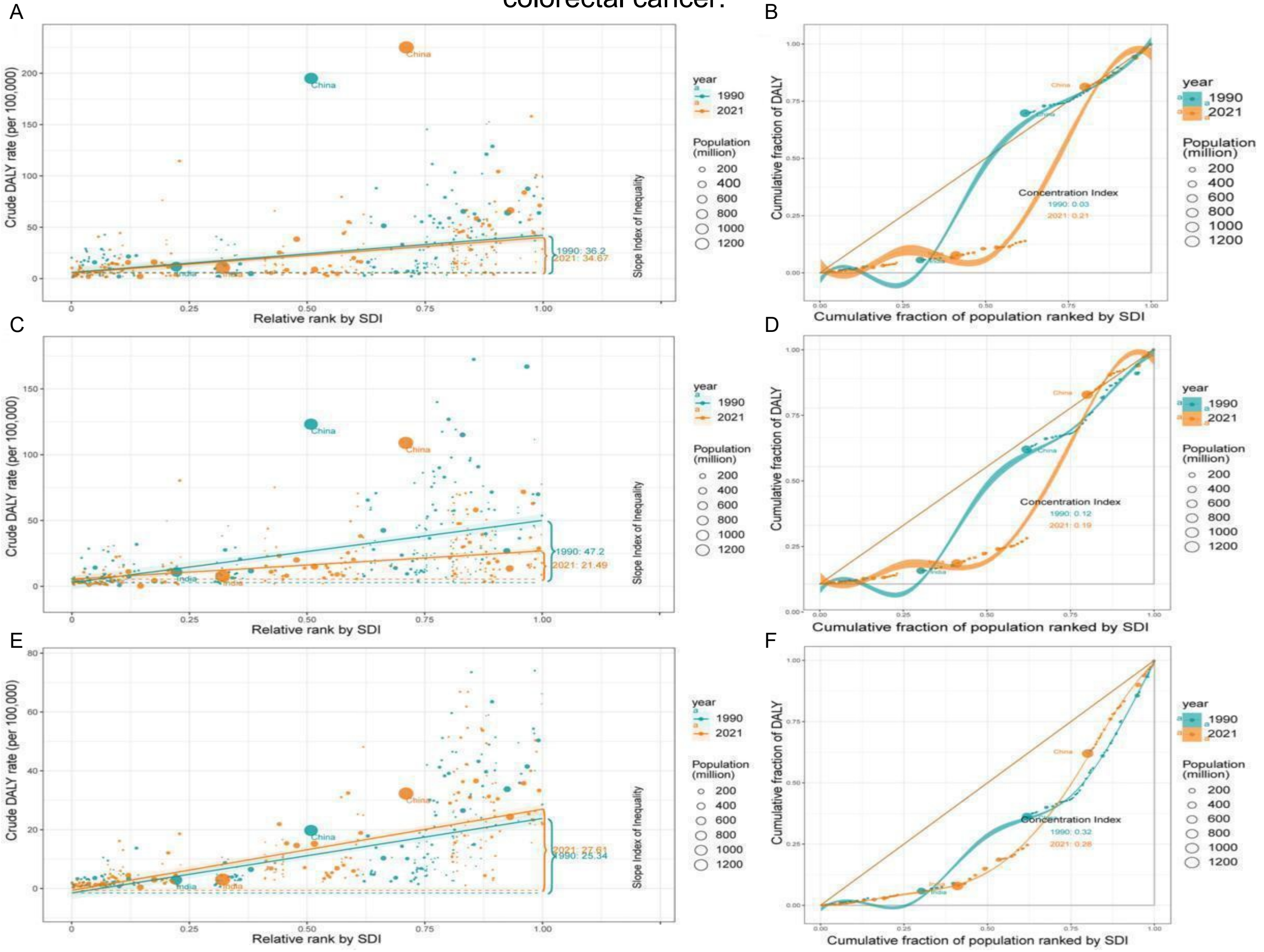

DALY: disability-adjusted life years rate; SDI: Socio-demographic Index.

Additional File2: Supplementary tables

Table S1: Global burden of esophageal cancer attributable to smoking in 1990 and 2021, and the temporal trends from 1990 to 2021.

Location	Deaths				EAPC of ASMR from 1990-2019	DALYs				EAPC of ASDR from 1990-2021
	Number (95% UI)		ASMR per 100000 (95% UI)			Number (95% UI)		ASDR per 100000 (95% UI)		
	1990	2021	1990	2021		1990	2021	1990	2021	
Low SDI	1885 (1437, 2333)	2892 (2197, 3617)	0.87 (0.66, 1.08)	0.59 (0.45, 0.74)	-1.47 (-1.57, -1.37)	52655 (40274, 65298)	80075 (60459, 101003)	21.82 (16.63, 27.06)	14.79 (11.21, 18.59)	-1.53 (-1.63, -1.42)
Low-middle SDI	6223 (4934, 7593)	10174 (7978, 12436)	1.06 (0.84, 1.30)	0.73 (0.57, 0.89)	-1.33 (-1.41, -1.26)	171318 (135856, 208390)	267580 (209089, 327031)	26.54 (21.08, 32.26)	17.83 (13.95, 21.83)	-1.41 (-1.48, -1.34)
Middle SDI	54586 (43259, 68159)	82572 (60685, 108092)	5.36 (4.24, 6.69)	3.14 (2.31, 4.11)	-1.91 (-2.03, -1.8)0	1500657 (1185142, 1878363)	1926460 (1423912, 2515937)	135.81 (107.56, 169.80)	69.51 (51.30, 90.83)	-2.37 (-2.48, -2.26)
High-middle SDI	46134 (36654, 56475)	74471 (55783, 97501)	4.61 (3.67, 5.64)	3.70 (2.77, 4.83)	-0.85 (-0.99, -0.71)	1247742 (987239, 1526747)	1742872 (1305980, 2286442)	121.09 (96.00, 148.22)	86.54 (64.83, 113.38)	-1.24 (-1.38, -1.11)
High SDI	27562 (22426, 32120)	35278 (27498, 42904)	2.49 (2.03, 2.90)	1.65 (1.29, 2.00)	-1.48 (-1.62, -1.34)	667002 (547352, 777432)	746185 (590486, 902021)	61.87 (50.84, 72.11)	37.57 (29.85, 45.36)	-1.74 (-1.89, -1.59)
High-income Asia Pacific	5640 (4664, 6520)	7005 (5405, 8510)	2.79 (2.31, 3.23)	1.41 (1.10, 1.71)	-2.45 (-2.60, -2.30)	138884 (115202, 159724)	130924 (102462, 158878)	66.78 (55.38, 76.83)	29.89 (23.77, 36.13)	-2.88 (-3.07, -2.69)

Central Asia	1582 (1258, 1920)	960 (741, 1192)	3.37 (2.66, 4.10)	1.21 (0.93, 1.51)	-3.17 (-3.27, -3.06)	43210 (34561, 51906)	25119 (19572, 31248)	87.87 (69.94, 106.09)	29.10 (22.61, 36.10)	-3.50 (-3.59, -3.41)
East Asia	85524 (66368, 107038)	141688 (104484, 185047)	10.13 (7.87, 12.63)	6.44 (4.76, 8.40)	-1.61 (-1.77, -1.45)	2333080 (1804709, 2931961)	3270718 (2400251, 4302891)	252.13 (196.07, 316.03)	143.42 (105.65, 188.21)	-2.01 (-2.15, -1.86)
South Asia	5169 (4025, 6440)	8511 (6599, 10654)	0.94 (0.73, 1.18)	0.60 (0.46, 0.75)	-1.71 (-1.82, -1.61)	144226 (113131, 178616)	220226 (169862, 274153)	23.48 (18.31, 29.21)	14.38 (11.10, 17.91)	-1.83 (-1.93, -1.73)
Southeast Asia	2110 (1610, 2742)	4283 (3358, 5338)	0.86 (0.65, 1.10)	0.66 (0.52, 0.82)	-1.01 (-1.07, -0.96)	58159 (44280, 76288)	115681 (90160, 143817)	21.50 (16.36, 28.02)	16.34 (12.77, 20.30)	-1.01 (-1.05, -0.96)
Australasia	382 (291, 477)	457 (320, 607)	1.60 (1.22, 2.00)	0.83 (0.58, 1.09)	-2.25 (-2.33, -2.16)	8760 (6779, 10800)	9391 (6771, 12237)	37.17 (28.81, 45.80)	18.34 (13.42, 23.76)	-2.35 (-2.43, -2.28)
Caribbean	311 (241, 390)	549 (415, 706)	1.24 (0.96, 1.56)	1.01 (0.76, 1.30)	-0.43 (-0.52, -0.34)	7406 (5827, 9146)	13678 (10370, 17395)	28.54 (22.43, 35.29)	25.10 (19.06, 31.91)	-0.16 (-0.26, -0.07)
Central Europe	2064 (1689, 2428)	2347 (1840, 2860)	1.36 (1.11, 1.61)	1.09 (0.86, 1.33)	-0.90 (-1.06, -0.74)	57284 (47400, 66699)	59234 (47009, 71587)	37.64 (31.13, 43.89)	29.19 (23.32, 35.17)	-1.03 (-1.21, -0.84)
Eastern Europe	4869 (4036, 5628)	4237 (3414, 5053)	1.69 (1.40, 1.95)	1.20 (0.97, 1.43)	-1.32 (-1.45, -1.20)	140202 (116783, 161737)	115500 (92858, 137805)	48.48 (40.46, 55.94)	33.70 (27.11, 40.20)	-1.43 (-1.57, -1.29)
Western Europe	13323 (10765, 15591)	12965 (9923, 16182)	2.32 (1.87, 2.71)	1.37 (1.06, 1.69)	-1.76 (-1.85, -1.67)	313021 (255343, 363237)	265965 (208553, 327629)	57.24 (46.73, 66.47)	31.18 (24.47, 38.14)	-2.01 (-2.11, -1.90)

Andean Latin America	58 (43, 74)	100 (71, 136)	0.30 (0.22, 0.39)	0.17 (0.12, 0.24)	-1.76 (-1.87, -1.64)	1357 (1008, 1742)	2180 (1533, 3001)	6.70 (4.98, 8.62)	3.70 (2.60, 5.09)	-1.89 (-2.00, -1.78)
Central Latin America	473 (368, 580)	535 (400, 669)	0.62 (0.48, 0.77)	0.22 (0.16, 0.27)	-3.60 (-3.71, -3.49)	11365 (9040, 13809)	12412 (9327, 15541)	13.70 (10.80, 16.71)	4.90 (3.68, 6.13)	-3.53 (-3.64, -3.42)
Southern Latin America	1067 (825, 1309)	868 (641, 1096)	2.30 (1.77, 2.83)	0.99 (0.73, 1.24)	-2.72 (-2.99, -2.45)	27244 (21386, 32955)	20256 (15396, 25376)	57.98 (45.55, 70.24)	23.65 (18.08, 29.54)	-2.92 (-3.19, -2.66)
Tropical Latin America	2870 (2283, 3441)	3477 (2616, 4421)	3.25 (2.58, 3.93)	1.35 (1.01, 1.72)	-2.99 (-3.09, -2.88)	77050 (62062, 91835)	86305 (64686, 108805)	80.58 (64.62, 96.45)	32.78 (24.58, 41.39)	-3.11 (-3.24, -2.98)
North Africa and Middle East	1195 (930, 1471)	2560 (1956, 3205)	0.75 (0.58, 0.93)	0.62 (0.47, 0.78)	-0.68 (-0.73, -0.64)	32015 (25014, 39333)	63038 (48216, 78831)	18.14 (14.13, 22.23)	13.63 (10.40, 17.04)	-1.04 (-1.09, -0.99)
High-income North America	7367 (5896, 8712)	11026 (8347, 13831)	2.12 (1.70, 2.50)	1.63 (1.24, 2.04)	-0.96 (-1.13, -0.78)	178215 (144111, 208865)	242511 (188030, 298973)	53.71 (43.50, 62.86)	37.59 (29.22, 46.29)	-1.25 (-1.43, -1.08)
Oceania	14 (9, 19)	25 (19, 34)	0.47 (0.32, 0.66)	0.34 (0.25, 0.46)	-1.18 (-1.23, -1.12)	400 (266, 565)	755 (546, 1038)	12.17 (8.28, 17.08)	8.85 (6.45, 12.03)	-1.15 (-1.20, -1.09)
Central Sub-Saharan Africa	253 (177, 349)	425 (292, 593)	1.09 (0.77, 1.50)	0.75 (0.52, 1.04)	-1.30 (-1.58, -1.02)	7551 (5256, 10363)	12831 (8766, 17838)	29.50 (20.65, 40.67)	20.08 (13.81, 28.04)	-1.34 (-1.61, -1.07)

Eastern Sub-Saharan Africa	1105 (829, 1398)	1693 (1276, 2181)	1.55 (1.16, 1.96)	1.04 (0.79, 1.33)	-1.57 (-1.69, -1.45)	30928 (23007, 39299)	47810 (35738, 62053)	38.91 (29.15, 49.45)	26.19 (19.66, 33.83)	-1.58 (-1.71, -1.45)
Southern Sub-Saharan Africa	904 (694, 1120)	1199 (919, 1503)	3.43 (2.61, 4.27)	2.06 (1.58, 2.57)	-2.23 (-2.68, -1.78)	25702 (19964, 31935)	34444 (26598, 42996)	89.20 (69.07, 110.78)	54.82 (42.13, 68.25)	-2.16 (-2.59, -1.71)
Western Sub-Saharan Africa	173 (127, 233)	552 (397, 730)	0.20 (0.15, 0.27)	0.28 (0.20, 0.37)	1.52 (1.34, 1.71)	4893 (3595, 6616)	16053 (11435, 21052)	5.21 (3.82, 7.02)	7.28 (5.23, 9.6)0	1.51 (1.33, 1.69)

DALYs: disability-adjusted life years; ASMR: age-standardized mortality rates; ASDR: age-standardized DALYs rates; EAPC: estimated annual percentage change.

Table S2: Global burden of gastric cancer attributable to smoking in 1990 and 2021, and the temporal trends from 1990 to 2021.

**Location**	**Deaths**				**EAPC of ASMR from 1990-2019**	**DALYs**				**EAPC of ASDR from 1990-[illegible]**
	Number (95% UI)		ASMR per 100000 (95% UI)			Number (95% UI)		ASDR per 100000 (95% UI)		
	[illegible]	[illegible]	[illegible]	[illegible]		[illegible]	[illegible]	[illegible]	[illegible]	
Low SDI	1287 (916, 1623)	1710 (1127, 2129)	0.59 (0.42, 0.74)	0.36 (0.23, 0.44)	-1.60 (-1.67, -1.53)	36731 (26058, 46343)	46646 (31155, 58286)	14.99 (10.67, 18.91)	8.65 (5.72, 10.79)	-1.85 (-1.92, -1.78)
Low-middle SDI	5875 (4621, 7463)	7784 (5978, 9586)	0.99 (0.78, 1.26)	0.56 (0.43, 0.69)	-1.77 (-1.83, -1.71)	166305 (130651, 210207)	205166 (157311, 253378)	25.32 (19.87, 32.04)	13.64 (10.45, 16.82)	-1.92 (-1.97, -1.87)
Middle SDI	35854 (27881, 45182)	43073 (32395, 57603)	3.50 (2.72, 4.45)	1.63 (1.23, 2.18)	-2.45 (-2.52, -2.37)	1010299 (774014, 1270169)	1041308 (777836, 1396036)	90.29 (69.96, 113.53)	37.48 (28.13, 50.23)	-2.84 (-2.9, -2.77)
High-middle SDI	39723 (32105, 47502)	39047 (30050, 51057)	3.98 (3.22, 4.75)	1.95 (1.50, 2.55)	-2.32 (-2.40, -2.24)	1084808 (874067, 1299890)	925650 (712993, 1220612)	105.19 (84.78, 126.10)	46.47 (35.85, 61.16)	-2.69 (-2.77, -2.61)
High SDI	26991 (22570, 31941)	16255 (13205, 19907)	2.42 (2.02, 2.86)	0.73 (0.60, 0.89)	-3.99 (-4.04, -3.93)	629078 (530058, 735218)	317863 (260975, 381945)	57.90 (48.80, 67.70)	15.84 (12.99, 18.91)	-4.30 (-4.35, -4.25)
High-income Asia Pacific	11774 (9706, 14249)	7130 (5654, 9036)	5.92 (4.88, 7.17)	1.36 (1.10, 1.68)	-4.98 (-5.06, -4.89)	287266 (235854, 343553)	124539 (100751, 153825)	139.68 (115.13, 167.03)	28.05 (23.14, 33.92)	-5.38 (-5.47, -5.30)
Central Asia	1231 (1024, 1478)	933 (752, 1128)	2.56 (2.12, 3.09)	1.14 (0.92, 1.38)	-2.13 (-2.30, -1.97)	36381 (30565, 43166)	26008 (21089, 31451)	72.78 (61.03, 86.72)	29.30 (23.74, 35.43)	-2.56 (-2.70, -2.43)

East Asia	52449 (38960, 66990)	66779 (49132, 92419)	6.11 (4.58, 7.85)	3.04 (2.24, 4.21)	-2.17 (-2.29, -2.05)	1475263 (1091203, 1885797)	1580210 (1162456, 2205354)	157.07 (116.54, 200.35)	70.00 (51.49, 97.50)	-2.57 (-2.67, -2.48)
South Asia	4119 (3146, 5417)	5370 (4033, 6893)	0.73 (0.56, 0.96)	0.38 (0.28, 0.48)	-2.04 (-2.13, -1.95)	118993 (90634, 156081)	138736 (105641, 179492)	18.91 (14.44, 24.88)	9.04 (6.86, 11.69)	-2.29 (-2.38, -2.21)
Southeast Asia	2897 (2141, 3628)	3962 (3093, 5118)	1.18 (0.87, 1.48)	0.61 (0.48, 0.79)	-2.38 (-2.48, -2.29)	80944 (59428, 101197)	107993 (83679, 138316)	29.63 (21.83, 37.04)	15.31 (11.90, 19.75)	-2.37 (-2.45, -2.29)
Australasia	190 (152, 231)	129 (100, 166)	0.80 (0.64, 0.97)	0.24 (0.19, 0.30)	-3.91 (-4.03, -3.78)	4426 (3586, 5262)	2754 (2199, 3422)	18.89 (15.34, 22.42)	5.66 (4.55, 6.96)	-3.88 (-4.01, -3.76)
Caribbean	242 (196, 295)	260 (199, 322)	0.96 (0.77, 1.17)	0.48 (0.37, 0.60)	-2.23 (-2.31, -2.15)	5919 (4816, 7124)	6247 (4800, 7656)	22.60 (18.36, 27.24)	11.57 (8.89, 14.19)	-2.19 (-2.27, -2.11)
Central Europe	3778 (3190, 4426)	2110 (1701, 2539)	2.51 (2.11, 2.95)	0.95 (0.77, 1.15)	-3.11 (-3.23, -2.99)	97480 (82511, 114192)	49526 (40280, 59385)	64.06 (54.29, 74.99)	23.94 (19.58, 28.66)	-3.16 (-3.27, -3.04)
Eastern Europe	9199 (7745, 10702)	4418 (3628, 5314)	3.22 (2.71, 3.76)	1.26 (1.03, 1.50)	-3.29 (-3.49, -3.10)	265795 (224633, 306469)	115871 (95233, 138251)	93.58 (79.18, 108.12)	34.15 (28.07, 40.61)	-3.55 (-3.74, -3.36)
Western Europe	12415 (10171, 14686)	5545 (4382, 6788)	2.08 (1.71, 2.46)	0.57 (0.46, 0.69)	-4.15 (-4.20, -4.10)	262440 (216926, 307176)	108740 (87991, 130445)	46.42 (38.48, 54.26)	12.75 (10.43, 15.26)	-4.09 (-4.13, -4.05)
Andean Latin America	277 (223, 346)	439 (332, 581)	1.40 (1.12, 1.75)	0.75 (0.57, 1.00)	-2.12 (-2.26, -1.98)	7132 (5715, 8836)	10674 (7937, 14162)	33.95 (27.27, 42.34)	17.80 (13.24, 23.65)	-2.20 (-2.33, -2.06)

Central Latin America	1107 (909, 1308)	1160 (916, 1439)	1.41 (1.15, 1.68)	0.47 (0.37, 0.58)	-3.95 (-4.09, -3.80)	28564 (23674, 33643)	29382 (23108, 36220)	33.14 (27.39, 39.15)	11.44 (9.02, 14.13)	-3.81 (-3.95, -3.67)
Southern Latin America	764 (615, 918)	651 (521, 797)	1.64 (1.32, 1.98)	0.75 (0.61, 0.92)	-2.37 (-2.53, -2.20)	20744 (16856, 24556)	16801 (13645, 20302)	44.13 (35.89, 52.26)	20.04 (16.33, 24.23)	-2.42 (-2.59, -2.26)
Tropical Latin America	2465 (1989, 2983)	1890 (1502, 2351)	2.85 (2.29, 3.49)	0.74 (0.59, 0.92)	-4.59 (-4.71, -4.48)	63812 (52072, 75933)	45162 (36443, 55400)	67.35 (54.74, 80.78)	17.29 (13.93, 21.21)	-4.69 (-4.81, -4.56)
North Africa and Middle East	2674 (1855, 3426)	3703 (2333, 4614)	1.68 (1.17, 2.15)	0.89 (0.56, 1.11)	-2.01 (-2.09, -1.94)	72912 (50364, 93115)	93116 (59062, 116916)	40.82 (28.28, 52.38)	19.84 (12.53, 24.81)	-2.33 (-2.41, -2.25)
High-income North America	3432 (2774, 4132)	2380 (1846, 2970)	0.96 (0.78, 1.16)	0.36 (0.28, 0.44)	-3.31 (-3.36, -3.26)	78133 (63544, 93566)	51015 (40682, 62404)	22.94 (18.66, 27.42)	8.16 (6.58, 9.94)	-3.40 (-3.45, -3.35)
Oceania	37 (25, 50)	60 (43, 84)	1.21 (0.84, 1.62)	0.75 (0.54, 1.02)	-1.66 (-1.76, -1.57)	1179 (776, 1628)	1976 (1375, 2757)	33.57 (22.79, 45.78)	21.35 (15.00, 29.60)	-1.58 (-1.69, -1.48)
Central Sub-Saharan Africa	96 (62, 127)	145 (95, 197)	0.42 (0.27, 0.55)	0.26 (0.17, 0.34)	-1.56 (-1.75, -1.36)	2874 (1864, 3833)	4479 (2918, 6072)	11.22 (7.24, 14.84)	6.88 (4.51, 9.34)	-1.54 (-1.72, -1.36)
Eastern Sub-Saharan Africa	305 (215, 391)	363 (269, 457)	0.42 (0.30, 0.54)	0.22 (0.17, 0.28)	-2.31 (-2.39, -2.22)	8665 (6032, 11066)	10380 (7635, 13169)	10.76 (7.58, 13.73)	5.59 (4.12, 7.04)	-2.34 (-2.42, -2.25)

Southern Sub-Saharan Africa	190 (137, 235)	226 (175, 278)	0.71 (0.51, 0.89)	0.38 (0.29, 0.47)	-2.19 (-2.45, -1.93)	5560 (4013, 6903)	6702 (5193, 8214)	18.83 (13.53, 23.40)	10.46 (8.07, 12.85)	-2.05 (-2.29, -1.81)
Western Sub-Saharan Africa	176 (133, 226)	272 (191, 348)	0.20 (0.16, 0.26)	0.14 (0.10, 0.18)	-1.01 (-1.08, -0.95)	4955 (3713, 6284)	7687 (5400, 9821)	5.26 (3.96, 6.71)	3.54 (2.50, 4.53)	-1.13 (-1.20, -1.06)

DALYs: disability-adjusted life years; ASMR: age-standardized mortality rates; ASDR: age-standardized DALYs rates; EAPC: estimated annual percentage change.

Table S3: Global burden of colorectal cancer attributable to smoking in 1990 and 2021, and the temporal trends from 1990 to 2021.

Location	Deaths				EAPC of ASMR from 1990-2019	DALYs				EAPC of ASDR from 1990-2021
	Number (95% UI)		ASMR per 100000 (95% UI)			Number (95% UI)		ASDR per 100000 (95% UI)		
	1990	2021	1990	2021		1990	2021	1990	2021	
Low SDI	400 (246, 575)	692 (421, 980)	0.18 (0.11, 0.26)	0.15 (0.09, 0.21)	-0.84 (-0.92, -0.76)	11302 (7054, 16190)	19096 (11714, 26945)	4.66 (2.89, 6.68)	3.54 (2.16, 5.00)	-1.01 (-1.09, -0.93)
Low-middle SDI	1595 (997, 2228)	3482 (2162, 4923)	0.27 (0.16, 0.37)	0.25 (0.15, 0.35)	-0.28 (-0.32, -0.24)	46066 (28785, 64202)	95591 (59545, 135417)	6.95 (4.35, 9.70)	6.28 (3.91, 8.88)	-0.35 (-0.38, -0.32)
Middle SDI	6316 (4021, 8602)	14441 (8977, 20447)	0.62 (0.39, 0.84)	0.54 (0.33, 0.76)	-0.45 (-0.50, -0.39)	187638 (119051, 255845)	390958 (244023, 549549)	16.43 (10.44, 22.40)	13.88 (8.65, 19.50)	-0.54 (-0.61, -0.48)
High-middle SDI	9480 (6009, 12803)	15719 (9899, 21835)	0.95 (0.60, 1.28)	0.79 (0.50, 1.10)	-0.62 (-0.69, -0.56)	268894 (171305, 362492)	413021 (259115, 568504)	26.03 (16.55, 35.10)	21.02 (13.17, 28.94)	-0.74 (-0.78, -0.69)
High SDI	13255 (8278, 18169)	13220 (8023, 18488)	1.2 (0.75, 1.64)	0.64 (0.39, 0.89)	-2.15 (-2.20, -2.11)	335149 (214286, 458439)	315506 (195787, 437063)	31.21 (20.00, 42.65)	16.85 (10.56, 23.23)	-2.09 (-2.13, -2.05)
High-income Asia Pacific	2162 (1373, 2897)	2670 (1644, 3732)	1.07 (0.68, 1.44)	0.58 (0.36, 0.80)	-2.22 (-2.30, -2.15)	59304 (38431, 79342)	58423 (36455, 80791)	28.57 (18.50, 38.24)	15.08 (9.53, 20.81)	-2.31 (-2.40, -2.23)
Central Asia	192 (123, 260)	256 (162, 354)	0.39 (0.25, 0.53)	0.3 (0.19, 0.42)	-0.25 (-0.40, -0.09)	6118 (3934, 8280)	7609 (4799, 10489)	12.02 (7.72, 16.29)	8.39 (5.29, 11.59)	-0.69 (-0.83, -0.56)

East Asia	8002 (5104, 11146)	17816 (10959, 26289)	0.94 (0.59, 1.30)	0.82 (0.50, 1.20)	-0.36 (-0.46, -0.25)	239143 (153959, 333987)	473620 (287331, 702516)	25.10 (16.14, 34.93)	21.37 (13.01, 31.65)	-0.45 (-0.59, -0.31)
South Asia	1154 (713, 1649)	2214 (1375, 3151)	0.21 (0.13, 0.29)	0.15 (0.10, 0.22)	-1.09 (-1.17, -1.01)	33262 (20472, 47360)	59040 (36358, 83890)	5.31 (3.28, 7.59)	3.81 (2.35, 5.42)	-1.20 (-1.28, -1.13)
Southeast Asia	1476 (924, 2045)	4342 (2706, 6292)	0.60 (0.37, 0.83)	0.67 (0.41, 0.97)	0.24 (0.14, 0.34)	42187 (26555, 58675)	120605 (75277, 174515)	15.30 (9.60, 21.20)	17.04 (10.63, 24.64)	0.26 (0.18, 0.35)
Australasia	286 (178, 394)	243 (146, 368)	1.22 (0.76, 1.69)	0.47 (0.28, 0.70)	-3.36 (-3.47, -3.24)	7628 (4856, 10493)	6027 (3756, 8907)	33.38 (21.29, 45.91)	12.76 (7.98, 18.72)	-3.35 (-3.49, -3.22)
Caribbean	148 (91, 204)	276 (169, 396)	0.58 (0.36, 0.80)	0.51 (0.31, 0.73)	-0.37 (-0.44, -0.29)	3885 (2410, 5334)	6960 (4301, 9974)	14.75 (9.15, 20.29)	12.88 (7.96, 18.42)	-0.41 (-0.48, -0.33)
Central Europe	1861 (1182, 2534)	2443 (1535, 3404)	1.23 (0.78, 1.67)	1.12 (0.71, 1.56)	-0.28 (-0.42, -0.14)	51858 (33141, 70065)	61418 (38703, 85319)	34.11 (21.82, 46.20)	30.13 (19.01, 41.72)	-0.38 (-0.52, -0.25)
Eastern Europe	2292 (1482, 3088)	2743 (1729, 3785)	0.80 (0.52, 1.08)	0.78 (0.49, 1.07)	-0.33 (-0.60, -0.06)	66869 (43459, 89804)	74533 (46979, 102675)	23.56 (15.33, 31.57)	21.99 (13.87, 30.20)	-0.48 (-0.73, -0.23)
Western Europe	7422 (4674, 10267)	6091 (3629, 8558)	1.28 (0.81, 1.77)	0.66 (0.40, 0.93)	-2.15 (-2.20, -2.10)	177329 (113098, 244518)	139254 (85368, 195593)	32.44 (20.75, 44.59)	17.16 (10.63, 24.04)	-2.04 (-2.11, -1.98)
Andean Latin America	45 (28, 65)	138 (80, 206)	0.23 (0.14, 0.33)	0.24 (0.14, 0.36)	0.27 (0.16, 0.38)	1154 (711, 1640)	3379 (1997, 5090)	5.52 (3.39, 7.87)	5.65 (3.34, 8.50)	0.22 (0.11, 0.33)

Central Latin America	233 (145, 322)	646 (389, 915)	0.29 (0.18, 0.40)	0.26 (0.16, 0.37)	-0.50 (-0.58, -0.43)	6286 (3974, 8628)	17200 (10490, 24270)	7.17 (4.51, 9.86)	6.68 (4.06, 9.44)	-0.35 (-0.43, -0.27)
Southern Latin America	466 (285, 649)	687 (411, 995)	1.00 (0.61, 1.39)	0.80 (0.48, 1.15)	-0.51 (-0.7, -0.32)	12850 (7978, 17684)	18224 (11206, 26040)	27.300 (16.95, 37.57)	21.74 (13.40, 30.97)	-0.53 (-0.7, -0.35)
Tropical Latin America	548 (345, 753)	1239 (753, 1773)	0.61 (0.38, 0.84)	0.48 (0.29, 0.69)	-0.94 (-1.03, -0.86)	15554 (9820, 21267)	33015 (20414, 46776)	15.77 (9.95, 21.58)	12.50 (7.72, 17.73)	-0.98 (-1.09, -0.87)
North Africa and Middle East	659 (425, 919)	1541 (935, 2170)	0.39 (0.25, 0.54)	0.34 (0.21, 0.48)	-0.34 (-0.46, -0.23)	19999 (12569, 27849)	44434 (27549, 62535)	10.57 (6.76, 14.72)	8.83 (5.44, 12.41)	-0.54 (-0.65, -0.42)
High-income North America	3746 (2324, 5233)	3516 (2108, 5006)	1.08 (0.67, 1.50)	0.55 (0.33, 0.78)	-2.30 (-2.38, -2.22)	95609 (59944, 132378)	90580 (54725, 125718)	28.82 (18.14, 39.75)	15.47 (9.48, 21.46)	-2.12 (-2.18, -2.05)
Oceania	8 (5, 11)	16 (10, 22)	0.26 (0.16, 0.38)	0.20 (0.12, 0.28)	-0.94 (-1.03, -0.85)	247 (143, 358)	492 (299, 694)	7.19 (4.24, 10.43)	5.53 (3.36, 7.80)	-0.95 (-1.03, -0.86)
Central Sub-Saharan Africa	35 (21, 52)	77 (45, 116)	0.16 (0.09, 0.23)	0.14 (0.08, 0.21)	-0.27 (-0.61, 0.07)	1035 (611, 1536)	2339 (1357, 3517)	4.13 (2.45, 6.09)	3.67 (2.15, 5.52)	-0.22 (-0.54, 0.10)
Eastern Sub-Saharan Africa	183 (109, 261)	332 (202, 480)	0.26 (0.15, 0.37)	0.22 (0.13, 0.31)	-0.66 (-0.77, -0.55)	5065 (3057, 7234)	9109 (5528, 13198)	6.42 (3.86, 9.18)	5.11 (3.11, 7.38)	-0.82 (-0.93, -0.72)
Southern Sub-Saharan Africa	109 (70, 155)	200 (126, 282)	0.41 (0.26, 0.59)	0.34 (0.22, 0.49)	-0.60 (-0.8, -0.41)	3143 (2027, 4453)	5881 (3730, 8303)	10.72 (6.90, 15.30)	9.23 (5.86, 13.06)	-0.42 (-0.60, -0.24)
Western Sub-Saharan Africa	63 (38, 92)	127 (75, 183)	0.08 (0.05, 0.11)	0.07 (0.04, 0.10)	-0.22 (-0.28, -0.15)	1725 (1030, 2510)	3526 (2035, 5084)	1.87 (1.12, 2.72)	1.66 (0.98, 2.38)	-0.30 (-0.37, -0.23)

DALYs: disability-adjusted life years; ASMR: age-standardized mortality rates; ASDR: age-standardized DALYs rates;

EAPC: estimated annual percentage change.

Table S4: the ASMR and ASDR of three GI cancers attributed to smoking in 2021 of 204 countries and territories.

Location	Esophageal cancer		Gastric cancer		Colorectal cancer	
	ASMR per 100000 (95% UI)	ASDR per 100000 (95% UI)	ASMR per 100000 (95% UI)	ASDR per 100000 (95% UI)	ASMR per 100000 (95% UI)	ASDR per 100000 (95% UI)
American Samoa	0.41(0.27, 0.55)	10.33(7.05, 14.02)	1.18(0.87, 1.58)	32.89(24.02, 44.01)	0.65(0.39, 0.97)	17.81(10.96, 26.62)
Antigua and Barbuda	0.54(0.38, 0.73)	12.27(8.65, 16.23)	0.52(0.39, 0.67)	11.48(8.85, 14.64)	0.36(0.21, 0.52)	8.55(5.04, 12.35)
Arab Republic of Egypt	0.42(0.30, 0.56)	9.03(6.60, 12.19)	0.80(0.49, 1.09)	17.71(10.39, 24.46)	0.39(0.23, 0.56)	9.96(5.98, 14.69)
Argentine Republic	1.17(0.86, 1.5)0	28.21(21.16, 35.35)	0.69(0.55, 0.85)	18.23(14.7, 22.23)	0.90(0.55, 1.29)	24.59(15.19, 35.28)
Australia	0.83(0.57, 1.11)	18.63(13.45, 24.32)	0.22(0.17, 0.28)	5.12(4.09, 6.27)	0.41(0.25, 0.61)	11.60(7.13, 17.09)
Barbados	0.85(0.54, 1.19)	17.74(11.50, 24.93)	0.37(0.25, 0.51)	7.65(5.18, 10.48)	0.41(0.23, 0.64)	9.62(5.36, 14.77)
Belize	0.42(0.30, 0.56)	10.42(7.53, 13.68)	0.48(0.37, 0.62)	11.68(9.09, 14.92)	0.29(0.17, 0.44)	7.36(4.42, 10.95)
Bermuda	0.87(0.59, 1.20)	20.64(14.23, 27.80)	0.30(0.22, 0.40)	6.80(5.03, 8.92)	0.62(0.36, 0.96)	15.25(8.93, 23.78)
Bolivarian Republic of Venezuela	0.27(0.16, 0.39)	6.30(3.75, 9.13)	0.53(0.37, 0.74)	13.15(9.04, 18.00)	0.30(0.17, 0.47)	7.97(4.47, 12.06)
Bosnia and Herzegovina	1.08(0.74, 1.46)	27.37(18.73, 37.40)	1.35(0.95, 1.87)	30.99(21.77, 42.80)	1.11(0.66, 1.65)	28.21(16.58, 42.28)
Brunei Darussalam	0.79(0.54, 1.08)	17.82(12.21, 24.52)	0.95(0.67, 1.29)	21.00(15.03, 28.49)	0.69(0.41, 1.02)	18.13(10.74, 26.74)
Burkina Faso	0.31(0.20, 0.45)	8.79(5.63, 12.68)	0.22(0.13, 0.30)	5.78(3.44, 7.84)	0.07(0.04, 0.11)	1.81(0.96, 2.70)
Canada	1.40(1.04, 1.79)	30.48(22.84, 38.76)	0.41(0.32, 0.52)	8.63(6.85, 10.83)	0.56(0.35, 0.81)	14.72(9.07, 21.22)
Central African Republic	0.91(0.61, 1.31)	25.46(16.85, 36.68)	0.38(0.23, 0.52)	10.65(6.61, 15.16)	0.16(0.08, 0.27)	4.46(2.22, 7.59)
Commonwealth of Dominica	0.69(0.47, 1.02)	17.49(11.65, 25.65)	0.78(0.56, 1.07)	19.06(13.78, 26.11)	0.39(0.22, 0.59)	9.58(5.65, 14.34)
Commonwealth of the Bahamas	1.09(0.72, 1.54)	27.07(17.77, 37.63)	0.41(0.29, 0.56)	9.83(6.94, 13.18)	0.48(0.27, 0.72)	12.40(6.94, 18.52)
Cook Islands	0.58(0.39, 0.83)	14.00(9.65, 19.99)	0.39(0.28, 0.53)	10.30(7.19, 13.8)	0.20(0.12, 0.29)	5.37(3.24, 7.92)
Cote dIvoire	0.15(0.10, 0.21)	3.90(2.58, 5.55)	0.09(0.06, 0.12)	2.32(1.47, 3.17)	0.06(0.03, 0.10)	1.63(0.87, 2.58)
Czech Republic	1.23(0.90, 1.57)	31.39(23.13, 40.19)	0.58(0.44, 0.73)	13.77(10.49, 17.52)	1.03(0.61, 1.53)	26.73(15.92, 39.92)
Democratic People's Republic of Korea	3.17(2.14, 4.6)0	86.32(58.56, 125.76)	2.13(1.48, 2.89)	60.68(41.48, 83.61)	0.46(0.25, 0.75)	14.04(7.59, 22.96)

Democratic Republic of Sao Tome and Principe	[illegible]	[illegible]	[illegible]	[illegible]	[illegible]	[illegible]
Democratic Republic of the Congo	0.50(0.31, 0.76)	13.89(8.42, 20.53)	0.20(0.13, 0.29)	5.54(3.48, 7.75)	0.10(0.05, 0.17)	2.59(1.35, 4.29)
Democratic Republic of Timor-Leste	0.49(0.32, 0.70)	12.08(7.81, 17.31)	0.62(0.41, 0.93)	15.09(9.89, 23.05)	0.42(0.24, 0.61)	10.43(6.02, 15.28)
Democratic Socialist Republic of Sri Lanka	0.53(0.31, 0.78)	12.96(7.39, 19.40)	0.18(0.11, 0.27)	4.45(2.57, 6.54)	0.15(0.07, 0.23)	3.55(1.78, 5.84)
Dominican Republic	0.69(0.46, 1.00)	15.54(10.44, 22.43)	0.61(0.42, 0.84)	13.36(9.30, 18.35)	0.41(0.24, 0.62)	9.51(5.69, 14.82)
Eastern Republic of Uruguay	1.74(1.32, 2.18)	41.72(32.13, 51.36)	1.00(0.78, 1.23)	25.53(20.32, 30.94)	1.26(0.75, 1.84)	33.25(19.91, 47.18)
Federal Democratic Republic of Ethiopia	0.15(0.10, 0.21)	3.73(2.58, 5.29)	0.10(0.06, 0.14)	2.42(1.59, 3.41)	0.18(0.11, 0.28)	4.16(2.42, 6.29)
Federal Democratic Republic of Nepal	0.81(0.55, 1.16)	18.01(12.17, 26.27)	0.50(0.33, 0.69)	11.03(7.43, 15.44)	0.21(0.12, 0.32)	4.65(2.70, 7.17)
Federal Republic of Germany	1.47(1.13, 1.82)	35.56(27.67, 43.48)	0.61(0.48, 0.77)	14.38(11.20, 17.74)	0.65(0.38, 0.93)	17.38(10.06, 24.52)
Federal Republic of Nigeria	0.19(0.12, 0.27)	4.81(3.11, 6.86)	0.03(0.02, 0.04)	0.70(0.47, 1.04)	0.04(0.02, 0.06)	1.01(0.57, 1.50)
Federal Republic of Somalia	1.18(0.74, 1.79)	31.70(19.5, 48.63)	0.39(0.23, 0.56)	10.45(6.04, 15.29)	0.19(0.09, 0.34)	4.98(2.24, 9.08)
Federated States of Micronesia	0.74(0.49, 1.04)	20.13(13.08, 28.52)	1.43(0.98, 2.02)	41.94(28.09, 58.74)	0.64(0.35, 0.99)	18.22(9.95, 28.20)
Federative Republic of Brazil	1.35(1.01, 1.72)	32.85(24.53, 41.58)	0.74(0.59, 0.92)	17.27(13.92, 21.20)	0.48(0.29, 0.68)	12.50(7.71, 17.77)
French Republic	1.17(0.90, 1.46)	28.03(22, 34.64)	0.40(0.31, 0.51)	9.84(7.75, 12.29)	0.66(0.40, 0.96)	17.66(10.63, 25.65)
Gabonese Republic	1.14(0.76, 1.56)	30.23(19.77, 42.52)	0.25(0.15, 0.33)	6.54(4.07, 8.88)	0.29(0.15, 0.47)	7.58(3.99, 11.93)
Georgia	0.48(0.37, 0.59)	11.74(9.03, 14.56)	1.58(1.27, 1.92)	39.94(32.36, 48.33)	0.75(0.45, 1.05)	20.80(12.90, 29.38)
Grand Duchy of Luxembourg	1.27(0.88, 1.75)	29.32(20.48, 39.99)	0.45(0.31, 0.63)	9.42(6.73, 13.05)	0.60(0.35, 0.88)	14.95(8.92, 21.53)
Greenland	4.48(2.99, 6.55)	108.64(74.85, 154.38)	1.11(0.78, 1.58)	28.23(20.22, 39.15)	1.55(0.93, 2.24)	42.29(25.46, 60.57)
Grenada	0.69(0.51, 0.91)	18.33(13.32, 23.77)	0.31(0.23, 0.39)	7.83(5.89, 9.95)	0.43(0.25, 0.65)	10.94(6.37, 16.15)
Guam	0.40(0.28, 0.53)	11.45(8.17, 15.33)	0.31(0.23, 0.40)	9.95(7.34, 12.62)	0.45(0.27, 0.65)	13.81(8.43, 19.93)

Hashemite Kingdom of Jordan	0.41(0.30, 0.54)	9.06(6.50, 11.90)	0.57(0.39, 0.76)	12.38(8.56, 16.62)	0.50(0.30, 0.77)	12.85(7.45, 19.96)
Hellenic Republic	0.87(0.71, 1.03)	20.89(17.12, 24.65)	1.24(1.01, 1.53)	27.37(22.21, 33.22)	0.84(0.51, 1.19)	21.00(12.88, 29.40)
Hungary	1.35(1.05, 1.72)	37.14(28.81, 46.84)	0.76(0.58, 0.97)	19.44(14.79, 24.57)	1.23(0.72, 1.81)	34.84(20.76, 50.51)
Independent State of Papua New Guinea	0.24(0.15, 0.38)	6.44(4.12, 10.20)	0.69(0.45, 0.99)	19.77(12.81, 29.11)	0.11(0.06, 0.16)	3.18(1.86, 4.74)
Independent State of Samoa	0.31(0.22, 0.43)	7.90(5.65, 10.69)	0.89(0.54, 1.18)	22.50(13.93, 29.54)	0.31(0.19, 0.46)	8.44(5.13, 12.26)
Ireland	1.88(1.39, 2.45)	38.89(29.15, 50.37)	0.45(0.34, 0.56)	8.87(7.12, 10.82)	0.59(0.36, 0.87)	14.36(8.86, 20.70)
Islamic Republic of Afghanistan	0.94(0.58, 1.41)	21.44(12.91, 32.69)	1.24(0.48, 1.83)	29.47(12.34, 43.15)	0.19(0.09, 0.33)	5.17(2.37, 8.99)
Islamic Republic of Iran	1.03(0.75, 1.29)	21.89(16.37, 27.67)	0.97(0.55, 1.25)	21.77(12.85, 27.37)	0.22(0.14, 0.32)	5.94(3.66, 8.49)
Islamic Republic of Mauritania	0.55(0.31, 0.84)	14.49(8.34, 22.46)	0.23(0.14, 0.32)	5.70(3.62, 7.95)	0.11(0.06, 0.17)	2.74(1.49, 4.21)
Islamic Republic of Pakistan	1.25(0.88, 1.74)	29.82(21.32, 41.16)	0.32(0.22, 0.45)	7.75(5.35, 10.90)	0.22(0.13, 0.33)	5.39(3.13, 8.08)
Jamaica	0.70(0.46, 1.02)	16.55(11.08, 24.49)	0.55(0.37, 0.78)	13.07(8.66, 18.72)	0.48(0.26, 0.75)	12.19(6.77, 19.16)
Japan	1.53(1.19, 1.86)	32.96(26.04, 39.85)	1.31(1.04, 1.62)	27.02(21.99, 32.52)	0.62(0.39, 0.86)	16.53(10.47, 22.64)
Kingdom of Bahrain	0.89(0.61, 1.25)	16.79(11.3, 24.10)	0.71(0.48, 1.02)	13.46(9.27, 19.40)	0.39(0.23, 0.61)	8.79(5.24, 13.88)
Kingdom of Belgium	1.73(1.33, 2.15)	40.23(30.99, 49.63)	0.50(0.39, 0.63)	11.07(8.68, 13.62)	0.57(0.34, 0.82)	14.85(8.86, 21.37)
Kingdom of Bhutan	0.57(0.33, 0.92)	11.90(6.92, 19.32)	0.26(0.16, 0.41)	5.60(3.36, 8.91)	0.11(0.06, 0.17)	2.51(1.34, 3.98)
Kingdom of Cambodia	1.08(0.75, 1.43)	25.63(17.86, 34.48)	1.35(0.94, 1.89)	30.43(20.83, 43.63)	0.85(0.51, 1.28)	20.79(12.03, 31.09)
Kingdom of Denmark	2.13(1.65, 2.64)	45.67(35.46, 55.88)	0.64(0.49, 0.80)	13.47(10.45, 16.61)	1.06(0.60, 1.56)	23.99(13.85, 34.91)
Kingdom of Eswatini	1.46(0.94, 2.08)	38.25(24.24, 55.59)	0.27(0.18, 0.37)	6.86(4.43, 9.58)	0.29(0.15, 0.46)	7.14(3.64, 11.09)
Kingdom of Lesotho	4.80(3.34, 6.68)	124.10(85.3, 172.32)	1.01(0.63, 1.39)	26.51(16.02, 36.59)	0.50(0.27, 0.78)	13.36(7.26, 20.75)
Kingdom of Morocco	0.15(0.09, 0.20)	3.53(2.27, 4.94)	0.12(0.07, 0.16)	2.88(1.84, 3.97)	0.17(0.09, 0.26)	4.63(2.49, 7.02)
Kingdom of Norway	0.74(0.53, 0.94)	16.36(12.21, 20.80)	0.23(0.18, 0.28)	5.10(4.08, 6.16)	0.53(0.32, 0.76)	13.54(8.56, 18.88)
Kingdom of Saudi Arabia	0.46(0.28, 0.67)	10.58(6.48, 15.43)	0.24(0.16, 0.39)	5.72(3.76, 9.97)	0.19(0.11, 0.28)	5.47(3.27, 8.19)
Kingdom of Spain	1.00(0.76, 1.21)	24.47(18.89, 29.51)	0.66(0.52, 0.83)	15.33(12.30, 18.64)	0.73(0.45, 1.05)	19.42(12.06, 27.36)
Kingdom of Sweden	1.01(0.73, 1.32)	21.09(15.31, 27.47)	0.35(0.26, 0.44)	7.04(5.38, 8.97)	0.59(0.35, 0.87)	14.06(8.20, 20.60)
Kingdom of Thailand	1.24(0.85, 1.78)	33.35(22.65, 48.03)	0.600(0.29, 0.88)	15.79(7.48, 23.33)	0.76(0.42, 1.22)	19.97(10.90, 32.12)

Kingdom of the Netherlands	2.37(1.77, 2.96)	49.58(37.83, 61.41)	0.51(0.4, 0.64)	10.29(8.11, 12.63)	1.06(0.64, 1.54)	26.28(16.17, 37.54)
Kingdom of Tonga	0.73(0.50, 1.04)	16.87(11.75, 23.94)	1.65(1.18, 2.31)	39.48(27.82, 55.38)	0.31(0.17, 0.46)	7.51(4.34, 11.27)
Kyrgyz Republic	1.20(0.93, 1.54)	28.44(21.51, 36.84)	2.15(1.63, 2.76)	56.10(42.60, 72.16)	0.31(0.19, 0.47)	9.03(5.62, 13.05)
Lao People's Democratic Republic	0.82(0.59, 1.13)	19.68(13.89, 27.32)	0.95(0.66, 1.36)	22.20(15.55, 31.49)	0.77(0.45, 1.15)	19.17(11.02, 28.98)
Lebanese Republic	0.49(0.36, 0.65)	10.70(7.83, 14.43)	0.87(0.61, 1.18)	18.76(13.50, 25.25)	0.69(0.4, 1.02)	16.46(9.78, 23.88)
Malaysia	0.71(0.52, 0.93)	16.27(12.01, 21.15)	0.45(0.34, 0.60)	10.42(8.09, 13.89)	0.69(0.42, 1.00)	16.8(10.27, 23.95)
Mongolia	3.90(2.70, 5.17)	91.69(63.92, 122.63)	3.42(2.44, 4.49)	92.89(66.29, 126.46)	0.37(0.22, 0.57)	10.55(6.03, 16.02)
Montenegro	1.18(0.82, 1.63)	30.31(21.02, 41.27)	1.16(0.82, 1.57)	27.15(19.45, 36.66)	0.99(0.62, 1.47)	26.24(16.31, 38.68)
New Zealand	0.83(0.62, 1.08)	16.94(12.84, 21.63)	0.36(0.29, 0.45)	8.44(6.84, 10.39)	0.78(0.48, 1.18)	18.93(11.60, 28.28)
North Macedonia	0.70(0.49, 0.94)	17.32(11.83, 23.78)	1.95(1.36, 2.66)	44.71(31.05, 60.37)	1.01(0.60, 1.48)	25.91(15.52, 38.24)
Northern Mariana Islands	0.59(0.41, 0.84)	14.75(10.62, 20.20)	0.77(0.58, 1.00)	20.53(15.67, 26.66)	0.65(0.40, 0.91)	17.18(10.68, 24.62)
Palestine	0.38(0.26, 0.52)	7.88(5.36, 11.00)	0.72(0.47, 1.01)	15.38(10.38, 21.10)	0.61(0.36, 0.86)	15.06(8.81, 21.26)
People's Democratic Republic of Algeria	0.28(0.19, 0.38)	5.54(3.75, 7.73)	0.44(0.29, 0.61)	8.23(5.49, 11.43)	0.19(0.11, 0.29)	4.18(2.36, 6.38)
People's Republic of Bangladesh	0.79(0.50, 1.12)	18.46(12.05, 26.15)	0.55(0.34, 0.76)	12.98(8.02, 18.22)	0.22(0.13, 0.34)	5.24(3.07, 8.20)
People's Republic of China	6.56(4.82, 8.58)	145.42(106.18, 191.29)	3.10(2.27, 4.30)	71.07(52.06, 99.29)	0.82(0.50, 1.21)	21.44(12.95, 31.98)
Plurinational State of Bolivia	0.31(0.20, 0.47)	6.92(4.33, 10.40)	1.23(0.85, 1.82)	28.95(19.61, 42.84)	0.35(0.18, 0.58)	8.24(4.36, 13.82)
Portuguese Republic	0.93(0.70, 1.17)	25.71(19.79, 32.03)	0.71(0.56, 0.87)	18.69(15.15, 22.64)	0.56(0.34, 0.79)	16.27(10.11, 23.10)
Principality of Andorra	0.47(0.25, 0.75)	11.07(5.88, 17.45)	0.63(0.38, 0.97)	13.89(8.33, 21.26)	0.80(0.41, 1.24)	20.18(10.66, 31.26)
Principality of Monaco	1.88(1.18, 2.87)	44.16(28.56, 67.32)	0.76(0.50, 1.12)	16.97(11.08, 25.42)	1.11(0.63, 1.69)	29.19(16.52, 45.02)
Puerto Rico	0.52(0.33, 0.81)	12.46(8.12, 18.89)	0.24(0.17, 0.36)	5.76(4.05, 8.34)	0.45(0.26, 0.70)	12.42(7.09, 18.94)
Republic of Albania	0.77(0.52, 1.07)	17.73(11.97, 24.54)	2.07(1.47, 2.93)	42.55(29.86, 58.76)	0.46(0.27, 0.69)	10.17(6.03, 15.49)
Republic of Angola	1.31(0.90, 1.85)	33.98(23.48, 48.08)	0.39(0.26, 0.53)	10.21(6.67, 13.96)	0.22(0.13, 0.35)	5.91(3.41, 9.37)
Republic of Armenia	0.49(0.40, 0.59)	11.55(9.4, 13.83)	1.78(1.43, 2.16)	41.75(33.89, 50.55)	0.68(0.42, 0.94)	17.49(10.92, 23.98)

Republic of Austria	0.97(0.76, 1.21)	23.40(18.61, 28.87)	0.49(0.38, 0.61)	11.57(9.20, 14.33)	0.52(0.30, 0.73)	14.00(8.15, 19.84)
Republic of Azerbaijan	2.29(1.59, 3.07)	52.66(36.33, 70.98)	2.02(1.44, 3.02)	47.87(33.71, 71.83)	0.34(0.19, 0.52)	9.37(5.27, 14.29)
Republic of Belarus	1.39(1.02, 1.78)	39.05(28.26, 49.96)	1.60(1.16, 2.10)	42.27(30.73, 55.20)	0.92(0.54, 1.36)	24.86(14.46, 36.74)
Republic of Benin	0.35(0.23, 0.50)	9.01(5.99, 12.88)	0.17(0.10, 0.23)	4.17(2.55, 5.66)	0.06(0.03, 0.09)	1.43(0.79, 2.22)
Republic of Botswana	1.97(1.31, 2.85)	47.78(31.48, 68.59)	0.37(0.23, 0.51)	9.03(5.68, 12.71)	0.40(0.23, 0.60)	9.60(5.39, 14.75)
Republic of Bulgaria	1.00(0.75, 1.27)	28.91(22.11, 36.28)	1.35(1.02, 1.76)	36.89(28.19, 47.95)	1.25(0.77, 1.85)	37.03(22.94, 54.98)
Republic of Burundi	0.76(0.51, 1.06)	20.30(13.60, 28.51)	0.23(0.15, 0.33)	6.14(3.78, 8.65)	0.17(0.09, 0.26)	4.22(2.32, 6.78)
Republic of Cabo Verde	1.45(0.98, 2.06)	39.26(26.68, 56.40)	0.55(0.38, 0.77)	13.97(9.60, 20.02)	0.11(0.06, 0.17)	2.78(1.48, 4.24)
Republic of Cameroon	0.57(0.34, 0.85)	16.18(9.64, 24.35)	0.23(0.13, 0.32)	6.06(3.53, 8.78)	0.10(0.05, 0.16)	2.69(1.28, 4.23)
Republic of Chad	0.51(0.32, 0.77)	13.16(8.05, 20.07)	0.38(0.25, 0.54)	9.30(5.94, 13.39)	0.11(0.06, 0.17)	2.59(1.37, 4.12)
Republic of Chile	0.44(0.32, 0.57)	10.54(8.02, 13.38)	0.84(0.65, 1.04)	22.90(18.14, 27.97)	0.47(0.28, 0.70)	13.58(8.28, 19.66)
Republic of Colombia	0.19(0.14, 0.27)	4.34(3.09, 5.85)	0.47(0.35, 0.62)	12.22(9.10, 16.20)	0.24(0.14, 0.36)	6.31(3.77, 9.47)
Republic of Costa Rica	0.35(0.25, 0.46)	7.32(5.25, 9.57)	0.99(0.76, 1.25)	22.93(17.65, 28.56)	0.50(0.29, 0.74)	12.84(7.66, 18.64)
Republic of Croatia	1.25(0.96, 1.55)	31.42(24.07, 39.20)	1.23(0.93, 1.56)	26.90(20.45, 34.07)	1.39(0.85, 2.00)	34.16(21.12, 48.26)
Republic of Cuba	1.78(1.31, 2.27)	46.00(34.35, 58.17)	0.52(0.4, 0.66)	12.53(9.66, 15.91)	0.70(0.42, 1.01)	18.28(11.21, 25.82)
Republic of Cyprus	0.61(0.42, 0.82)	13.62(9.47, 18.54)	0.69(0.47, 0.94)	14.37(9.90, 19.38)	0.56(0.34, 0.83)	13.74(8.41, 20.02)
Republic of Djibouti	2.35(1.41, 3.59)	56.95(33.89, 86.80)	0.51(0.32, 0.73)	12.14(7.62, 17.18)	0.58(0.32, 0.92)	13.35(7.27, 21.08)
Republic of Ecuador	0.17(0.12, 0.24)	3.52(2.44, 4.79)	0.73(0.53, 0.99)	17.21(12.32, 23.16)	0.24(0.13, 0.37)	5.79(3.27, 8.71)
Republic of El Salvador	0.22(0.14, 0.32)	5.47(3.61, 7.88.00)	0.6(0.42, 0.82)	16.33(11.68, 22.07)	0.17(0.10, 0.26)	5.11(3.02, 7.71)
Republic of Equatorial Guinea	0.99(0.62, 1.45)	25.91(15.71, 38.00)	0.21(0.13, 0.3)	5.51(3.30, 7.84)	0.24(0.13, 0.39)	6.10(3.20, 9.94)
Republic of Estonia	1.03(0.77, 1.32)	26.91(20.19, 34.55)	0.91(0.7, 1.17)	23.61(18.11, 29.72)	0.75(0.47, 1.08)	19.80(12.32, 28.63)
Republic of Fiji	0.46(0.31, 0.65)	11.82(8.02, 16.92)	0.43(0.18, 0.63)	11.61(4.76, 17.23)	0.36(0.19, 0.58)	9.62(5.01, 15.16)
Republic of Finland	0.71(0.52, 0.92)	17.45(13.09, 22.27)	0.27(0.2, 0.35)	6.52(4.95, 8.30)	0.38(0.21, 0.55)	10.21(5.81, 14.69)
Republic of Ghana	0.22(0.15, 0.30)	5.22(3.55, 7.15)	0.14(0.1, 0.19)	3.19(2.18, 4.42)	0.09(0.05, 0.14)	2.01(1.19, 3.02)
Republic of Guatemala	0.19(0.13, 0.26)	4.18(2.92, 5.64)	0.75(0.56, 0.98)	18.29(13.69, 23.85)	0.15(0.09, 0.23)	3.89(2.24, 5.68)
Republic of Guinea	0.14(0.09, 0.22)	3.73(2.36, 5.56)	0.29(0.19, 0.41)	7.34(4.79, 10.34)	0.09(0.05, 0.15)	2.32(1.26, 3.71)

Republic of Guinea-Bissau	0.54(0.36, 0.76)	15.20(9.93, 22.00)	0.30(0.19, 0.39)	8.13(5.33, 10.95)	0.10(0.05, 0.15)	2.57(1.35, 3.99)
Republic of Guyana	0.30(0.20, 0.44)	8.50(5.53, 12.40)	0.33(0.22, 0.46)	8.86(6.03, 12.70)	0.37(0.20, 0.58)	10.00(5.38, 15.81)
Republic of Haiti	0.29(0.18, 0.45)	7.68(4.76, 12.00)	0.49(0.31, 0.71)	12.44(7.66, 17.91)	0.26(0.12, 0.42)	6.25(3.08, 10.19)
Republic of Honduras	0.35(0.24, 0.49)	7.53(5.12, 10.56)	1.01(0.70, 1.39)	23.34(16.55, 31.96)	0.18(0.11, 0.27)	4.53(2.67, 6.70)
Republic of Iceland	1.61(1.16, 2.09)	36.49(26.89, 46.47)	0.50(0.37, 0.65)	10.43(7.84, 13.36)	0.40(0.24, 0.58)	10.02(5.96, 14.54)
Republic of India	0.51(0.38, 0.65)	12.29(9.25, 15.69)	0.36(0.27, 0.47)	8.68(6.52, 11.60)	0.14(0.08, 0.200)	3.47(2.03, 5.07)
Republic of Indonesia	0.37(0.27, 0.49)	9.12(6.62, 11.97)	0.65(0.48, 0.90)	15.90(11.43, 21.93)	0.65(0.36, 1.02)	16.05(8.94, 25.30)
Republic of Iraq	0.50(0.33, 0.72)	11.16(7.11, 16.00)	0.70(0.46, 0.94)	14.99(9.82, 20.35)	0.34(0.2, 0.51)	8.44(4.82, 12.78)
Republic of Italy	0.57(0.44, 0.72)	12.95(10.15, 16.14)	0.69(0.53, 0.85)	14.94(11.86, 18.23)	0.58(0.36, 0.81)	14.94(9.57, 20.69)
Republic of Kazakhstan	1.13(0.85, 1.46)	28.43(21.61, 36.33)	0.84(0.66, 1.06)	23.63(18.79, 29.24)	0.35(0.22, 0.49)	10.33(6.41, 14.40)
Republic of Kenya	1.04(0.71, 1.53)	26.02(17.69, 38.81)	0.27(0.20, 0.37)	6.74(4.90, 9.1)0	0.14(0.08, 0.21)	3.58(2.13, 5.36)
Republic of Kiribati	2.19(1.52, 3.05)	56.09(38.42, 78.25)	2.53(1.78, 3.34)	69.91(48.83, 92.78)	0.64(0.39, 0.96)	17.52(10.51, 26.63)
Republic of Korea	1.17(0.83, 1.58)	23.94(16.76, 32.42)	1.78(1.30, 2.40)	34.86(26.25, 46.05)	0.56(0.33, 0.81)	13.03(7.91, 18.96)
Republic of Latvia	1.12(0.84, 1.39)	31.08(23.38, 38.08)	1.02(0.77, 1.31)	28.58(21.59, 36.25)	0.76(0.48, 1.09)	21.27(13.65, 30.37)
Republic of Liberia	0.43(0.27, 0.62)	11.85(7.45, 17.44)	0.21(0.13, 0.31)	5.67(3.40, 8.22)	0.08(0.03, 0.13)	1.98(0.89, 3.46)
Republic of Lithuania	1.29(0.99, 1.60)	35.22(27.29, 43.48)	0.94(0.71, 1.19)	25.09(19.03, 31.67)	0.73(0.45, 1.05)	19.36(11.94, 27.68)
Republic of Madagascar	0.60(0.38, 0.90)	14.83(9.47, 22.77)	0.14(0.09, 0.21)	3.56(2.22, 5.22)	0.13(0.07, 0.20)	3.04(1.58, 4.77)
Republic of Malawi	3.90(2.68, 5.32)	98.92(66.79, 136.56)	0.18(0.12, 0.25)	4.36(2.93, 6.01)	0.15(0.09, 0.24)	3.62(2.07, 5.76)
Republic of Maldives	0.45(0.32, 0.60)	9.40(6.61, 12.57)	0.38(0.27, 0.53)	7.49(5.34, 10.34)	0.27(0.16, 0.40)	5.79(3.38, 8.61)
Republic of Mali	0.26(0.17, 0.36)	6.24(4.00, 8.65)	0.57(0.40, 0.78)	13.25(9.27, 18.48)	0.17(0.10, 0.28)	3.88(2.26, 6.21)
Republic of Malta	0.83(0.62, 1.08)	20.78(15.76, 26.69)	0.47(0.36, 0.61)	10.91(8.44, 14.03)	0.46(0.26, 0.67)	12.36(7.31, 17.76)
Republic of Mauritius	0.87(0.69, 1.05)	22.39(17.90, 27.00)	0.78(0.64, 0.95)	19.87(16.200, 23.92)	0.64(0.42, 0.9)	16.77(10.77, 23.37)
Republic of Moldova	0.99(0.77, 1.22)	27.37(21.25, 33.87)	1.21(0.90, 1.60)	32.86(24.39, 42.91)	0.85(0.52, 1.23)	24.23(14.82, 34.58)
Republic of Mozambique	1.12(0.77, 1.51)	27.12(18.60, 37)	0.29(0.19, 0.40)	7.08(4.64, 9.75)	0.09(0.05, 0.14)	2.04(1.200, 3.02)
Republic of Namibia	0.37(0.26, 0.48)	8.81(6.18, 11.97)	0.15(0.11, 0.20)	3.45(2.45, 4.61)	0.24(0.15, 0.34)	5.27(3.20, 7.50)
Republic of Nauru	0.79(0.51, 1.10)	21.49(13.79, 30.3)	1.51(1.03, 2.03)	45.03(30.78, 61.49)	0.80(0.41, 1.25)	23.38(11.85, 35.75)

Republic of Nicaragua	0.16(0.11, 0.23)	3.57(2.41, 5.06)	0.47(0.34, 0.64)	11.55(8.33, 15.65)	0.16(0.09, 0.24)	4.23(2.49, 6.24)
Republic of Niue	0.60(0.40, 0.92)	14.91(9.87, 22.40)	0.67(0.47, 0.94)	17.82(12.62, 24.85)	0.41(0.24, 0.60)	11.07(6.55, 16.51)
Republic of Palau	0.51(0.35, 0.74)	12.55(8.68, 17.94)	0.96(0.67, 1.33)	26.18(18.25, 35.94)	0.25(0.14, 0.36)	6.27(3.63, 9.20)
Republic of Panama	0.21(0.14, 0.30)	4.35(2.88, 6.17)	0.41(0.30, 0.55)	9.21(6.67, 12.41)	0.25(0.14, 0.38)	6.33(3.69, 9.47)
Republic of Paraguay	1.32(0.86, 1.95)	30.27(19.74, 44.54)	0.82(0.55, 1.16)	17.93(12.10, 25.04)	0.55(0.32, 0.86)	12.84(7.39, 19.48)
Republic of Peru	0.14(0.09, 0.21)	2.90(1.90, 4.40)	0.64(0.43, 0.88)	15.00(10.07, 20.72)	0.21(0.11, 0.33)	4.86(2.69, 7.63)
Republic of Poland	1.17(0.91, 1.45)	30.61(24.19, 37.65)	0.90(0.72, 1.11)	22.28(18.06, 27.10)	1.18(0.73, 1.66)	30.59(19.23, 42.71)
Republic of Rwanda	2.59(1.75, 3.66)	58.08(39.06, 80.76)	0.53(0.36, 0.73)	11.88(8.13, 16.24)	0.48(0.28, 0.77)	10.42(5.94, 16.61)
Republic of San Marino	0.34(0.19, 0.55)	8.00(4.35, 13.10)	0.98(0.56, 1.63)	20.92(11.88, 34.65)	0.43(0.22, 0.71)	10.89(5.63, 18.07)
Republic of Senegal	0.49(0.32, 0.71)	13.55(8.98, 20.01)	0.24(0.15, 0.33)	6.45(4.07, 9.07)	0.09(0.05, 0.14)	2.46(1.28, 3.83)
Republic of Serbia	0.89(0.58, 1.31)	23.72(15.30, 35.08)	0.88(0.64, 1.19)	22.43(16.23, 30.39)	1.18(0.70, 1.72)	32.14(19.51, 46.56)
Republic of Seychelles	1.67(1.24, 2.20)	41.17(30.12, 54.52)	0.59(0.44, 0.8)	14.23(10.51, 18.96)	0.89(0.56, 1.27)	22.55(14.22, 31.79)
Republic of Sierra Leone	0.49(0.34, 0.68)	13.66(9.28, 19.38)	0.31(0.20, 0.43)	8.26(5.35, 11.52)	0.10(0.06, 0.15)	2.53(1.49, 3.98)
Republic of Singapore	0.41(0.29, 0.54)	9.04(6.51, 11.64)	0.22(0.17, 0.29)	4.70(3.66, 5.91)	0.27(0.17, 0.39)	7.13(4.45, 10.05)
Republic of Slovenia	0.95(0.68, 1.22)	24.79(17.85, 31.65)	0.74(0.55, 0.98)	17.64(13.25, 23.18)	0.58(0.34, 0.87)	15.68(9.16, 23.10)
Republic of South Africa	1.84(1.38, 2.32)	49.67(37.68, 62.74)	0.32(0.24, 0.40)	9.00(6.92, 11.06)	0.32(0.20, 0.46)	8.87(5.59, 12.52)
Republic of South Sudan	1.45(0.91, 2.16)	37.07(22.68, 56.08)	0.34(0.20, 0.50)	8.66(4.79, 12.74)	0.28(0.13, 0.44)	6.64(3.23, 10.76)
Republic of Sudan	1.38(0.82, 2.14)	30.28(17.87, 46.64)	1.18(0.41, 1.86)	25.85(9.42, 41.43)	0.15(0.08, 0.26)	4.01(2.05, 6.86)
Republic of Suriname	0.42(0.28, 0.61)	11.26(7.48, 16.16)	0.47(0.31, 0.71)	12.58(8.35, 18.77)	0.53(0.30, 0.83)	14.42(8.29, 22.20)
Republic of Tajikistan	2.01(1.17, 3.17)	43.30(25.63, 68.13)	1.19(0.79, 1.76)	28.60(19.20, 42.42)	0.12(0.07, 0.18)	3.26(1.90, 5.09)
Republic of the Congo	1.36(0.89, 1.97)	33.72(21.96, 48.60)	0.31(0.19, 0.41)	7.75(4.88, 10.50)	0.24(0.14, 0.35)	5.91(3.52, 9.02)
Republic of the Gambia	0.21(0.14, 0.30)	5.75(3.75, 8.11)	0.11(0.07, 0.15)	3.00(1.95, 4.12)	0.05(0.03, 0.08)	1.32(0.73, 2.06)
Republic of the Marshall Islands	0.56(0.33, 0.89)	14.26(8.63, 22.32)	1.02(0.65, 1.44)	28.63(18.69, 40.50)	0.39(0.22, 0.60)	10.68(6.03, 16.73)
Republic of the Niger	0.20(0.13, 0.29)	4.63(3.00, 6.73)	0.19(0.12, 0.26)	4.02(2.44, 5.59)	0.05(0.02, 0.08)	1.03(0.52, 1.65)
Republic of the Philippines	0.42(0.31, 0.53)	10.61(7.84, 13.70)	0.40(0.30, 0.57)	10.20(7.56, 14.41)	0.71(0.43, 1.01)	19.06(11.64, 26.59)

Republic of the Union of Myanmar	0.53(0.37, 0.75)	12.38(8.47, 17.48)	0.58(0.42, 0.79)	13.44(9.65, 18.51)	0.56(0.33, 0.83)	13.38(7.75, 19.99)
Republic of Trinidad and Tobago	0.40(0.26, 0.57)	10.56(6.99, 14.9)	0.28(0.19, 0.38)	7.36(5.04, 10.19)	0.49(0.28, 0.77)	13.42(7.63, 20.87)
Republic of Tunisia	0.28(0.18, 0.41)	6.26(3.84, 9.35)	0.55(0.35, 0.77)	11.67(7.30, 16.37)	0.38(0.21, 0.60)	9.12(5.24, 14.44)
Republic of Turkey	0.61(0.42, 0.82)	14.10(9.56, 19.11)	1.40(0.98, 1.93)	33.06(22.68, 45.92)	0.60(0.35, 0.88)	16.17(9.53, 23.97)
Republic of Uganda	1.14(0.81, 1.61)	29.28(20.53, 40.59)	0.17(0.12, 0.24)	4.29(3.06, 6.03)	0.19(0.11, 0.29)	4.66(2.81, 7.18)
Republic of Uzbekistan	0.64(0.46, 0.85)	15.35(11.05, 20.12)	0.44(0.32, 0.57)	12.26(9.05, 15.84)	0.14(0.08, 0.21)	4.23(2.49, 6.19)
Republic of Vanuatu	0.37(0.25, 0.51)	9.37(6.42, 13.06)	0.81(0.57, 1.13)	22.19(15.41, 31.5)	0.30(0.17, 0.46)	7.90(4.45, 12.06)
Republic of Yemen	1.48(0.89, 2.18)	33.66(20.34, 50.21)	2.22(0.81, 3.29)	51.04(18.69, 75.41)	0.27(0.14, 0.42)	7.10(3.68, 11.13)
Republic of Zambia	1.63(1.08, 2.34)	40.22(26.50, 57.04)	0.27(0.18, 0.38)	6.69(4.38, 9.29)	0.34(0.17, 0.67)	8.15(4.00, 17.35)
Republic of Zimbabwe	3.70(2.55, 4.96)	92.25(63.21, 124.29)	0.80(0.58, 1.06)	20.16(14.5, 26.98)	0.49(0.30, 0.71)	12.32(7.49, 18.51)
Romania	0.92(0.71, 1.17)	26.82(20.78, 33.45)	1.00(0.77, 1.27)	27.35(21.18, 33.96)	1.04(0.62, 1.48)	29.52(17.57, 41.85)
Russian Federation	1.29(1.03, 1.56)	35.84(28.69, 43.19)	1.34(1.08, 1.61)	35.89(29.32, 43.07)	0.81(0.52, 1.09)	22.70(14.85, 30.43)
Saint Kitts and Nevis	0.56(0.37, 0.78)	13.20(9.00, 18.23)	0.32(0.23, 0.43)	7.21(5.06, 9.66)	0.36(0.20, 0.54)	8.57(4.79, 12.70)
Saint Lucia	0.83(0.56, 1.12)	20.42(14.13, 27.89)	0.52(0.38, 0.69)	12.44(9.23, 16.57)	0.29(0.16, 0.45)	7.33(4.14, 11.13)
Saint Vincent and the Grenadines	0.40(0.29, 0.54)	10.45(7.56, 13.95)	0.44(0.33, 0.57)	11.22(8.51, 14.41)	0.35(0.21, 0.51)	8.83(5.28, 12.77)
Slovak Republic	1.11(0.76, 1.57)	30.37(20.29, 43.82)	0.72(0.50, 0.97)	17.98(12.72, 23.88)	1.19(0.73, 1.76)	31.74(19.29, 46.11)
Socialist Republic of Viet Nam	0.89(0.63, 1.18)	23.34(16.16, 31.55)	0.82(0.59, 1.18)	21.42(15.36, 29.92)	0.76(0.47, 1.07)	20.21(12.33, 29.25)
Solomon Islands	0.71(0.46, 1.05)	18.88(11.96, 28.37)	1.69(1.17, 2.36)	48.69(33.65, 68.67)	0.48(0.26, 0.74)	13.60(7.27, 20.79)
State of Eritrea	0.72(0.42, 1.20)	20.73(11.72, 34.75)	0.17(0.11, 0.25)	5.17(3.13, 7.68)	0.13(0.07, 0.21)	3.73(2.00, 6.30)
State of Israel	0.47(0.35, 0.60)	10.39(7.88, 13.17)	0.46(0.36, 0.59)	10.18(8.07, 12.64)	0.46(0.28, 0.68)	11.48(6.93, 16.62)
State of Kuwait	0.38(0.27, 0.50)	7.42(5.23, 9.75)	0.30(0.22, 0.41)	6.08(4.33, 8.19)	0.33(0.20, 0.51)	8.06(4.82, 12.04)
State of Libya	0.80(0.53, 1.13)	19.23(12.57, 27.18)	0.63(0.40, 0.89)	14.42(9.39, 20.26)	0.52(0.29, 0.79)	13.45(7.44, 20.62)
State of Qatar	0.73(0.41, 1.11)	14.50(8.21, 22.48)	0.37(0.23, 0.55)	7.76(4.8, 11.48)	0.23(0.13, 0.37)	5.60(3.14, 9.11)
Sultanate of Oman	0.31(0.20, 0.44)	6.81(4.45, 9.98)	0.25(0.17, 0.34)	5.41(3.66, 7.34)	0.09(0.05, 0.13)	2.11(1.20, 3.30)
Swiss Confederation	1.18(0.90, 1.49)	25.05(19.24, 30.96)	0.44(0.34, 0.57)	9.13(7.22, 11.63)	0.49(0.30, 0.72)	11.75(7.31, 17.54)

Syrian Arab Republic	0.34(0.23, 0.47)	6.99(4.57, 9.85)	0.58(0.39, 0.80)	12.30(8.27, 17.23)	0.25(0.13, 0.38)	6.30(3.26, 9.73)
Taiwan (Province of China)	3.26(2.59, 3.91)	91.73(72.61, 111.37)	1.03(0.79, 1.30)	20.82(16.26, 26.05)	0.90(0.57, 1.26)	23.24(14.87, 31.92)
Togolese Republic	1.02(0.65, 1.50)	26.34(16.66, 39.08)	0.44(0.27, 0.64)	11.11(6.73, 16.06)	0.11(0.06, 0.18)	2.90(1.56, 4.66)
Tokelau	0.44(0.28, 0.64)	10.83(7.00, 15.56)	0.64(0.47, 0.88)	17.28(12.48, 24.42)	0.36(0.20, 0.55)	9.67(5.46, 15.39)
Turkmenistan	2.32(1.60, 3.18)	55.78(38.24, 76.87)	0.82(0.58, 1.13)	21.60(15.28, 29.74)	0.13(0.08, 0.20)	4.05(2.33, 6.16)
Tuvalu	0.55(0.38, 0.79)	14.29(9.94, 20.38)	1.06(0.78, 1.43)	29.76(22.05, 39.91)	0.46(0.28, 0.67)	12.72(7.63, 18.48)
Ukraine	0.90(0.57, 1.34)	26.65(16.64, 39.84)	0.99(0.64, 1.44)	28.39(18.02, 41.41)	0.66(0.36, 1.09)	19.29(10.48, 31.47)
Union of the Comoros	1.46(0.95, 2.25)	34.41(21.53, 54.06)	0.29(0.18, 0.42)	6.63(4.25, 9.65)	0.30(0.17, 0.47)	6.58(3.56, 10.39)
United Arab Emirates	0.54(0.35, 0.77)	9.63(6.16, 13.72)	0.43(0.29, 0.61)	7.91(5.50, 11.21)	0.27(0.15, 0.46)	5.85(3.14, 9.97)
United Kingdom of Great Britain and Northern Ireland	2.71(2.02, 3.37)	55.72(42.34, 69.13)	0.51(0.39, 0.64)	10.01(7.92, 12.20)	0.72(0.44, 1.03)	17.48(10.79, 24.52)
United Mexican States	0.21(0.15, 0.27)	4.69(3.47, 5.95)	0.38(0.29, 0.48)	8.97(6.98, 11.22)	0.27(0.16, 0.38)	6.82(4.19, 9.59)
United Republic of Tanzania	1.26(0.87, 1.80)	31.81(21.62, 45.89)	0.29(0.19, 0.41)	7.46(4.81, 10.54)	0.35(0.2, 0.54)	8.58(4.92, 13.35)
United States of America	1.66(1.26, 2.07)	38.43(29.99, 47.02)	0.35(0.27, 0.43)	8.09(6.52, 9.85)	0.55(0.33, 0.78)	15.55(9.50, 21.59)
United States Virgin Islands	0.47(0.26, 0.77)	11.20(6.28, 18.31)	0.29(0.17, 0.43)	6.60(4.05, 9.67)	0.32(0.17, 0.50)	8.26(4.64, 12.81)

GI: gastrointestinal; DALYs: disability-adjusted life years; ASMR: age-standardized mortality rates; ASDR: age-standardized DALYs rates;

Table S5: the EAPC of ASMR and ASDR of three GI cancers attributed to smoking in 2021 of 204 countries and territories.

Location	Esophageal cancer		Gastric cancer		Colorectal cancer	
	EAPC of ASMR from 1990-2021 (95%UI)	EAPC of ASDR from 1990-2021 (95% UI)	EAPC of ASMR from 1990-2021 (95% UI)	EAPC of ASDR from 1990-2021 (95% UI)	EAPC of ASMR from 1990-2021 (95% UI)	EAPC of ASDR from 1990-2021 (95% UI)
American Samoa	0.94 (0.61, 1.26)	1.00 (0.67, 1.32)	-1.20 (-1.32, -1.08)	-1.11 (-1.23, -0.99)	-0.26 (-0.40, -0.13)	-0.22 (-0.35, -0.10)
Antigua and Barbuda	-0.59 (-0.88, -0.30)	-0.74 (-1.02, -0.46)	-2.26 (-2.47, -2.06)	-2.44 (-2.64, -2.24)	0.47 (0.27, 0.67)	0.35 (0.18, 0.53)
Arab Republic of Egypt	0.09 (-0.08, 0.26)	-0.25 (-0.43, -0.06)	2.97 (2.29, 3.65)	2.53 (1.91, 3.15)	2.20 (1.91, 2.48)	1.82 (1.59, 2.06)
Argentine Republic	-2.25 (-2.53, -1.97)	-2.46 (-2.74, -2.18)	-1.99 (-2.18, -1.79)	-2.04 (-2.25, -1.83)	-0.39 (-0.63, -0.15)	-0.43 (-0.67, -0.20)
Australia	-2.18 (-2.29, -2.08)	-2.25 (-2.35, -2.16)	-3.98 (-4.07, -3.89)	-3.92 (-4.02, -3.83)	-3.51 (-3.63, -3.40)	-3.40 (-3.53, -3.27)
Barbados	-1.61 (-1.84, -1.37)	-1.70 (-1.90, -1.50)	-3.27 (-3.60, -2.93)	-3.3 (-3.58, -3.01)	0.06 (-0.23, 0.36)	-0.06 (-0.33, 0.21)
Belize	0.28 (-0.21, 0.77)	0.31 (-0.17, 0.78)	-1.49 (-2.06, -0.91)	-1.47 (-2.03, -0.90)	1.12 (0.65, 1.60)	1.14 (0.68, 1.60)
Bermuda	-1.39 (-1.68, -1.09)	-1.53 (-1.83, -1.23)	-3.03 (-3.26, -2.79)	-3.16 (-3.39, -2.93)	-1.10 (-1.30, -0.90)	-1.08 (-1.27, -0.88)
Bolivarian Republic of Venezuela	-2.94 (-3.10, -2.77)	-2.91 (-3.07, -2.76)	-3.87 (-4.11, -3.62)	-3.79 (-4.03, -3.56)	-0.70 (-0.86, -0.54)	-0.71 (-0.87, -0.56)
Bosnia and Herzegovina	0.12 (-0.05, 0.29)	0.11 (-0.08, 0.31)	-1.27 (-1.46, -1.09)	-1.43 (-1.63, -1.24)	2.05 (1.64, 2.47)	1.88 (1.47, 2.30)
Brunei Darussalam	-1.90 (-2.12, -1.67)	-2.07 (-2.29, -1.85)	-3.98 (-4.33, -3.63)	-4.21 (-4.59, -3.84)	-1.91 (-2.34, -1.48)	-1.97 (-2.43, -1.5)
Burkina Faso	2.38 (2.13, 2.63)	2.29 (2.05, 2.54)	-0.76 (-0.96, -0.56)	-0.91 (-1.13, -0.70)	0.38 (0.24, 0.51)	0.23 (0.08, 0.38)
Canada	-1.18 (-1.32, -1.04)	-1.36 (-1.5, -1.22)	-3.80 (-3.90, -3.70)	-4.00 (-4.09, -3.91)	-2.34 (-2.40, -2.28)	-2.34 (-2.39, -2.28)
Central African Republic	-1.95 (-2.14, -1.77)	-1.96 (-2.14, -1.77)	-1.99 (-2.17, -1.81)	-1.97 (-2.15, -1.79)	-1.01 (-1.13, -0.88)	-0.98 (-1.10, -0.85)
Commonwealth of Dominica	-0.27 (-0.53, -0.01)	-0.06 (-0.32, 0.19)	-1.66 (-1.9, -1.42)	-1.52 (-1.75, -1.28)	0.58 (0.49, 0.67)	0.68 (0.58, 0.78)
Commonwealth of the Bahamas	-0.23 (-0.52, 0.05)	-0.42 (-0.71, -0.13)	-1.67 (-1.79, -1.56)	-1.85 (-1.97, -1.73)	0.73 (0.61, 0.85)	0.55 (0.43, 0.67)
Cook Islands	-1.27 (-1.40, -1.13)	-1.19 (-1.30, -1.08)	-2.65 (-2.81, -2.5)	-2.55 (-2.73, -2.37)	-1.79 (-1.96, -1.62)	-1.73 (-1.90, -1.57)

Cote dIvoire	-0.49 (-0.86, -0.12)	-0.53 (-0.91, -0.15)	-1.78 (-2.17, -1.39)	-1.83 (-2.23, -1.42)	-1.01 (-1.30, -0.72)	-1.02 (-1.32, -0.72)
Czech Republic	-0.35 (-0.46, -0.24)	-0.55 (-0.69, -0.41)	-4.48 (-4.58, -4.38)	-4.46 (-4.53, -4.38)	-2.65 (-2.85, -2.45)	-2.74 (-2.93, -2.54)
Democratic People's Republic of Korea	-0.52 (-0.62, -0.42)	-0.51 (-0.63, -0.38)	-0.99 (-1.13, -0.85)	-0.97 (-1.13, -0.81)	-0.33 (-0.41, -0.25)	-0.27 (-0.35, -0.19)
Democratic Republic of Sao Tome and Principe	3.71 (3.48, 3.94)	3.61 (3.37, 3.84)	-0.24 (-0.54, 0.07)	-0.36 (-0.67, -0.04)	1.15 (0.99, 1.31)	1.06 (0.89, 1.22)
Democratic Republic of the Congo	-1.46 (-1.76, -1.17)	-1.41 (-1.69, -1.13)	-1.38 (-1.54, -1.21)	-1.27 (-1.42, -1.12)	-0.61 (-1.01, -0.21)	-0.49 (-0.87, -0.11)
Democratic Republic of Timor-Leste	-0.68 (-0.89, -0.47)	-0.73 (-0.99, -0.47)	-1.23 (-1.45, -1.01)	-1.28 (-1.56, -1.01)	0.35 (0.19, 0.52)	0.37 (0.16, 0.58)
Democratic Socialist Republic of Sri Lanka	-1.97 (-2.18, -1.76)	-1.95 (-2.18, -1.72)	-4.32 (-4.52, -4.12)	-4.26 (-4.48, -4.03)	-1.39 (-1.55, -1.23)	-1.31 (-1.46, -1.15)
Dominican Republic	0.19 (-0.03, 0.42)	0.30 (0.13, 0.48)	-1.10 (-1.42, -0.78)	-0.98 (-1.24, -0.72)	0.51 (0.17, 0.84)	0.61 (0.35, 0.88)
Eastern Republic of Uruguay	-2.05 (-2.31, -1.78)	-2.23 (-2.51, -1.95)	-1.86 (-2.00, -1.72)	-1.93 (-2.07, -1.79)	-0.28 (-0.42, -0.13)	-0.38 (-0.53, -0.23)
Federal Democratic Republic of Ethiopia	-2.78 (-3.26, -2.29)	-3.02 (-3.50, -2.54)	-3.59 (-3.92, -3.26)	-3.98 (-4.31, -3.64)	-1.51 (-1.95, -1.07)	-1.97 (-2.41, -1.52)
Federal Democratic Republic of Nepal	-2.03 (-2.34, -1.73)	-2.36 (-2.67, -2.05)	-2.61 (-2.81, -2.41)	-2.99 (-3.21, -2.78)	-1.55 (-1.81, -1.29)	-1.84 (-2.10, -1.58)
Federal Republic of Germany	-0.49 (-0.67, -0.32)	-0.93 (-1.13, -0.73)	-3.88 (-4.01, -3.74)	-3.68 (-3.80, -3.57)	-2.41 (-2.49, -2.32)	-2.25 (-2.33, -2.17)
Federal Republic of Nigeria	1.08 (0.84, 1.32)	1.07 (0.84, 1.30)	-2.80 (-3.01, -2.59)	-2.84 (-3.07, -2.61)	-1.10 (-1.22, -0.98)	-1.11 (-1.25, -0.96)
Federal Republic of Somalia	-2.14 (-2.27, -2.02)	-2.21 (-2.34, -2.07)	-1.48 (-1.54, -1.41)	-1.47 (-1.54, -1.40)	-0.43 (-0.51, -0.35)	-0.49 (-0.56, -0.42)

Federated States of Micronesia	-0.69 (-0.73, -0.65)	-0.66 (-0.69, -0.62)	-1.21 (-1.32, -1.10)	-1.16 (-1.27, -1.05)	-0.20 (-0.24, -0.17)	-0.15 (-0.19, -0.11)
Federative Republic of Brazil	-3.03 (-3.13, -2.93)	-3.15 (-3.28, -3.01)	-4.64 (-4.75, -4.52)	-4.73 (-4.85, -4.60)	-0.98 (-1.06, -0.89)	-1.01 (-1.12, -0.90)
French Republic	-3.54 (-3.66, -3.42)	-3.80 (-3.93, -3.68)	-3.59 (-3.67, -3.50)	-3.14 (-3.25, -3.03)	-1.77 (-1.82, -1.72)	-1.46 (-1.53, -1.40)
Gabonese Republic	-0.14 (-0.24, -0.04)	-0.20 (-0.31, -0.10)	-1.30 (-1.35, -1.25)	-1.33 (-1.39, -1.27)	0.25 (0.19, 0.30)	0.22 (0.18, 0.27)
Georgia	-0.88 (-1.43, -0.32)	-1.04 (-1.58, -0.49)	0.60 (0.06, 1.15)	0.17 (-0.35, 0.69)	2.81 (2.33, 3.30)	2.32 (1.88, 2.77)
Grand Duchy of Luxembourg	-1.81 (-2.00, -1.63)	-2.15 (-2.35, -1.95)	-4.25 (-4.32, -4.18)	-4.63 (-4.71, -4.55)	-2.66 (-2.89, -2.42)	-2.87 (-3.10, -2.64)
Greenland	-2.09 (-2.18, -2.00)	-2.26 (-2.36, -2.15)	-3.66 (-3.75, -3.58)	-3.77 (-3.84, -3.69)	-2.06 (-2.15, -1.97)	-2.11 (-2.20, -2.01)
Grenada	-1.39 (-2.00, -0.78)	-1.30 (-1.82, -0.78)	-2.40 (-2.85, -1.95)	-2.43 (-2.75, -2.10)	0.54 (0.19, 0.89)	0.44 (0.20, 0.68)
Guam	0.06 (-0.30, 0.41)	0.41 (0.06, 0.76)	-1.07 (-1.41, -0.74)	-0.68 (-0.98, -0.38)	-0.44 (-0.75, -0.13)	-0.10 (-0.39, 0.19)
Hashemite Kingdom of Jordan	-0.88 (-1.04, -0.71)	-1.13 (-1.32, -0.95)	-2.37 (-2.51, -2.23)	-2.64 (-2.79, -2.50)	-0.60 (-0.75, -0.44)	-0.70 (-0.85, -0.55)
Hellenic Republic	-1.28 (-1.46, -1.09)	-0.95 (-1.10, -0.80)	-3.09 (-3.26, -2.92)	-2.98 (-3.13, -2.84)	-0.35 (-0.49, -0.22)	-0.16 (-0.26, -0.05)
Hungary	-1.81 (-2.16, -1.45)	-2.23 (-2.64, -1.82)	-4.05 (-4.32, -3.77)	-4.04 (-4.33, -3.75)	-0.76 (-1.05, -0.47)	-0.77 (-1.08, -0.46)
Independent State of Papua New Guinea	-1.42 (-1.54, -1.29)	-1.45 (-1.57, -1.32)	-1.65 (-1.76, -1.55)	-1.65 (-1.77, -1.54)	-1.05 (-1.18, -0.92)	-1.08 (-1.21, -0.94)
Independent State of Samoa	-1.02 (-1.14, -0.91)	-1.02 (-1.14, -0.91)	-1.54 (-1.68, -1.40)	-1.52 (-1.66, -1.37)	-0.88 (-1.00, -0.76)	-0.82 (-0.94, -0.69)
Ireland	-2.54 (-2.68, -2.39)	-2.62 (-2.76, -2.48)	-5.47 (-5.61, -5.32)	-5.57 (-5.74, -5.41)	-3.45 (-3.54, -3.36)	-3.37 (-3.46, -3.28)
Islamic Republic of Afghanistan	-0.14 (-0.54, 0.26)	-0.28 (-0.69, 0.14)	-0.08 (-0.56, 0.41)	-0.12 (-0.61, 0.37)	0.79 (0.62, 0.95)	0.87 (0.71, 1.04)
Islamic Republic of Iran	-0.53 (-0.69, -0.37)	-0.85 (-1.01, -0.69)	-1.50 (-1.64, -1.36)	-1.73 (-1.88, -1.58)	0.80 (0.59, 1.01)	0.67 (0.45, 0.88)
Islamic Republic of Mauritania	1.52 (1.14, 1.90)	1.36 (0.97, 1.75)	-1.81 (-2.11, -1.52)	-2.05 (-2.35, -1.75)	-0.53 (-0.78, -0.28)	-0.74 (-0.99, -0.49)

Islamic Republic of Pakistan	-1.50 (-1.80, -1.19)	-1.55 (-1.87, -1.22)	-2.43 (-2.80, -2.05)	-2.38 (-2.78, -1.99)	-0.93 (-1.26, -0.60)	-0.96 (-1.31, -0.62)
Jamaica	-0.71 (-1.21, -0.21)	-0.77 (-1.31, -0.23)	-2.72 (-3.10, -2.35)	-2.77 (-3.17, -2.36)	0.55 (0.10, 0.99)	0.53 (0.04, 1.03)
Japan	-2.02 (-2.19, -1.85)	-2.36 (-2.59, -2.13)	-4.71 (-4.77, -4.64)	-5.03 (-5.11, -4.95)	-2.19 (-2.28, -2.10)	-2.22 (-2.31, -2.13)
Kingdom of Bahrain	-2.96 (-3.31, -2.6)	-3.31 (-3.64, -2.97)	-3.33 (-3.63, -3.04)	-3.65 (-3.90, -3.40)	-1.28 (-1.52, -1.04)	-1.46 (-1.64, -1.27)
Kingdom of Belgium	-0.57 (-0.79, -0.35)	-0.76 (-1.03, -0.48)	-4.04 (-4.17, -3.91)	-3.89 (-4.01, -3.77)	-2.70 (-2.85, -2.55)	-2.58 (-2.74, -2.41)
Kingdom of Bhutan	-1.01 (-1.25, -0.78)	-1.46 (-1.7, -1.21)	-1.47 (-1.62, -1.31)	-1.93 (-2.10, -1.76)	-0.18 (-0.32, -0.04)	-0.49 (-0.64, -0.35)
Kingdom of Cambodia	-1.57 (-1.68, -1.45)	-1.78 (-1.91, -1.65)	-2.16 (-2.28, -2.04)	-2.41 (-2.54, -2.27)	0.04 (-0.04, 0.12)	-0.13 (-0.22, -0.04)
Kingdom of Denmark	-1.18 (-1.35, -1.01)	-1.48 (-1.64, -1.32)	-3.59 (-3.88, -3.30)	-3.67 (-3.96, -3.38)	-2.77 (-3.10, -2.45)	-2.91 (-3.20, -2.63)
Kingdom of Eswatini	-0.99 (-1.57, -0.41)	-0.94 (-1.58, -0.30)	-1.40 (-1.90, -0.90)	-1.32 (-1.89, -0.76)	0.11 (-0.33, 0.56)	0.22 (-0.28, 0.72)
Kingdom of Lesotho	2.05 (1.72, 2.38)	2.36 (1.99, 2.74)	1.87 (1.54, 2.20)	2.28 (1.90, 2.66)	3.63 (3.30, 3.96)	3.99 (3.61, 4.36)
Kingdom of Morocco	-0.46 (-0.70, -0.21)	-0.51 (-0.76, -0.26)	-2.02 (-2.19, -1.84)	-2.09 (-2.27, -1.91)	0.42 (0.19, 0.64)	0.30 (0.08, 0.51)
Kingdom of Norway	-1.65 (-1.89, -1.40)	-1.77 (-1.99, -1.55)	-6.13 (-6.33, -5.94)	-6.16 (-6.32, -5.99)	-3.79 (-4.04, -3.54)	-3.76 (-3.99, -3.53)
Kingdom of Saudi Arabia	-0.63 (-0.78, -0.48)	-0.75 (-0.90, -0.60)	-1.98 (-2.21, -1.75)	-1.94 (-2.17, -1.71)	2.13 (1.74, 2.53)	2.16 (1.79, 2.54)
Kingdom of Spain	-2.60 (-2.74, -2.47)	-2.90 (-3.07, -2.73)	-4.18 (-4.25, -4.12)	-4.20 (-4.30, -4.10)	-1.11 (-1.36, -0.86)	-1.09 (-1.38, -0.81)
Kingdom of Sweden	-0.33 (-0.48, -0.17)	-0.44 (-0.61, -0.28)	-4.08 (-4.29, -3.88)	-4.15 (-4.33, -3.96)	-1.51 (-1.71, -1.30)	-1.63 (-1.83, -1.43)
Kingdom of Thailand	-1.91 (-2.07, -1.76)	-1.64 (-1.79, -1.50)	-3.26 (-3.45, -3.06)	-3.00 (-3.18, -2.81)	-0.75 (-0.88, -0.63)	-0.53 (-0.64, -0.41)
Kingdom of the Netherlands	-0.36 (-0.63, -0.08)	-0.69 (-0.99, -0.38)	-5.03 (-5.16, -4.90)	-5.21 (-5.34, -5.08)	-1.26 (-1.46, -1.07)	-1.40 (-1.59, -1.21)
Kingdom of Tonga	-0.63 (-0.88, -0.37)	-0.60 (-0.83, -0.37)	-1.51 (-1.67, -1.34)	-1.43 (-1.57, -1.29)	-0.58 (-0.75, -0.40)	-0.55 (-0.70, -0.40)
Kyrgyz Republic	-2.86 (-3.02, -2.70)	-3.37 (-3.52, -3.22)	-1.25 (-1.59, -0.91)	-1.65 (-1.94, -1.36)	-0.21 (-0.46, 0.03)	-0.55 (-0.77, -0.34)
Lao People's Democratic Republic	-1.90 (-20, -1.81)	-2.18 (-2.27, -2.08)	-2.47 (-2.56, -2.39)	-2.77 (-2.86, -2.68)	0.23 (0.18, 0.27)	-0.01 (-0.05, 0.04)
Lebanese Republic	-0.25 (-0.46, -0.04)	-0.56 (-0.76, -0.35)	-1.19 (-1.43, -0.94)	-1.53 (-1.75, -1.31)	0.35 (0.03, 0.67)	0.14 (-0.15, 0.44)
Malaysia	-0.89 (-1.08, -0.70)	-0.90 (-1.06, -0.73)	-2.44 (-2.65, -2.23)	-2.42 (-2.59, -2.25)	-0.79 (-0.98, -0.60)	-0.74 (-0.91, -0.56)

Mongolia	-0.72 (-0.85, -0.58)	-0.71 (-0.85, -0.56)	-0.80 (-0.90, -0.69)	-0.83 (-0.94, -0.72)	0.62 (0.49, 0.75)	0.53 (0.39, 0.67)
Montenegro	0.38 (0.26, 0.51)	0.14 (-0.03, 0.30)	-1.06 (-1.36, -0.75)	-1.31 (-1.67, -0.95)	0.34 (0.13, 0.54)	0.16 (-0.08, 0.41)
New Zealand	-2.51 (-2.70, -2.32)	-2.84 (-3.01, -2.67)	-3.60 (-3.91, -3.29)	-3.72 (-4.02, -3.42)	-2.79 (-2.95, -2.64)	-3.14 (-3.33, -2.95)
North Macedonia	-0.23 (-0.54, 0.09)	-0.38 (-0.68, -0.08)	-2.15 (-2.43, -1.86)	-2.37 (-2.62, -2.12)	0.21 (-0.12, 0.53)	0.04 (-0.25, 0.34)
Northern Mariana Islands	2.65 (2.05, 3.24)	2.66 (2.07, 3.26)	-1.45 (-1.71, -1.19)	-1.42 (-1.71, -1.13)	-0.51 (-0.7, -0.31)	-0.51 (-0.72, -0.29)
Palestine	-1.88 (-2.13, -1.62)	-1.84 (-2.07, -1.62)	-2.81 (-3.13, -2.49)	-2.77 (-3.05, -2.49)	-0.90 (-1.08, -0.72)	-0.83 (-0.98, -0.68)
People's Democratic Republic of Algeria	-0.31 (-0.40, -0.23)	-0.50 (-0.57, -0.44)	-2.05 (-2.24, -1.85)	-2.31 (-2.45, -2.16)	-0.50 (-0.68, -0.32)	-0.66 (-0.78, -0.54)
People's Republic of Bangladesh	-1.87 (-2.05, -1.69)	-2.04 (-2.18, -1.90)	-2.54 (-2.75, -2.32)	-2.75 (-2.91, -2.58)	-1.26 (-1.42, -1.09)	-1.38 (-1.50, -1.26)
People's Republic of China	-1.62 (-1.79, -1.46)	-2.03 (-2.18, -1.89)	-2.17 (-2.29, -2.05)	-2.58 (-2.68, -2.48)	-0.37 (-0.48, -0.26)	-0.46 (-0.60, -0.31)
Plurinational State of Bolivia	-0.73 (-0.97, -0.49)	-0.98 (-1.23, -0.73)	-1.62 (-1.84, -1.39)	-1.82 (-2.05, -1.58)	0.23 (0.05, 0.42)	0.06 (-0.13, 0.25)
Portuguese Republic	-1.78 (-1.96, -1.60)	-1.48 (-1.73, -1.23)	-4.17 (-4.29, -4.05)	-3.91 (-4.06, -3.76)	-1.13 (-1.36, -0.90)	-0.85 (-1.09, -0.61)
Principality of Andorra	-2.09 (-2.39, -1.80)	-2.20 (-2.46, -1.94)	-3.09 (-3.37, -2.81)	-3.14 (-3.39, -2.88)	-1.91 (-2.17, -1.65)	-1.88 (-2.11, -1.65)
Principality of Monaco	-0.18 (-0.40, 0.05)	-0.26 (-0.49, -0.03)	-2.52 (-2.63, -2.42)	-2.60 (-2.71, -2.50)	-0.46 (-0.56, -0.36)	-0.46 (-0.56, -0.37)
Puerto Rico	-3.72 (-3.92, -3.52)	-3.50 (-3.70, -3.29)	-4.34 (-4.49, -4.19)	-4.08 (-4.23, -3.92)	-0.45 (-0.67, -0.22)	-0.22 (-0.41, -0.04)
Republic of Albania	-0.36 (-0.61, -0.11)	-0.42 (-0.66, -0.17)	-1.32 (-1.55, -1.09)	-1.42 (-1.65, -1.19)	0.65 (0.40, 0.90)	0.66 (0.41, 0.91)
Republic of Angola	-1.57 (-1.85, -1.29)	-1.66 (-1.95, -1.37)	-2.07 (-2.31, -1.83)	-2.12 (-2.37, -1.87)	-0.12 (-0.42, 0.18)	-0.12 (-0.43, 0.19)
Republic of Armenia	-3.09 (-3.44, -2.75)	-3.26 (-3.64, -2.88)	-2.01 (-2.24, -1.78)	-2.57 (-2.79, -2.35)	0.33 (0.02, 0.63)	-0.21 (-0.51, 0.09)
Republic of Austria	-0.10 (-0.24, 0.03)	-0.41 (-0.57, -0.25)	-3.96 (-4.10, -3.82)	-3.84 (-3.99, -3.68)	-2.41 (-2.54, -2.28)	-2.40 (-2.52, -2.28)
Republic of Azerbaijan	-0.39 (-0.64, -0.15)	-0.90 (-1.11, -0.68)	-1.17 (-1.40, -0.95)	-1.80 (-2.00, -1.61)	0.16 (-0.15, 0.48)	-0.36 (-0.66, -0.07)
Republic of Belarus	-0.40 (-0.56, -0.24)	-0.44 (-0.62, -0.27)	-3.56 (-3.75, -3.37)	-3.77 (-3.97, -3.56)	-0.28 (-0.51, -0.04)	-0.53 (-0.77, -0.28)
Republic of Benin	0.68 (0.50, 0.86)	0.65 (0.47, 0.83)	-2.57 (-2.65, -2.50)	-2.65 (-2.73, -2.57)	-1.43 (-1.54, -1.31)	-1.50 (-1.62, -1.38)
Republic of Botswana	-1.66 (-1.97, -1.35)	-1.89 (-2.26, -1.52)	-2.74 (-2.98, -2.50)	-2.94 (-3.24, -2.65)	-0.39 (-0.59, -0.19)	-0.55 (-0.80, -0.30)

Republic of Bulgaria	-0.76 (-0.98, -0.55)	-0.76 (-0.98, -0.53)	-2.27 (-2.42, -2.12)	-2.28 (-2.42, -2.15)	0.59 (0.42, 0.75)	0.45 (0.30, 0.60)
Republic of Burundi	-3.91 (-4.40, -3.42)	-4.01 (-4.50, -3.51)	-3.24 (-3.67, -2.80)	-3.32 (-3.77, -2.88)	-1.55 (-1.98, -1.11)	-1.70 (-2.15, -1.26)
Republic of Cabo Verde	0.67 (0.13, 1.22)	0.73 (0.28, 1.18)	-2.28 (-2.88, -1.68)	-2.33 (-2.84, -1.82)	1.27 (0.77, 1.77)	1.20 (0.76, 1.64)
Republic of Cameroon	2.01 (1.76, 2.26)	1.97 (1.71, 2.24)	-1.34 (-1.55, -1.14)	-1.49 (-1.72, -1.26)	-0.25 (-0.39, -0.12)	-0.39 (-0.54, -0.24)
Republic of Chad	2.52 (2.28, 2.76)	2.57 (2.32, 2.83)	-0.39 (-0.59, -0.20)	-0.40 (-0.62, -0.19)	0.55 (0.38, 0.72)	0.55 (0.36, 0.74)
Republic of Chile	-4.54 (-4.8, -4.27)	-4.65 (-4.88, -4.42)	-3.44 (-3.58, -3.30)	-3.45 (-3.56, -3.33)	0.00 (-0.11, 0.11)	0.06 (-0.08, 0.19)
Republic of Colombia	-5.39 (-5.64, -5.14)	-5.53 (-5.77, -5.28)	-5.02 (-5.21, -4.83)	-4.93 (-5.11, -4.75)	-1.18 (-1.32, -1.03)	-1.16 (-1.31, -1.01)
Republic of Costa Rica	-3.04 (-3.25, -2.84)	-3.21 (-3.40, -3.03)	-4.37 (-4.59, -4.16)	-4.34 (-4.57, -4.12)	1.06 (0.83, 1.29)	1.15 (0.93, 1.37)
Republic of Croatia	-1.51 (-1.68, -1.34)	-1.69 (-1.88, -1.49)	-3.66 (-3.79, -3.54)	-3.79 (-3.91, -3.68)	0.18 (-0.01, 0.38)	0.09 (-0.11, 0.29)
Republic of Cuba	0.62 (0.50, 0.74)	0.98 (0.86, 1.10)	-1.97 (-2.08, -1.86)	-1.93 (-2.05, -1.80)	-0.46 (-0.55, -0.36)	-0.51 (-0.60, -0.41)
Republic of Cyprus	0.25 (0.03, 0.47)	0.56 (0.31, 0.80)	-2.23 (-2.37, -2.09)	-2.06 (-2.20, -1.93)	-1.23 (-1.37, -1.10)	-0.97 (-1.13, -0.81)
Republic of Djibouti	-0.77 (-0.85, -0.69)	-0.92 (-1.03, -0.81)	-0.63 (-0.76, -0.50)	-0.79 (-0.95, -0.63)	1.46 (1.30, 1.61)	1.18 (1.02, 1.34)
Republic of Ecuador	-2.75 (-3.07, -2.44)	-2.95 (-3.26, -2.64)	-3.01 (-3.39, -2.63)	-3.05 (-3.40, -2.70)	0.45 (0.11, 0.79)	0.44 (0.10, 0.78)
Republic of El Salvador	-0.12 (-0.34, 0.11)	-0.02 (-0.25, 0.20)	-0.42 (-0.69, -0.16)	-0.32 (-0.58, -0.07)	1.83 (1.62, 2.05)	2 .00(1.79, 2.21)
Republic of Equatorial Guinea	-1.11 (-1.70, -0.52)	-1.40 (-2.03, -0.77)	-3.46 (-4.05, -2.86)	-3.70 (-4.32, -3.07)	0.96 (0.64, 1.29)	0.75 (0.40, 1.10)
Republic of Estonia	-1.04 (-1.23, -0.85)	-1.36 (-1.55, -1.16)	-3.53 (-3.72, -3.33)	-3.85 (-4.06, -3.64)	-0.62 (-0.80, -0.45)	-0.95 (-1.13, -0.77)
Republic of Fiji	-0.73 (-0.85, -0.61)	-0.69 (-0.83, -0.55)	-2.03 (-2.24, -1.82)	-2.05 (-2.28, -1.83)	-0.70 (-0.93, -0.47)	-0.78 (-1.02, -0.53)
Republic of Finland	-1.02 (-1.11, -0.93)	-1.13 (-1.23, -1.04)	-4.44 (-4.55, -4.33)	-4.53 (-4.64, -4.41)	-1.33 (-1.39, -1.27)	-1.44 (-1.5, -1.38)
Republic of Ghana	1.71 (1.48, 1.95)	1.64 (1.40, 1.87)	-1.14 (-1.27, -1.02)	-1.12 (-1.23, -1.01)	1.36 (1.20, 1.53)	1.33 (1.16, 1.50)
Republic of Guatemala	-2.14 (-2.50, -1.78)	-2.03 (-2.40, -1.66)	-1.84 (-2.57, -1.10)	-1.67 (-2.38, -0.94)	0.64 (0.25, 1.04)	0.91 (0.53, 1.29)
Republic of Guinea	0.74 (0.55, 0.93)	0.73 (0.52, 0.95)	-0.44 (-0.63, -0.26)	-0.44 (-0.66, -0.22)	0.37 (0.25, 0.49)	0.41 (0.25, 0.56)
Republic of Guinea-Bissau	3.63 (3.14, 4.12)	3.69 (3.20, 4.18)	0.30 (-0.08, 0.67)	0.31 (-0.06, 0.69)	0.79 (0.52, 1.06)	0.84 (0.57, 1.11)
Republic of Guyana	-0.01 (-0.19, 0.17)	0.16 (-0.03, 0.35)	-2.06 (-2.35, -1.77)	-1.91 (-2.20, -1.62)	0.51 (0.24, 0.78)	0.62 (0.35, 0.89)
Republic of Haiti	-1.90 (-2.09, -1.72)	-2.14 (-2.34, -1.94)	-2.34 (-2.51, -2.17)	-2.65 (-2.84, -2.45)	-0.82 (-0.9, -0.74)	-1.09 (-1.18, -0.99)

Republic of Honduras	1.46 (1.24, 1.67)	1.20 (1.00, 1.4)	0.17 (-0.03, 0.37)	-0.14 (-0.32, 0.03)	1.24 (1.08, 1.39)	0.92 (0.79, 1.04)
Republic of Iceland	-1.64 (-1.80, -1.48)	-1.77 (-1.93, -1.61)	-5.11 (-5.18, -5.04)	-5.32 (-5.39, -5.25)	-2.53 (-2.68, -2.38)	-2.6 (-2.73, -2.46)
Republic of India	-1.58 (-1.67, -1.49)	-1.77 (-1.87, -1.68)	-1.90 (-2.05, -1.75)	-2.21 (-2.34, -2.08)	-1.02 (-1.11, -0.94)	-1.18 (-1.27, -1.09)
Republic of Indonesia	0.50 (0.34, 0.65)	0.35 (0.22, 0.49)	-0.43 (-0.63, -0.23)	-0.63 (-0.81, -0.46)	1.39 (1.16, 1.62)	1.27 (1.06, 1.47)
Republic of Iraq	-0.64 (-0.85, -0.43)	-0.95 (-1.13, -0.78)	-1.62 (-1.80, -1.44)	-1.99 (-2.13, -1.85)	0.07 (-0.16, 0.29)	-0.21 (-0.39, -0.02)
Republic of Italy	-3.19 (-3.25, -3.13)	-3.40 (-3.47, -3.34)	-4.30 (-4.35, -4.25)	-4.33 (-4.38, -4.29)	-2.14 (-2.29, -2.00)	-2.15 (-2.31, -2.00)
Republic of Kazakhstan	-4.81 (-5.11, -4.51)	-5.01 (-5.28, -4.73)	-3.79 (-4.07, -3.51)	-3.97 (-4.23, -3.71)	-1.09 (-1.35, -0.83)	-1.29 (-1.52, -1.05)
Republic of Kenya	-0.17 (-0.38, 0.04)	-0.16 (-0.41, 0.09)	-1.18 (-1.43, -0.93)	-1.14 (-1.44, -0.84)	0.43 (0.26, 0.59)	0.42 (0.22, 0.61)
Republic of Kiribati	-0.47 (-0.66, -0.28)	-0.46 (-0.64, -0.29)	-0.58 (-0.75, -0.40)	-0.56 (-0.72, -0.40)	-0.35 (-0.56, -0.13)	-0.32 (-0.52, -0.13)
Republic of Korea	-4.07 (-4.26, -3.87)	-4.68 (-4.87, -4.49)	-6.01 (-6.19, -5.82)	-6.60 (-6.75, -6.44)	-1.31 (-1.51, -1.12)	-1.61 (-1.83, -1.40)
Republic of Latvia	-0.33 (-0.54, -0.12)	-0.52 (-0.74, -0.30)	-2.97 (-3.14, -2.80)	-3.17 (-3.35, -2.99)	-0.13 (-0.31, 0.05)	-0.32 (-0.49, -0.15)
Republic of Liberia	2.34 (2.02, 2.67)	2.44 (2.10, 2.77)	-1.09 (-1.22, -0.95)	-1.05 (-1.19, -0.90)	-0.28 (-0.48, -0.08)	-0.22 (-0.43, 0.00)
Republic of Lithuania	0.28 (0.09, 0.48)	0.20 (-0.04, 0.43)	-2.86 (-3.03, -2.68)	-2.91 (-3.10, -2.72)	0.14 (-0.07, 0.35)	0.02 (-0.20, 0.24)
Republic of Madagascar	-3.14 (-3.41, -2.87)	-3.23 (-3.48, -2.98)	-3.07 (-3.30, -2.85)	-3.09 (-3.29, -2.89)	-1.38 (-1.68, -1.09)	-1.52 (-1.79, -1.24)
Republic of Malawi	0.91 (0.43, 1.39)	0.98 (0.47, 1.50)	-1.12 (-1.43, -0.81)	-1.02 (-1.37, -0.66)	0.19 (-0.03, 0.41)	0.29 (0.02, 0.55)
Republic of Maldives	-3.32 (-3.58, -3.07)	-3.79 (-4.10, -3.48)	-4.31 (-4.62, -4.00)	-4.83 (-5.20, -4.46)	-2.15 (-2.37, -1.93)	-2.52 (-2.78, -2.26)
Republic of Mali	1.74 (1.55, 1.93)	1.62 (1.43, 1.82)	0.52 (0.36, 0.69)	0.36 (0.19, 0.54)	1.45 (1.31, 1.60)	1.31 (1.15, 1.46)
Republic of Malta	-1.57 (-1.69, -1.44)	-1.40 (-1.51, -1.28)	-4.04 (-4.24, -3.84)	-3.97 (-4.17, -3.78)	-1.93 (-2.07, -1.79)	-1.80 (-1.95, -1.65)
Republic of Mauritius	-0.57 (-1.11, -0.02)	-0.62 (-1.18, -0.05)	-2.44 (-2.95, -1.93)	-2.48 (-3.00, -1.95)	1.23 (0.91, 1.54)	1.20 (0.87, 1.53)
Republic of Moldova	-0.41 (-0.85, 0.04)	-0.45 (-0.89, -0.01)	-1.90 (-2.29, -1.51)	-2.03 (-2.40, -1.66)	1.24 (0.82, 1.66)	1.11 (0.72, 1.49)
Republic of Mozambique	0.80 (0.58, 1.02)	1.05 (0.79, 1.30)	-0.02 (-0.24, 0.21)	0.23 (-0.03, 0.50)	0.95 (0.77, 1.14)	1.18 (0.97, 1.40)
Republic of Namibia	-1.04 (-1.41, -0.67)	-1.03 (-1.43, -0.63)	-1.86 (-2.23, -1.50)	-1.91 (-2.31, -1.52)	-0.59 (-0.88, -0.29)	-0.59 (-0.90, -0.27)
Republic of Nauru	-1.40 (-1.58, -1.23)	-1.31 (-1.50, -1.12)	-1.55 (-1.86, -1.24)	-1.42 (-1.75, -1.10)	-0.96 (-1.03, -0.88)	-0.84 (-0.93, -0.76)
Republic of Nicaragua	-0.83 (-1.04, -0.62)	-0.87 (-1.06, -0.67)	-1.36 (-1.57, -1.14)	-1.37 (-1.57, -1.18)	1.16 (0.89, 1.42)	1.16 (0.91, 1.41)
Republic of Niue	-0.32 (-0.45, -0.19)	-0.46 (-0.58, -0.33)	-1.42 (-1.52, -1.32)	-1.57 (-1.68, -1.46)	-0.10 (-0.16, -0.03)	-0.21 (-0.28, -0.14)
Republic of Palau	-0.95 (-1.06, -0.85)	-0.94 (-1.06, -0.82)	-1.60 (-1.67, -1.54)	-1.51 (-1.58, -1.44)	-0.80 (-0.83, -0.77)	-0.97 (-1.01, -0.94)

Republic of Panama	-2.79 (-2.91, -2.66)	-2.79 (-2.92, -2.66)	-2.90 (-3.07, -2.72)	-2.90 (-3.09, -2.71)	-0.32 (-0.44, -0.21)	-0.19 (-0.32, -0.06)
Republic of Paraguay	-0.60 (-0.79, -0.40)	-0.66 (-0.86, -0.46)	-2.43 (-2.74, -2.12)	-2.46 (-2.77, -2.15)	0.79 (0.57, 1.02)	0.78 (0.55, 1.00)
Republic of Peru	-1.56 (-1.89, -1.22)	-1.68 (-2.03, -1.34)	-1.77 (-2.07, -1.46)	-1.86 (-2.17, -1.55)	0.16 (-0.05, 0.37)	0.11 (-0.10, 0.33)
Republic of Poland	-1.51 (-1.70, -1.33)	-1.60 (-1.79, -1.41)	-3.89 (-4.05, -3.72)	-3.94 (-4.10, -3.79)	-0.23 (-0.39, -0.06)	-0.4 (-0.55, -0.24)
Republic of Rwanda	-2.46 (-2.83, -2.08)	-2.91 (-3.32, -2.50)	-2.76 (-3.09, -2.41)	-3.17 (-3.54, -2.79)	-0.63 (-0.88, -0.38)	-1.07 (-1.36, -0.79)
Republic of San Marino	-1.76 (-2.09, -1.42)	-1.88 (-2.18, -1.57)	-3.47 (-3.78, -3.17)	-3.57 (-3.85, -3.30)	-2.38 (-2.68, -2.07)	-2.35 (-2.63, -2.07)
Republic of Senegal	1.27 (0.97, 1.58)	1.18 (0.88, 1.48)	-1.95 (-2.15, -1.75)	-2.12 (-2.31, -1.93)	-0.74 (-0.91, -0.56)	-0.88 (-1.05, -0.71)
Republic of Serbia	-0.30 (-0.51, -0.08)	-0.31 (-0.55, -0.07)	-2.05 (-2.38, -1.72)	-2.10 (-2.45, -1.75)	-0.32 (-0.52, -0.12)	-0.31 (-0.52, -0.10)
Republic of Seychelles	-0.61 (-0.80, -0.42)	-0.80 (-0.98, -0.62)	-2.25 (-2.44, -2.07)	-2.45 (-2.62, -2.27)	0.31 (0.07, 0.55)	0.17 (-0.07, 0.41)
Republic of Sierra Leone	1.82 (1.51, 2.14)	1.91 (1.58, 2.24)	-1.17 (-1.35, -0.98)	-1.13 (-1.32, -0.93)	-0.42 (-0.57, -0.27)	-0.36 (-0.52, -0.21)
Republic of Singapore	-4.40 (-4.74, -4.06)	-4.59 (-4.90, -4.27)	-6.04 (-6.29, -5.79)	-6.27 (-6.52, -6.02)	-2.98 (-3.16, -2.79)	-3.00 (-3.19, -2.81)
Republic of Slovenia	-1.88 (-2.05, -1.72)	-2.11 (-2.28, -1.93)	-3.53 (-3.69, -3.36)	-3.80 (-3.97, -3.63)	-1.26 (-1.61, -0.90)	-1.46 (-1.80, -1.11)
Republic of South Africa	-2.77 (-3.27, -2.28)	-2.72 (-3.19, -2.25)	-2.81 (-3.07, -2.55)	-2.70 (-2.93, -2.48)	-0.85 (-1.02, -0.68)	-0.68 (-0.83, -0.53)
Republic of South Sudan	-1.73 (-1.95, -1.51)	-1.80 (-2.05, -1.55)	-1.57 (-1.77, -1.36)	-1.61 (-1.86, -1.36)	-0.07 (-0.2, 0.05)	-0.22 (-0.39, -0.05)
Republic of Sudan	0.08 (0.03, 0.13)	-0.21 (-0.27, -0.15)	-1.35 (-1.48, -1.22)	-1.65 (-1.79, -1.51)	-0.08 (-0.13, -0.04)	-0.29 (-0.34, -0.24)
Republic of Suriname	-0.57 (-0.8, -0.34)	-0.49 (-0.71, -0.26)	-2.30 (-2.56, -2.04)	-2.26 (-2.51, -2.00)	-0.05 (-0.30, 0.21)	-0.04 (-0.29, 0.20)
Republic of Tajikistan	-2.57 (-2.86, -2.27)	-3.04 (-3.31, -2.77)	-3.69 (-3.98, -3.39)	-4.11 (-4.36, -3.86)	-2.89 (-3.15, -2.62)	-3.24 (-3.49, -2.98)
Republic of the Congo	-0.93 (-1.19, -0.67)	-1.18 (-1.45, -0.91)	-1.51 (-1.72, -1.30)	-1.72 (-1.94, -1.50)	0.28 (0.02, 0.54)	0.11 (-0.16, 0.37)
Republic of the Gambia	-0.43 (-0.56, -0.29)	-0.49 (-0.64, -0.34)	-2.31 (-2.47, -2.15)	-2.40 (-2.58, -2.23)	-1.27 (-1.43, -1.11)	-1.34 (-1.52, -1.16)
Republic of the Marshall Islands	-0.05 (-0.18, 0.08)	-0.14 (-0.25, -0.03)	-0.77 (-0.88, -0.66)	-0.83 (-0.92, -0.74)	0.35 (0.26, 0.44)	0.31 (0.23, 0.39)
Republic of the Niger	1.67 (1.47, 1.86)	1.49 (1.28, 1.69)	-0.68 (-0.75, -0.60)	-0.96 (-1.04, -0.87)	-0.11 (-0.17, -0.06)	-0.38 (-0.45, -0.32)
Republic of the Philippines	-0.93 (-1.11, -0.74)	-0.99 (-1.19, -0.78)	-1.89 (-2.09, -1.69)	-1.92 (-2.13, -1.71)	0.01 (-0.15, 0.17)	-0.02 (-0.19, 0.14)
Republic of the Union of Myanmar	-3.68 (-3.85, -3.51)	-3.96 (-4.14, -3.79)	-4.57 (-4.76, -4.39)	-4.85 (-5.04, -4.66)	-1.76 (-1.87, -1.64)	-1.98 (-2.10, -1.86)

Republic of Trinidad and Tobago	-1.27 (-1.48, -1.06)	-1.15 (-1.36, -0.94)	-3.41 (-3.63, -3.19)	-3.22 (-3.44, -3.00)	-0.41 (-0.55, -0.27)	-0.31 (-0.46, -0.16)
Republic of Tunisia	-0.22 (-0.30, -0.15)	-0.27 (-0.34, -0.20)	-2.09 (-2.21, -1.98)	-2.07 (-2.17, -1.96)	-0.57 (-0.69, -0.46)	-0.44 (-0.54, -0.33)
Republic of Turkey	-1.84 (-2.08, -1.61)	-2.11 (-2.34, -1.88)	-3.67 (-3.95, -3.39)	-3.92 (-4.19, -3.64)	-1.72 (-2.01, -1.44)	-1.86 (-2.14, -1.57)
Republic of Uganda	-1.17 (-1.58, -0.75)	-1.19 (-1.63, -0.76)	-2.82 (-3.30, -2.34)	-2.86 (-3.36, -2.36)	-1.2 (-1.59, -0.80)	-1.19 (-1.61, -0.77)
Republic of Uzbekistan	-3.89 (-4.49, -3.29)	-4.29 (-4.88, -3.69)	-1.97 (-2.22, -1.72)	-2.29 (-2.55, -2.04)	0.25 (-0.06, 0.56)	0.01 (-0.34, 0.35)
Republic of Vanuatu	-1.45 (-1.57, -1.32)	-1.45 (-1.56, -1.33)	-2.07 (-2.18, -1.96)	-2.05 (-2.15, -1.94)	-1.15 (-1.24, -1.07)	-1.17 (-1.26, -1.08)
Republic of Yemen	-0.72 (-0.81, -0.63)	-1.09 (-1.18, -0.99)	-0.94 (-1.01, -0.88)	-1.33 (-1.41, -1.26)	0.13 (0.05, 0.20)	-0.12 (-0.19, -0.04)
Republic of Zambia	-1.77 (-2.09, -1.45)	-1.84 (-2.19, -1.49)	-2.15 (-2.54, -1.77)	-2.17 (-2.59, -1.76)	0.46 (0.32, 0.60)	0.45 (0.30, 0.61)
Republic of Zimbabwe	-0.21 (-0.56, 0.15)	-0.16 (-0.52, 0.21)	-0.03 (-0.42, 0.35)	0.15 (-0.27, 0.57)	0.35 (-0.04, 0.73)	0.48 (0.07, 0.90)
Romania	1.11 (0.78, 1.44)	1.12 (0.76, 1.49)	-1.92 (-2.14, -1.70)	-2.01 (-2.23, -1.79)	1.05 (0.80, 1.30)	0.90 (0.66, 1.15)
Russian Federation	-1.32 (-1.46, -1.18)	-1.45 (-1.61, -1.29)	-3.00 (-3.21, -2.79)	-3.32 (-3.52, -3.11)	0.10 (-0.22, 0.43)	-0.03 (-0.33, 0.28)
Saint Kitts and Nevis	-0.67 (-0.79, -0.55)	-0.82 (-0.95, -0.69)	-2.45 (-2.6, -2.29)	-2.68 (-2.82, -2.54)	0.36 (0.24, 0.48)	0.12 (0.01, 0.23)
Saint Lucia	-1.39 (-1.60, -1.19)	-1.35 (-1.54, -1.17)	-3.33 (-3.56, -3.09)	-3.29 (-3.49, -3.08)	-0.85 (-1.02, -0.68)	-0.80 (-0.94, -0.66)
Saint Vincent and the Grenadines	0.22 (-0.07, 0.51)	0.25 (-0.03, 0.54)	-1.85 (-2.11, -1.59)	-1.91 (-2.16, -1.65)	0.63 (0.47, 0.78)	0.61 (0.45, 0.76)
Slovak Republic	-1.31 (-1.41, -1.21)	-1.55 (-1.68, -1.43)	-3.10 (-3.24, -2.97)	-3.24 (-3.37, -3.10)	-0.59 (-0.67, -0.51)	-0.78 (-0.86, -0.70)
Socialist Republic of Viet Nam	0.25 (0.20, 0.29)	0.44 (0.37, 0.51)	-3.16 (-3.46, -2.86)	-3.00 (-3.26, -2.73)	0.93 (0.83, 1.03)	1.11 (1.04, 1.18)
Solomon Islands	-0.51 (-0.63, -0.40)	-0.41 (-0.53, -0.28)	-0.90 (-1.13, -0.67)	-0.77 (-1, -0.54)	-0.03 (-0.24, 0.19)	0.10 (-0.12, 0.32)
State of Eritrea	-2.71 (-2.94, -2.48)	-2.89 (-3.13, -2.66)	-2.55 (-2.71, -2.38)	-2.69 (-2.85, -2.52)	-0.42 (-0.54, -0.30)	-0.60 (-0.73, -0.48)
State of Israel	-2.18 (-2.35, -2.01)	-2.12 (-2.31, -1.92)	-3.89 (-4.05, -3.72)	-3.84 (-4.02, -3.66)	-3.08 (-3.38, -2.77)	-2.96 (-3.25, -2.67)
State of Kuwait	-2.00 (-2.86, -1.14)	-2.61 (-3.43, -1.77)	-1.64 (-2.10, -1.18)	-2.11 (-2.55, -1.67)	1.50 (1.00, 2.01)	1.20 (0.70, 1.70)
State of Libya	1.03 (0.85, 1.21)	0.93 (0.77, 1.09)	-0.32 (-0.54, -0.10)	-0.46 (-0.66, -0.26)	0.68 (0.49, 0.88)	0.61 (0.44, 0.78)
State of Qatar	-3.2 (-3.93, -2.47)	-3.24 (-3.92, -2.56)	-4.60 (-5.26, -3.94)	-4.62 (-5.21, -4.02)	-0.63 (-1.34, 0.09)	-0.56 (-1.21, 0.10)
Sultanate of Oman	-0.38 (-0.63, -0.14)	-0.77 (-0.96, -0.58)	-2.55 (-2.91, -2.19)	-3.04 (-3.33, -2.74)	-0.47 (-0.72, -0.22)	-0.78 (-1.00, -0.56)

Swiss Confederation	-1.70 (-1.87, -1.52)	-2.25 (-2.43, -2.07)	-3.93 (-4.29, -3.56)	-4.16 (-4.50, -3.81)	-1.70 (-1.87, -1.53)	-1.95 (-2.14, -1.77)
Syrian Arab Republic	-0.25 (-0.43, -0.08)	-0.62 (-0.79, -0.44)	-1.96 (-2.1, -1.82)	-2.30 (-2.45, -2.16)	-0.93 (-1.08, -0.78)	-1.23 (-1.38, -1.07)
Taiwan (Province of China)	-1.39 (-1.68, -1.10)	-0.97 (-1.32, -0.62)	-4.05 (-4.28, -3.81)	-4.43 (-4.65, -4.2)	-0.26 (-0.53, 0.02)	-0.42 (-0.66, -0.19)
Togolese Republic	1.85 (1.65, 2.04)	1.99 (1.79, 2.19)	-1.31 (-1.41, -1.22)	-1.18 (-1.28, -1.08)	-0.42 (-0.47, -0.36)	-0.29 (-0.34, -0.24)
Tokelau	-1.14 (-1.24, -1.05)	-1.10 (-1.18, -1.01)	-2.27 (-2.35, -2.2)	-2.19 (-2.26, -2.11)	-0.66 (-0.70, -0.61)	-0.59 (-0.65, -0.54)
Turkmenistan	-4.90 (-5.38, -4.42)	-5.15 (-5.61, -4.69)	-3.93 (-4.1, -3.75)	-4.13 (-4.32, -3.95)	-1.66 (-2.02, -1.29)	-1.79 (-2.16, -1.43)
Tuvalu	-0.84 (-0.90, -0.78)	-0.84 (-0.90, -0.77)	-1.50 (-1.59, -1.41)	-1.48 (-1.58, -1.38)	-0.21 (-0.24, -0.18)	-0.23 (-0.26, -0.19)
Ukraine	-1.91 (-2.06, -1.75)	-1.90 (-2.06, -1.74)	-4.33 (-4.6, -4.06)	-4.45 (-4.72, -4.17)	-1.63 (-1.92, -1.34)	-1.79 (-2.08, -1.51)
Union of the Comoros	-2.11 (-2.32, -1.91)	-2.35 (-2.59, -2.11)	-2.39 (-2.56, -2.23)	-2.61 (-2.81, -2.40)	-0.34 (-0.46, -0.23)	-0.65 (-0.82, -0.49)
United Arab Emirates	0.68 (0.07, 1.30)	-0.41 (-1.01, 0.19)	-1.62 (-2.24, -1.00)	-2.64 (-3.23, -2.03)	-0.43 (-0.99, 0.13)	-1.44 (-1.97, -0.90)
United Kingdom of Great Britain and Northern Ireland	-1.22 (-1.44, -1.01)	-1.47 (-1.7, -1.24)	-4.74 (-4.84, -4.65)	-4.95 (-5.05, -4.86)	-3.00 (-3.12, -2.88)	-3.01 (-3.12, -2.89)
United Mexican States	-3.58 (-3.67, -3.49)	-3.31 (-3.39, -3.22)	-4.29 (-4.44, -4.13)	-3.99 (-4.15, -3.83)	-0.55 (-0.66, -0.44)	-0.24 (-0.36, -0.12)
United Republic of Tanzania	-2.79 (-3.00, -2.58)	-2.84 (-3.06, -2.62)	-2.87 (-3.06, -2.68)	-2.87 (-3.06, -2.67)	-0.56 (-0.64, -0.47)	-0.59 (-0.68, -0.51)
United States of America	-0.94 (-1.12, -0.75)	-1.24 (-1.42, -1.06)	-3.27 (-3.33, -3.21)	-3.34 (-3.39, -3.28)	-2.30 (-2.39, -2.22)	-2.10 (-2.17, -2.03)
United States Virgin Islands	-1.81 (-2.02, -1.61)	-1.80 (-1.98, -1.62)	-3.24 (-3.49, -2.99)	-3.22 (-3.44, -3.00)	-1.68 (-2.00, -1.37)	-1.60 (-1.88, -1.33)

GI: gastrointestinal; DALYs: disability-adjusted life years; ASMR: age-standardized mortality rates; ASDR: age-standardized DALYs rates; EAPC: estimated annual percentage change.

Table S6: The DALYs of colorectal cancer attributable to smoking in males by SDI region, from1990 to 2021

Year	High-middle SDI	High SDI	Low-middle SDI	Middle SDI	Low SDI	al_Global	upper_Global	lower_Global
1990	228380.56	233407.07	38991.91	167841.77	9415.26	34.96	47.16	22.22
1991	232579.29	236532.82	39568.71	171883.38	9513.53	34.75	47.73	22.15
1992	238994.76	239095.46	40449.39	175626.85	9594.77	34.63	47.08	22.07
1993	247638.15	240493.90	41443.06	179255.80	9741.51	34.55	46.77	22.25
1994	253214.13	241294.12	42408.26	181649.19	9892.57	34.25	46.94	22.34
1995	254326.40	241967.83	43409.16	184970.90	9947.08	33.83	46.50	21.46
1996	254369.78	241025.07	44289.13	187681.48	10012.91	33.24	45.87	21.94
1997	253965.10	240250.16	45424.92	188611.50	10215.80	32.57	44.27	21.11
1998	254909.79	240099.03	46299.20	190890.29	10336.73	32.03	43.62	20.78
1999	261183.01	240108.69	46874.12	193749.04	10221.53	31.71	42.80	20.20
2000	267944.80	240139.69	48174.76	198593.72	10214.84	31.52	42.78	20.05
2001	271323.27	240324.81	49512.37	203078.74	10254.28	31.16	42.34	20.41
2002	275587.89	239616.93	50732.62	208385.75	10350.53	30.81	41.65	19.93
2003	280366.12	238052.64	52199.84	214318.57	10530.94	30.50	41.56	19.68
2004	284452.65	235647.88	53247.08	221627.49	10684.90	30.13	40.86	19.16
2005	289109.43	234573.25	54569.85	226539.36	10823.86	29.76	40.54	19.15
2006	286598.31	233786.17	55785.45	228645.98	10922.36	28.98	39.17	18.57
2007	289838.19	233556.80	57528.39	235662.87	11081.73	28.62	38.81	18.33
2008	297085.82	233593.23	59237.42	246381.68	11238.63	28.54	38.60	18.37
2009	300879.21	232197.95	60759.59	255440.08	11484.75	28.22	38.69	18.19
2010	308644.69	232568.14	62573.11	266554.88	11742.27	28.16	38.11	17.77
2011	313376.57	232649.84	64331.74	275722.59	11979.98	27.91	38.35	17.37
2012	320290.14	231232.74	65316.11	284657.69	12145.13	27.63	37.75	17.42

2013	322798.85	230575.95	66465.76	291359.98	12485.75	27.19	37.51	17.04
2014	325958.03	228858.10	68038.66	297089.08	12800.58	26.73	36.81	16.68
2015	331387.22	230619.73	70169.87	304851.91	13266.68	26.53	36.15	17.35
2016	338071.53	231461.72	72510.40	313674.27	13623.61	26.35	36.28	16.86
2017	341084.32	231924.87	74638.03	320920.90	14036.47	26.01	36.16	16.54
2018	349011.64	231242.70	77467.54	330440.26	14528.92	25.85	35.47	16.08
2019	356249.32	231030.74	79816.62	342155.01	15083.10	25.74	35.87	15.88
2020	358673.31	227831.51	81626.02	350379.47	15540.26	25.37	35.55	16.01
2021	362178.19	229516.10	83058.80	357957.87	15868.73	25.20	34.99	15.92

Table S7: The DALYs of colorectal cancer attributable to smoking in females by SDI region, from1990 to 2021

Year	High-middle SDI	High SDI	Low-middle SDI	Middle SDI	Low SDI	val_Global	upper_Global	lower_Global
1990	40513.89	101741.89	7073.97	19796.30	1886.79	8.00	10.95	5.08
1991	40875.82	101491.69	7222.42	20245.15	1925.20	7.87	10.76	4.95
1992	41097.22	100930.67	7434.70	20956.86	1966.28	7.73	10.67	4.87
1993	42187.20	100991.01	7580.08	21570.21	2009.62	7.66	10.50	4.86
1994	43189.71	100235.10	7792.67	22185.72	2050.80	7.54	10.44	4.75
1995	43357.50	99783.72	7996.73	22448.39	2073.65	7.40	10.18	4.68
1996	43564.30	98590.53	8143.61	22806.20	2100.55	7.23	9.96	4.60
1997	43957.30	97801.96	8326.96	22986.98	2134.33	7.07	9.81	4.50
1998	44246.49	97368.56	8545.42	23361.07	2164.93	6.94	9.58	4.40
1999	45225.49	97518.34	8686.55	23660.07	2183.88	6.84	9.44	4.33
2000	45677.45	96865.77	8906.14	23799.28	2212.48	6.70	9.30	4.20
2001	46324.33	96682.17	9090.70	24091.16	2235.07	6.58	9.09	4.13
2002	47093.18	96073.15	9306.36	24448.51	2283.32	6.45	8.95	4.06
2003	48454.35	95580.36	9431.86	24728.90	2332.73	6.34	8.77	4.00
2004	48934.43	93911.16	9510.68	25000.76	2348.20	6.16	8.49	3.85
2005	49966.06	93356.87	9624.04	25122.10	2369.32	6.03	8.34	3.74
2006	49553.40	93189.52	9702.34	25189.30	2389.44	5.86	8.11	3.66
2007	50210.54	93103.73	9849.04	25407.15	2409.02	5.74	7.90	3.59
2008	51119.80	93078.84	9952.75	25836.93	2425.79	5.63	7.74	3.53
2009	51229.87	92593.77	9974.32	26224.03	2446.57	5.48	7.55	3.43
2010	51719.57	91604.32	10073.74	26486.35	2481.35	5.33	7.41	3.36
2011	51753.46	92021.16	10267.78	26853.27	2546.53	5.22	7.23	3.27
2012	51803.71	91662.63	10423.01	26972.36	2608.62	5.08	7.05	3.18

2013	51366.28	91057.75	10657.77	27226.08	2664.83	4.93	6.82	3.09
2014	51249.52	90639.51	10854.33	27418.83	2724.47	4.80	6.66	3.00
2015	51564.00	91319.97	11231.27	27948.35	2805.47	4.72	6.56	2.91
2016	51589.91	91810.56	11546.22	28815.75	2864.33	4.64	6.42	2.90
2017	51351.70	90543.80	11865.86	29423.43	2941.00	4.50	6.23	2.81
2018	51462.80	89642.54	12219.83	30200.35	3023.07	4.40	6.12	2.78
2019	51403.35	88348.92	12339.09	31289.56	3097.43	4.28	5.96	2.66
2020	50504.16	85779.38	12438.26	32006.15	3157.76	4.11	5.72	2.53
2021	50843.05	85990.02	12532.38	33000.24	3227.62	4.06	5.68	2.53

Table S8: The DALYs of esophageal cancer attributable to smoking in males by SDI region, from1990 to 2021

Year	High-middle SDI	High SDI	Low-middle SDI	Middle SDI	Low SDI	val_Global	upper_Global	lower_Global
1990	1177556.61	581228.56	153559.10	1420867.89	45176.91	173.77	210.22	139.43
1991	1194050.27	591007.40	155500.22	1442108.12	46000.66	172.43	206.33	137.76
1992	1212892.36	599215.91	158771.27	1451545.65	46784.33	170.59	207.04	135.29
1993	1226232.37	610995.78	161100.46	1464232.12	47926.74	168.97	202.43	135.00
1994	1230052.17	619312.00	164112.59	1467304.71	48839.21	166.44	196.81	134.52
1995	1236380.90	625036.66	167631.48	1487327.08	49299.63	164.81	195.06	134.07
1996	1234845.35	629107.38	169806.08	1483763.67	50070.53	161.53	190.79	131.10
1997	1239101.31	631445.57	172927.35	1470891.00	51407.75	158.11	187.49	128.77
1998	1252007.92	637892.64	175330.18	1473279.14	52465.31	155.95	186.04	127.57
1999	1278699.46	647804.02	175422.03	1486728.46	52202.72	154.68	182.73	124.38
2000	1332854.86	656958.52	178324.57	1515103.44	52384.26	155.41	183.70	126.92
2001	1362213.81	664964.61	181379.55	1540261.45	52489.99	154.75	185.20	123.63
2002	1386188.62	671731.36	183315.90	1562118.32	52424.71	153.32	182.67	125.82
2003	1424154.05	677871.26	185653.64	1600888.92	52849.76	153.27	182.23	124.57
2004	1470975.69	677229.12	187017.73	1647755.00	52987.23	153.17	183.52	122.84
2005	1477803.76	684364.51	190335.33	1633054.86	53190.12	149.53	176.02	121.27
2006	1440632.30	683678.70	192491.86	1577791.60	53386.36	142.19	167.31	114.76
2007	1430232.47	684248.30	196639.68	1564842.01	53567.60	137.82	162.23	111.99
2008	1431380.17	681922.35	200395.94	1583096.88	53865.78	135.12	159.68	111.83
2009	1441284.30	679883.85	202393.91	1596584.59	54421.70	132.59	158.82	109.67
2010	1453919.02	682294.99	206144.52	1612536.27	55190.83	130.35	154.96	105.11
2011	1467504.84	678615.48	208202.13	1618184.44	55898.64	127.38	153.70	103.13
2012	1492615.33	666722.95	208336.71	1631397.94	56436.37	124.66	151.49	99.15

2013	1484950.24	657861.82	209976.36	1628009.25	57425.13	120.79	145.90	96.83
2014	1464680.33	661379.14	211948.04	1620810.82	58590.09	116.94	141.23	93.90
2015	1478277.55	664515.17	215746.72	1633878.29	59967.01	114.73	138.69	92.66
2016	1502624.42	664936.18	220990.19	1657980.47	61344.82	113.15	136.99	89.55
2017	1512878.85	661150.12	226223.73	1676376.28	62814.68	110.90	136.19	87.24
2018	1553555.10	659494.91	232705.44	1717904.75	64679.55	110.17	137.08	85.39
2019	1600622.40	663643.95	238061.99	1770779.76	66822.49	110.01	139.37	85.03
2020	1640553.65	653897.13	242700.55	1821684.27	68636.32	109.42	135.47	84.34
2021	1680872.95	660834.82	245587.65	1865163.78	69939.37	109.33	136.76	83.18

Table S9: The DALYs of esophageal cancer attributable to smoking in females by SDI region, from1990 to 2021

Year	High-middle SDI	High SDI	Low-middle SDI	Middle SDI	Low SDI	val_Global	upper_Global	lower_Global
1990	70184.97	85773.72	17758.54	79789.09	7477.70	12.33	15.94	8.82
1991	71120.41	86324.58	18032.84	81106.17	7641.32	12.21	15.70	8.56
1992	72019.66	86976.61	18513.00	82402.68	7805.74	12.10	15.64	8.55
1993	73545.34	88723.14	18865.17	83495.93	7993.91	12.06	15.78	8.58
1994	74837.11	89157.09	19299.41	84987.17	8101.43	11.98	15.59	8.46
1995	75084.99	89997.31	19736.65	84806.19	8104.23	11.80	15.20	8.36
1996	74911.50	90925.22	19895.00	84006.39	8159.06	11.56	14.95	8.16
1997	75008.34	90221.52	20225.02	83000.31	8293.28	11.27	14.52	8.04
1998	75311.02	90340.15	20599.22	81886.68	8418.80	11.03	14.14	7.83
1999	75880.70	91317.15	20741.96	81070.62	8438.06	10.82	14.09	7.72
2000	77319.89	91955.98	20936.99	80815.44	8462.83	10.66	13.72	7.49
2001	77162.11	91817.70	21216.02	80761.45	8466.04	10.42	13.59	7.39
2002	76130.39	92007.46	21535.58	80351.20	8530.40	10.13	13.10	7.25
2003	76318.67	91568.67	21779.64	80121.99	8609.98	9.89	12.76	7.06
2004	76505.47	90968.70	21770.13	79668.48	8580.14	9.61	12.41	6.99
2005	75000.87	90321.32	21891.47	76907.93	8555.54	9.22	12.02	6.63
2006	71678.89	90364.87	22004.41	72671.54	8539.70	8.73	11.31	6.47
2007	69501.65	89852.62	22155.46	69796.66	8523.33	8.32	10.60	6.13
2008	67370.57	89453.64	22112.97	68019.40	8507.37	7.96	10.20	5.91
2009	65916.34	89527.89	21815.45	66835.86	8511.18	7.65	9.93	5.59
2010	64293.27	89676.93	21804.81	64959.87	8550.82	7.35	9.42	5.37
2011	62928.16	88250.20	21687.60	62746.45	8632.22	7.00	8.94	5.17
2012	60930.26	87004.97	21460.66	60049.71	8721.37	6.63	8.45	4.86

2013	59002.79	86266.73	21525.85	57775.36	8850.28	6.32	8.08	4.66
2014	57460.68	87174.41	21465.42	56521.21	8966.40	6.09	7.77	4.49
2015	57560.80	87977.13	21706.49	56195.31	9141.09	5.95	7.64	4.26
2016	58226.52	88785.49	21841.32	56738.35	9291.07	5.83	7.56	4.25
2017	58492.75	87792.41	21990.10	56763.69	9467.35	5.65	7.34	4.08
2018	59329.74	88307.13	22236.16	57517.90	9643.08	5.55	7.17	4.06
2019	60403.82	87919.09	22167.48	58609.39	9820.66	5.43	7.11	3.95
2020	61148.22	85309.01	22086.23	59944.15	9975.67	5.27	6.93	3.82
2021	61999.07	85349.98	21992.17	61296.32	10135.93	5.19	6.86	3.66

Table S10: The DALYs of gastric cancer attributable to smoking in males by SDI region, from1990 to 2021

Year	High-middle SDI	Low SDI	Low-middle SDI	High SDI	Middle SDI	val_Global	upper_Global	lower_Global
1990	1006822.22	32363.30	152092.93	535491.00	956485.83	138.96	167.45	111.93
1991	1002618.24	32621.08	152525.87	528157.24	960707.13	135.43	163.83	108.14
1992	1005302.77	32805.37	153819.43	517062.19	960604.90	132.04	160.31	105.90
1993	1006034.59	33206.38	153416.32	507579.59	960991.99	128.71	154.46	103.95
1994	996572.07	33713.75	153241.11	500083.21	953551.17	124.84	149.22	101.38
1995	982709.14	33711.18	153067.32	489461.68	956274.43	121.27	144.37	97.48
1996	959706.08	34170.83	154075.82	477276.27	945698.20	116.73	139.07	93.81
1997	939311.68	35200.19	155781.12	464374.15	928585.51	112.12	132.98	90.14
1998	930099.16	35696.22	156422.63	455950.00	921772.51	108.68	131.25	87.02
1999	937681.66	34862.58	154739.07	446265.30	919690.00	105.94	125.95	84.81
2000	954478.75	34476.87	155790.61	432092.19	929943.15	104.16	123.74	84.07
2001	957463.90	34390.26	157031.77	418351.00	941756.28	101.89	121.29	81.44
2002	960960.08	34303.75	158289.29	406226.22	957014.07	99.79	118.84	79.39
2003	966603.15	34497.73	160182.10	398882.63	975272.89	98.26	117.74	76.41
2004	974471.49	34315.93	160122.35	390055.55	996575.34	96.68	116.56	76.17
2005	962179.11	34534.17	162238.41	379352.18	986181.02	93.16	111.06	74.62
2006	918615.87	34940.09	164924.14	369091.84	951305.81	87.62	103.96	69.39
2007	897722.00	35562.92	169933.93	360380.00	947428.37	84.39	100.38	67.56
2008	894467.95	35780.47	173222.59	351952.65	961879.66	82.50	98.02	66.21
2009	891814.18	36113.09	175051.62	344294.79	971537.14	80.54	96.83	63.81
2010	891615.20	36855.09	178872.90	337788.58	978823.73	78.68	94.41	61.84
2011	885559.36	36926.45	179604.34	329004.98	978520.56	76.13	91.87	60.06
2012	890870.99	36854.17	178696.80	318145.80	985495.44	74.05	90.02	58.82

2013	880438.71	37225.79	179065.11	310444.69	979628.06	71.42	87.28	57.07
2014	864748.51	37212.23	178510.32	301454.57	971065.87	68.56	83.38	53.91
2015	863002.45	37789.53	179662.24	294429.91	974832.62	66.62	82.09	52.54
2016	867973.04	37851.82	181158.58	288965.49	982463.57	65.08	80.01	50.45
2017	856515.24	38186.52	182554.69	283411.23	974857.91	62.72	78.04	49.74
2018	857433.79	38859.67	186090.37	278501.56	975740.01	61.05	76.93	46.94
2019	862185.56	39777.51	188105.99	273458.35	984541.98	59.71	75.94	45.54
2020	866168.43	40338.17	189096.99	269162.28	992332.99	58.46	73.51	43.93
2021	871537.07	40537.25	189073.52	270863.28	1000047.16	57.57	74.59	44.84

Table S11: The DALYs of gastric cancer attributable to smoking in females by SDI region, from1990 to 2021

Year	High-middle SDI	Low SDI	Low-middle SDI	High SDI	Middle SDI	val_Global	upper_Global	lower_Global
1990	77986.18	4367.38	14212.01	93586.70	53812.84	11.45	13.80	9.28
1991	76910.78	4433.52	14326.80	91545.31	54314.70	11.08	13.13	8.94
1992	76077.64	4506.56	14566.90	89230.73	55073.56	10.75	12.75	8.75
1993	76288.82	4574.29	14637.50	87241.59	55381.76	10.46	12.49	8.44
1994	76273.55	4650.65	14746.28	85288.01	55610.90	10.17	12.12	8.21
1995	75144.77	4678.69	14840.30	83869.62	55166.07	9.84	11.83	7.94
1996	73774.01	4741.00	14950.79	81732.94	54476.30	9.47	11.38	7.76
1997	73062.45	4788.97	15175.08	79659.62	53325.83	9.12	10.88	7.39
1998	72177.96	4822.94	15389.58	78402.09	52308.90	8.80	10.49	7.12
1999	72520.04	4835.57	15399.78	77115.71	51818.68	8.55	10.22	6.95
2000	72924.94	4868.45	15398.23	75148.86	51075.55	8.28	9.88	6.65
2001	72891.75	4916.58	15447.57	73105.23	50662.24	7.99	9.54	6.49
2002	72873.66	5025.36	15630.53	71118.28	50377.04	7.73	9.15	6.26
2003	73595.78	5099.90	15611.91	69671.21	49978.54	7.51	8.99	6.13
2004	73782.20	5065.32	15483.58	68208.96	49670.05	7.27	8.65	5.87
2005	72516.30	5116.01	15563.56	66104.18	48127.34	6.93	8.27	5.60
2006	69348.67	5167.21	15599.96	64538.85	45908.43	6.53	7.81	5.34
2007	67839.65	5245.31	15735.39	63060.90	44268.00	6.22	7.38	5.04
2008	66748.26	5262.84	15579.48	61900.90	43485.87	5.95	7.14	4.87
2009	65470.67	5271.52	15392.38	60627.87	42821.55	5.70	6.84	4.64
2010	64228.51	5358.95	15539.09	59630.98	42095.58	5.47	6.56	4.46
2011	62793.65	5466.14	15508.06	58159.08	41180.60	5.22	6.22	4.23
2012	61029.02	5604.61	15534.56	56962.12	40177.11	4.97	5.98	4.03

2013	59101.31	5739.53	15675.68	55717.27	39312.44	4.74	5.70	3.83
2014	57675.72	5737.77	15612.72	54279.10	38739.56	4.52	5.42	3.61
2015	57146.23	5820.96	15853.36	53353.58	38661.20	4.37	5.32	3.52
2016	56450.24	5844.58	16024.00	52861.03	39098.40	4.23	5.13	3.42
2017	55422.89	5907.18	16094.75	51315.82	39095.73	4.06	4.92	3.29
2018	55029.59	5975.70	16346.40	50305.69	39609.70	3.94	4.71	3.16
2019	54619.28	6039.53	16326.60	49010.68	40234.88	3.81	4.69	3.07
2020	53937.16	6062.16	16210.09	47171.34	40637.00	3.66	4.55	2.91
2021	54112.92	6108.33	16092.24	47000.08	41260.81	3.59	4.42	2.88

Table S12: The deaths of colorectal cancer attributable to smoking in males by SDI region, from1990 to 2021

Year	Low SDI	Low-middle SDI	High-middle SDI	High SDI	Middle SDI	val_Global	upper_Global	lower_Global
1990	333.79	1341.85	7998.43	9067.74	5561.00	1.39	1.88	0.88
1991	337.55	1363.24	8157.51	9177.98	5708.26	1.38	1.90	0.87
1992	340.75	1397.29	8394.92	9287.41	5850.34	1.37	1.88	0.87
1993	346.07	1433.44	8703.77	9336.18	5987.94	1.37	1.86	0.88
1994	352.33	1471.41	8909.68	9362.95	6083.91	1.35	1.85	0.88
1995	354.64	1511.20	8986.87	9405.96	6218.48	1.34	1.84	0.84
1996	356.41	1543.10	9034.84	9382.46	6332.69	1.32	1.82	0.86
1997	363.22	1583.67	9071.07	9357.33	6389.69	1.29	1.78	0.83
1998	368.06	1618.92	9160.99	9376.28	6489.24	1.28	1.75	0.82
1999	363.69	1638.18	9421.33	9401.17	6592.78	1.26	1.74	0.80
2000	364.53	1685.64	9702.69	9404.95	6781.69	1.26	1.73	0.80
2001	366.50	1735.58	9865.47	9408.07	6959.42	1.24	1.71	0.81
2002	370.38	1783.59	10044.39	9403.31	7147.71	1.23	1.68	0.79
2003	377.52	1843.03	10267.82	9370.25	7372.38	1.22	1.67	0.78
2004	383.14	1885.41	10449.74	9291.24	7638.98	1.20	1.65	0.76
2005	388.20	1930.46	10644.41	9264.99	7825.30	1.19	1.63	0.76
2006	392.09	1972.29	10550.45	9231.67	7888.98	1.15	1.57	0.73
2007	397.84	2040.67	10671.69	9224.74	8152.99	1.14	1.55	0.73
2008	403.87	2103.35	10954.93	9242.52	8577.98	1.14	1.55	0.72
2009	412.91	2153.05	11133.81	9198.85	8951.91	1.12	1.55	0.72
2010	423.04	2218.40	11447.17	9227.19	9392.49	1.12	1.52	0.71
2011	432.10	2277.69	11649.02	9253.15	9742.54	1.11	1.52	0.69
2012	437.74	2306.93	11936.65	9221.54	10083.06	1.10	1.51	0.69

2013	451.82	2360.80	12047.18	9221.79	10357.17	1.08	1.50	0.67
2014	464.03	2433.17	12176.82	9178.38	10604.55	1.06	1.46	0.65
2015	481.15	2503.42	12415.67	9277.57	10927.07	1.06	1.44	0.68
2016	494.40	2588.13	12705.71	9337.56	11295.70	1.05	1.45	0.67
2017	509.98	2676.06	12825.77	9388.79	11583.60	1.04	1.45	0.65
2018	527.64	2789.57	13128.54	9407.89	11967.49	1.03	1.42	0.64
2019	548.22	2883.36	13429.40	9443.01	12445.43	1.02	1.42	0.63
2020	563.79	2951.14	13574.08	9381.97	12801.78	1.01	1.41	0.63
2021	572.04	2999.51	13724.20	9502.08	13121.66	1.01	1.40	0.63

Table S13: The deaths of colorectal cancer attributable to smoking in females by SDI region, from1990 to 2021

Year	Low SDI	Low-middle SDI	High-middle SDI	High SDI	Middle SDI	val_Global	upper_Global	lower_Global
1990	66.27	253.56	1481.51	4187.22	754.86	0.33	0.45	0.20
1991	67.72	259.22	1500.51	4187.96	774.68	0.32	0.44	0.20
1992	69.31	267.65	1516.80	4187.06	804.00	0.32	0.44	0.20
1993	70.93	274.04	1562.11	4200.06	830.24	0.31	0.43	0.20
1994	72.76	282.80	1602.74	4184.09	856.47	0.31	0.43	0.19
1995	73.82	291.96	1615.35	4173.31	872.07	0.30	0.42	0.19
1996	74.65	298.39	1630.39	4132.44	889.22	0.30	0.42	0.19
1997	75.81	305.99	1652.19	4102.34	896.88	0.29	0.40	0.18
1998	77.07	316.02	1672.34	4093.84	912.64	0.29	0.40	0.18
1999	77.96	322.17	1711.12	4101.76	926.87	0.28	0.39	0.17
2000	79.27	331.51	1735.73	4073.19	937.48	0.28	0.38	0.17
2001	80.66	339.06	1762.61	4054.91	951.96	0.27	0.38	0.17
2002	83.23	348.34	1789.34	4034.45	966.76	0.26	0.37	0.16
2003	85.28	355.52	1840.99	4013.80	978.49	0.26	0.36	0.16
2004	85.83	360.88	1864.23	3945.34	990.31	0.25	0.35	0.16
2005	86.62	366.85	1903.43	3913.92	996.15	0.25	0.35	0.15
2006	87.57	371.39	1888.54	3900.97	996.92	0.24	0.34	0.15
2007	88.62	377.80	1909.68	3890.58	1003.21	0.23	0.33	0.14
2008	89.51	382.64	1942.43	3887.70	1020.24	0.23	0.32	0.14
2009	90.59	383.54	1949.13	3860.38	1034.53	0.22	0.31	0.14
2010	92.23	387.26	1969.89	3822.10	1047.84	0.22	0.30	0.13
2011	95.20	394.81	1977.36	3845.49	1061.96	0.21	0.30	0.13
2012	98.35	401.53	1976.39	3836.06	1066.98	0.21	0.29	0.13

2013	101.47	411.79	1959.19	3819.93	1076.40	0.20	0.28	0.12
2014	103.49	420.41	1958.34	3808.87	1086.03	0.19	0.27	0.12
2015	105.94	432.51	1977.30	3837.45	1107.94	0.19	0.27	0.12
2016	108.08	443.60	1989.69	3864.75	1144.45	0.19	0.26	0.11
2017	110.96	456.53	1983.75	3829.42	1171.98	0.18	0.25	0.11
2018	113.79	471.06	1991.20	3808.49	1205.21	0.18	0.25	0.11
2019	116.33	477.01	1998.58	3779.48	1251.89	0.17	0.24	0.11
2020	118.22	480.42	1982.08	3693.80	1280.82	0.17	0.24	0.10
2021	120.35	482.02	1995.23	3717.45	1319.25	0.16	0.23	0.10

Table S14: The deaths of esophageal cancer attributable to smoking in males by SDI region, from1990 to 2021

Year	High-middle SDI	Low-middle SDI	Low SDI	High SDI	Middle SDI	val_Global	upper_Global	lower_Global
1990	42873.46	5544.97	1607.51	23349.92	51126.75	6.93	8.29	5.57
1991	43564.56	5621.77	1637.34	23824.60	52043.40	6.89	8.20	5.53
1992	44330.32	5752.10	1666.59	24195.85	52554.41	6.83	8.23	5.43
1993	44940.42	5844.52	1707.00	24739.35	53192.77	6.78	8.09	5.38
1994	45250.62	5969.53	1742.17	25170.25	53497.27	6.70	7.96	5.42
1995	45672.59	6114.60	1760.26	25496.52	54446.32	6.65	7.84	5.43
1996	45785.81	6194.36	1785.25	25721.70	54518.97	6.54	7.71	5.31
1997	46177.71	6308.10	1829.14	25893.91	54376.52	6.43	7.63	5.20
1998	46899.30	6408.63	1867.44	26292.18	54793.97	6.38	7.59	5.20
1999	48120.44	6403.35	1856.48	26776.97	55544.41	6.35	7.48	5.09
2000	50456.72	6513.12	1868.25	27231.03	57075.23	6.41	7.54	5.25
2001	51878.67	6645.81	1874.06	27673.27	58505.98	6.42	7.67	5.16
2002	52989.86	6731.84	1873.04	28027.49	59650.19	6.39	7.60	5.20
2003	54869.69	6852.85	1890.06	28390.22	61743.43	6.43	7.68	5.21
2004	56931.33	6925.23	1895.20	28479.29	63828.17	6.45	7.71	5.16
2005	57353.50	7042.25	1902.48	28844.73	63487.47	6.31	7.43	5.12
2006	55805.00	7116.75	1912.30	28900.43	61157.55	5.99	7.02	4.84
2007	55603.83	7288.39	1918.17	29006.01	60920.55	5.82	6.86	4.70
2008	55999.63	7434.69	1928.62	29064.55	62324.48	5.74	6.78	4.72
2009	56966.37	7500.08	1947.58	29153.46	63771.50	5.68	6.79	4.68
2010	57894.09	7649.67	1975.58	29414.90	65040.09	5.62	6.70	4.51
2011	58671.94	7721.74	2000.92	29389.75	65516.30	5.50	6.61	4.45
2012	59926.50	7712.32	2017.64	29022.41	66257.90	5.39	6.51	4.32

2013	60040.49	7824.47	2056.95	28874.95	66573.82	5.26	6.32	4.22
2014	59618.64	7958.50	2100.25	29220.18	66666.65	5.12	6.18	4.10
2015	60528.76	8077.51	2149.18	29567.84	67529.11	5.04	6.06	4.07
2016	61914.95	8264.64	2196.48	29843.26	68945.62	4.99	6.06	3.93
2017	62689.92	8490.95	2248.60	29902.73	70030.81	4.91	6.03	3.87
2018	64650.42	8768.59	2315.25	30045.31	72125.19	4.89	6.06	3.81
2019	66963.10	8996.86	2394.17	30447.39	74778.23	4.90	6.16	3.81
2020	69064.43	9172.32	2455.58	30221.55	77347.49	4.89	6.01	3.78
2021	71135.98	9266.88	2489.88	30725.64	79595.25	4.90	6.13	3.74

Table S15: The deaths of esophageal cancer attributable to smoking in females by SDI region, from1990 to 2021

Year	High-middle SDI	Low-middle SDI	Low SDI	High SDI	Middle SDI	val_Global	upper_Global	lower_Global
1990	3260.58	678.50	277.83	4211.99	3458.78	0.58	0.75	0.41
1991	3320.33	689.93	284.47	4260.60	3535.19	0.58	0.74	0.41
1992	3387.11	711.70	291.34	4331.65	3612.20	0.57	0.75	0.41
1993	3477.41	727.79	298.82	4444.49	3686.46	0.57	0.75	0.40
1994	3564.01	749.01	304.50	4495.14	3776.38	0.57	0.75	0.40
1995	3609.54	771.70	306.12	4558.55	3800.69	0.57	0.73	0.40
1996	3629.71	779.30	307.84	4636.13	3790.72	0.56	0.73	0.39
1997	3665.78	793.17	312.71	4618.58	3758.26	0.55	0.71	0.39
1998	3709.30	812.71	317.58	4646.04	3738.88	0.54	0.70	0.38
1999	3766.55	819.20	319.10	4706.33	3727.21	0.53	0.70	0.37
2000	3882.45	828.56	321.29	4743.46	3749.47	0.53	0.69	0.36
2001	3894.85	843.92	323.20	4741.18	3786.09	0.51	0.67	0.36
2002	3857.01	861.83	328.21	4754.64	3789.95	0.50	0.66	0.35
2003	3889.95	879.73	332.15	4729.46	3805.08	0.49	0.63	0.35
2004	3930.36	886.20	331.50	4687.18	3801.66	0.48	0.63	0.34
2005	3878.55	897.11	331.28	4645.06	3680.33	0.46	0.61	0.33
2006	3705.37	907.74	331.76	4651.01	3462.75	0.43	0.57	0.31
2007	3596.39	917.72	332.43	4628.43	3324.00	0.41	0.54	0.30
2008	3501.13	918.83	333.05	4621.05	3255.44	0.40	0.52	0.28
2009	3430.46	905.22	333.92	4618.26	3212.52	0.38	0.51	0.27
2010	3362.67	903.13	336.29	4643.83	3134.06	0.37	0.48	0.26
2011	3305.93	897.23	340.87	4582.19	3033.51	0.35	0.45	0.25
2012	3192.13	888.90	346.33	4543.11	2891.25	0.33	0.43	0.24

2013	3087.60	896.17	354.37	4512.28	2774.85	0.32	0.41	0.23
2014	3018.60	896.01	359.09	4570.02	2709.08	0.30	0.40	0.22
2015	3034.80	900.79	364.98	4622.89	2694.97	0.30	0.39	0.21
2016	3092.73	904.04	370.87	4679.44	2727.91	0.29	0.38	0.21
2017	3110.40	911.70	378.15	4632.71	2731.89	0.28	0.37	0.20
2018	3159.53	922.39	384.76	4673.29	2778.70	0.28	0.37	0.20
2019	3229.66	921.08	391.69	4665.01	2841.64	0.27	0.36	0.20
2020	3286.52	915.94	397.23	4550.80	2910.06	0.26	0.35	0.19
2021	3335.41	907.32	402.29	4551.95	2976.99	0.26	0.35	0.18

Table S16: The deaths of gastric cancer attributable to smoking in males by SDI region, from1990 to 2021

year	Low SDI	Low-middle SDI	High-middle SDI	High SDI	Middle SDI	val_Global	upper_Global	lower_Global
1990	1136.47	5353.75	36582.48	22688.65	33670.71	5.66	6.84	4.58
1991	1147.84	5380.90	36479.77	22441.05	33913.14	5.52	6.68	4.45
1992	1157.03	5441.09	36619.53	22080.21	34029.48	5.39	6.55	4.33
1993	1173.28	5440.49	36681.90	21759.44	34151.46	5.26	6.36	4.30
1994	1196.65	5453.06	36400.03	21525.51	34023.78	5.11	6.12	4.21
1995	1199.54	5467.83	36014.42	21194.44	34239.47	4.98	5.94	4.05
1996	1214.17	5506.14	35329.04	20776.15	34012.03	4.81	5.72	3.89
1997	1249.30	5574.94	34742.80	20335.33	33564.36	4.64	5.55	3.77
1998	1271.15	5616.60	34576.65	20061.70	33461.49	4.51	5.43	3.64
1999	1241.32	5555.65	34991.72	19739.94	33493.91	4.41	5.27	3.55
2000	1234.17	5605.31	35824.15	19220.94	34088.34	4.35	5.17	3.51
2001	1235.79	5673.46	36133.12	18717.29	34738.62	4.27	5.09	3.45
2002	1235.81	5743.86	36408.85	18297.01	35458.59	4.19	5.00	3.33
2003	1248.10	5846.72	36939.13	18052.65	36418.02	4.15	4.98	3.26
2004	1243.69	5866.10	37476.52	17753.17	37361.82	4.10	4.94	3.23
2005	1252.47	5944.67	37133.37	17357.80	37089.42	3.95	4.73	3.15
2006	1271.39	6052.04	35515.50	16964.70	35693.63	3.71	4.40	2.94
2007	1296.63	6268.25	34860.13	16669.51	35699.33	3.59	4.30	2.85
2008	1307.15	6402.42	34942.66	16360.19	36620.81	3.52	4.19	2.83
2009	1321.04	6466.96	35205.55	16090.01	37480.52	3.46	4.17	2.74
2010	1352.59	6614.83	35445.67	15883.52	38126.03	3.40	4.09	2.66
2011	1357.11	6634.40	35389.08	15556.12	38268.17	3.30	3.97	2.58
2012	1352.97	6596.13	35776.73	15114.22	38683.00	3.21	3.88	2.56

2013	1375.36	6674.59	35586.25	14863.44	38730.77	3.11	3.78	2.48
2014	1378.33	6708.79	35132.97	14547.73	38627.97	3.00	3.64	2.35
2015	1397.08	6715.55	35239.78	14290.60	38968.86	2.92	3.59	2.31
2016	1399.93	6761.99	35637.44	14110.57	39521.15	2.86	3.52	2.22
2017	1412.15	6844.68	35334.84	13968.49	39389.79	2.76	3.44	2.17
2018	1433.20	7007.57	35498.35	13831.10	39613.18	2.69	3.40	2.07
2019	1467.63	7109.11	35867.16	13697.45	40194.97	2.64	3.33	1.99
2020	1485.79	7153.23	36247.16	13590.03	40741.12	2.59	3.24	1.96
2021	1483.35	7144.32	36612.70	13777.81	41255.38	2.56	3.30	1.99

Table S17: The deaths of gastric cancer attributable to smoking in females by SDI region, from1990 to 2021

Year	Low SDI	Low-middle SDI	High-middle SDI	High SDI	Middle SDI	val_Global	upper_Global	lower_Global
1990	150.95	521.07	3140.49	4302.39	2183.74	0.50	0.61	0.40
1991	153.67	526.78	3119.69	4228.60	2215.57	0.49	0.58	0.39
1992	156.59	537.81	3101.63	4152.48	2253.65	0.47	0.57	0.38
1993	159.11	542.67	3121.47	4085.05	2282.01	0.46	0.56	0.37
1994	162.56	548.59	3131.04	4021.82	2301.35	0.45	0.55	0.36
1995	164.20	555.62	3098.49	3970.45	2297.12	0.44	0.53	0.35
1996	165.84	561.10	3063.75	3886.28	2279.32	0.42	0.52	0.34
1997	167.36	570.36	3053.37	3805.68	2238.90	0.41	0.49	0.33
1998	168.79	582.20	3033.99	3763.54	2209.88	0.40	0.48	0.32
1999	169.62	584.97	3059.53	3708.88	2199.00	0.39	0.47	0.31
2000	171.49	586.45	3106.13	3620.56	2181.59	0.37	0.46	0.30
2001	175.07	590.33	3116.17	3532.44	2177.28	0.36	0.44	0.29
2002	181.71	600.65	3117.56	3457.19	2173.26	0.35	0.43	0.28
2003	185.27	605.30	3158.36	3388.82	2168.66	0.34	0.42	0.27
2004	183.88	603.85	3176.36	3316.81	2160.79	0.33	0.40	0.26
2005	185.82	611.00	3121.61	3220.82	2093.75	0.31	0.38	0.25
2006	188.18	616.43	2976.45	3149.35	1984.26	0.30	0.36	0.24
2007	192.50	624.12	2899.85	3085.64	1909.10	0.28	0.34	0.22
2008	193.95	618.39	2851.92	3043.31	1878.80	0.27	0.33	0.22
2009	194.97	611.98	2807.07	2990.72	1856.52	0.26	0.32	0.21
2010	199.29	617.94	2759.90	2953.15	1829.80	0.25	0.30	0.20
2011	204.89	616.28	2703.34	2899.11	1790.43	0.24	0.29	0.19
2012	213.22	618.88	2621.93	2853.36	1741.81	0.22	0.28	0.18

2013	223.10	628.55	2544.91	2801.92	1705.20	0.21	0.26	0.17
2014	222.32	627.73	2489.29	2746.30	1680.73	0.20	0.25	0.16
2015	222.51	633.24	2471.62	2709.83	1676.55	0.20	0.24	0.16
2016	222.48	637.07	2461.68	2694.00	1699.64	0.19	0.24	0.15
2017	224.19	641.08	2434.89	2639.96	1704.59	0.18	0.23	0.15
2018	225.29	652.67	2430.24	2605.44	1733.04	0.18	0.22	0.14
2019	226.31	652.93	2427.23	2553.17	1766.96	0.17	0.22	0.14
2020	225.80	647.30	2422.36	2472.88	1788.78	0.17	0.21	0.13
2021	226.59	639.50	2434.03	2477.37	1818.09	0.16	0.20	0.13

© 2026 Yang J. et al.
